# Metallophilicity Under Confinement: The Iminopyrrole Cage Promoting the Hg^II^···M^II^ (M = Hg, Cd) Interactions

**DOI:** 10.1021/acs.inorgchem.6c00290

**Published:** 2026-03-05

**Authors:** Aleksandra Sarwa, Piotr Krajewski, Bartosz Trzaskowski, Jędrzej P. Perdek, Miłosz Siczek, Bartosz Szyszko

**Affiliations:** † Faculty of Chemistry, 49572 University of Wrocław, F. Joliot-Curie 14, 50-383 Wrocław, Poland; ‡ Centre of New Technologies, 427050 University of Warsaw, Banacha 2C, 02-097 Warszawa, Poland

## Abstract

Self-assembly of 2,5-diformylpyrrole, tris­(2-aminoethyl)­amine, and Cd­(II) or Hg­(II) affords homobimetallic cryptates. In contrast, the synthesis of heterobimetallic Zn­(II)–Cd­(II) and Cd­(II)–Hg­(II) cages requires a stepwise approach involving a monocadmium­(II) precursor. The bis-mercury­(II) cryptate exhibits a pronounced mercurophilic interaction, as evidenced by a short Hg···Hg separation of 3.0549(7) Å. Notably, a similarly close intracavity contact is observed for the Cd­(II)–Hg­(II) system, with a M···M distance of ca. 3.1 Å. The participation of the metal centers in the attractive interaction—enforced by confinement within the iminopyrrole cage—was verified through the density functional theory (DFT) calculations of bond indices and was further corroborated by QTAIM analysis.

## Graphical abstract

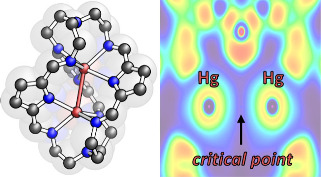

The term *metallophilicity* describes the interaction between closed-shell or pseudoclosed-shell metal cations.^1–4^ While short-range Coulombic repulsion would normally be expected between positively charged ions, multiple M···M interactions stabilize pairs and aggregates of metal cations. Although metallophilic interactions (MIs) are considerably weaker than M–M covalent bonds,^5^ they are widespread among elements such as Au, Ag, Cu, Hg, Pd, and Pt.^1,6^ MIs played a key role in stabilizing uncommon conformations,^7,8^ affecting molecular dynamics,^9,10^ while also inducing intriguing photophysical properties^11,12^ and electrical conductivity.^13^

While MIs are now recognized to play an important role in self-assembly,^14–16^ catalysis,^17–19^ and molecular sensing,^20,21^ the debate over their physical origin remains open.^22^ Several explanations have been proposed to account for the attractive forces between closed-shell metal cations, but the prevailing view attributes metallophilicity to dispersive forces acting in concert with relativistic effects.^23–26^ While MIs between the same metal centers are the most commonly observed, a growing number of heteronuclear contacts have been reported in recent years.^27–33^ Although the carefully designed coordination environment promoted close contacts between metal cations,^34–42^ the majority of MIs described to date were identified post-factum.

Our group has recently employed the iminopyrrole motif to construct metallosupramolecular assemblies.^43–46^ Consequently, it was demonstrated that the features of the iminopyrrole cage^47–49^ promoted the incorporation of silver clusters Ag_*n*_^*n*+^ (*n* = 3–5), with Ag^+^ interacting through argentophilic interactions.^43^ Herein, we demonstrate how molecular confinement can be exploited to promote close contacts between Cd­(II) and Hg­(II) in homobimetallic and heterobimetallic systems.

The homobinuclear cages were obtained by refluxing 2,5-diformylpyrrole **1**, tris­(2-aminoethyl)­amine **2** (*tren*), and diisopropylethylamine (DIPEA) in *n*-butanol or chloroform, in the presence of excess cadmium­(II) acetate (**3–Cd**_**2**_, 72%) or mercury­(II) triflate (**3-Hg**_**2**_, 26%) (Scheme 1). The analogous **3-Zn**_**2**_ was prepared following our previously reported protocol.^44^ The resulting acetate was converted to the corresponding hexafluorophosphate by dissolution in methanol and the addition of an excess of TBAPF_6_, which led to the precipitation of the product.

**Scheme 1:**
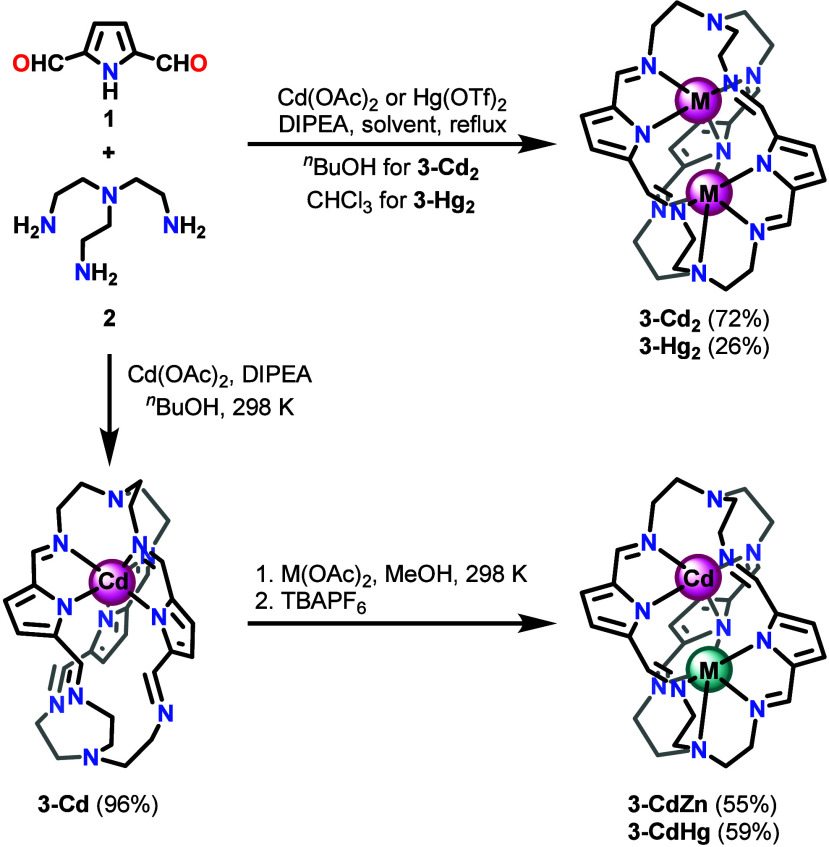
Synthesis of **3-M**_**2**_ and **3-M** ^**1**^ **M** ^**2**^

The elemental composition of **3-M**_**2**_ was determined by electrospray ionization mass spectrometry (ESI-MS), which implied that the incorporation of two cations required the deprotonation of three pyrroles (Figures S40–S45). The ^1^H NMR spectrum of **3-Zn**_**2**_ displayed a single imine resonance at 8.08 ppm, accompanied by a β-pyrrolic signal at 6.65 ppm, while methylene resonances clustered in the 3.05–2.95 and 2.62–2.50 ppm regions (Figure 1A). The spectra of **3-Cd**_**2**_ and **3-Hg**_**2**_ exhibited analogous resonance patterns, while imine resonances at 8.18 and 8.34 ppm demonstrated well-resolved ^111/113^Cd and ^199^Hg satellites, respectively (Figure 1B and 1C).^50^

**Figure 1:**
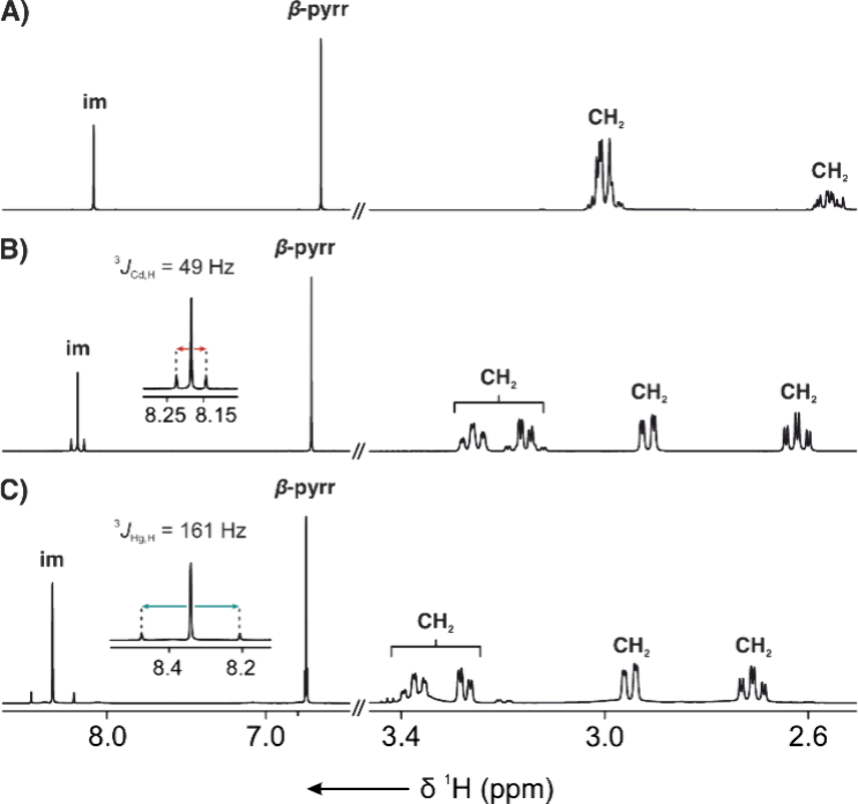
^1^H NMR spectrum of (A) **3-Zn**_**2**_, (B) **3-Cd**_**2**_, and (C) **3-Hg**_**2**_ (CD_3_CN, 300 K).

The incorporation of two metals in the cavity of the [3 + 2] cage was confirmed by SCXRD (Figure 2). In all cages, the metal cations exhibited a similar binding mode, coordinating to three imine nitrogens (M–N_im_: 2.034(2)–2.091(2) Å (**3-Zn**_**2**_), 2.212(8)–2.306(7) Å (**3-Cd**_**2**_), and 2.243(4)–2.518(5) Å (**3-Hg**_**2**_)). Both metals were also bound by one or two pyrrolides formed upon deprotonation of the pyrrole(s) (M–N_pyrr_: 2.234(2)–2.410(2) Å (**3-Zn**_**2**_), 2.360(7)–2.525(8) Å (**3-Cd**_**2**_), and 2.217(4)–2.514(5) Å (**3-Hg**_**2**_)). In **3-Zn**_**2**_, one pyrrolic ring acted as a bridging ligand, coordinating to both metal centers at 2.299(2) and 2.410(2) Å, respectively.

**Figure 2:**
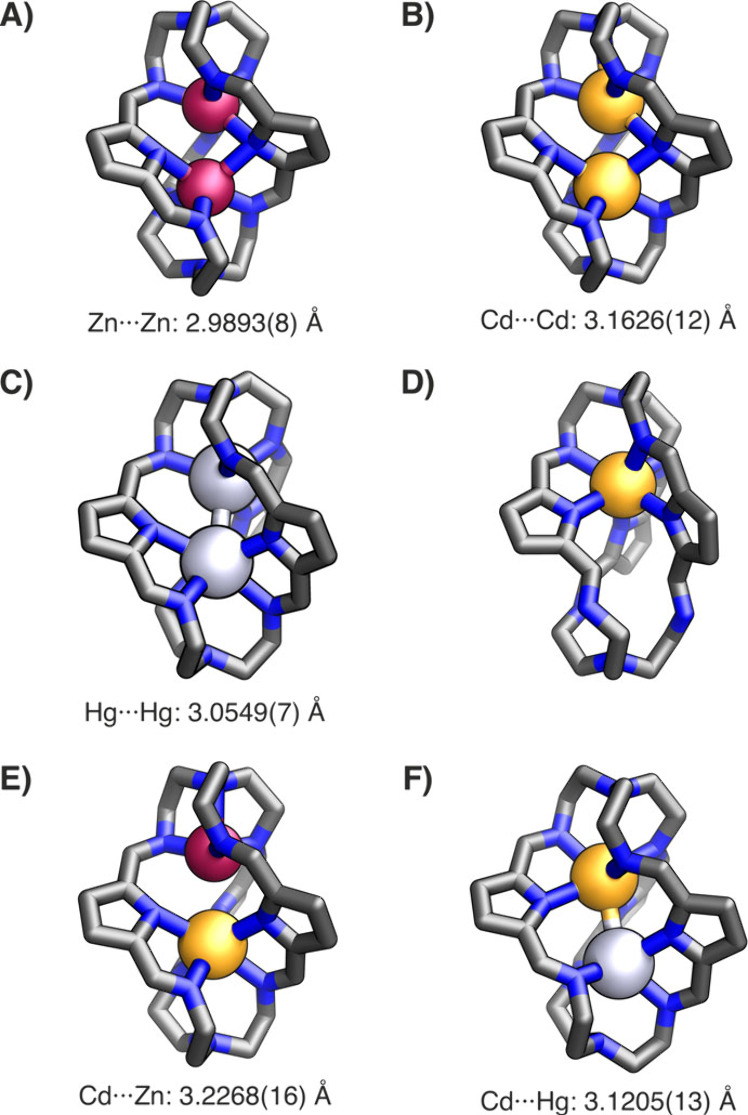
X-ray molecular structure of (A) **3-Zn**_**2**_, (B) **3-Cd**_**2**_, (C) **3-Hg**_**2**_, (D) **3-Cd**, (E) **3-CdZn**, and (F) **3-CdHg**.

Because the separation between metal centers (*d*(M···M)) is a key determinant of MIs, the intermetallic distances in **3-M**_**2**_ were determined and compared with the sum of their van der Waals radii (*r*_vdW_; Zn: 1.39, Cd: 1.62, Hg: 1.70 Å).^51^ The *d*(Zn···Zn) in **3-Zn**_**2**_ was determined to be 2.9893(8) Å, which is significantly greater than the sum of van der Waals radii of zinc atoms. In **3-Cd**_**2**_, *d*(Cd···Cd) of 3.1626(12) Å, falls in the 2*r*_vdW_ limit, while the value 3.0549(7) Å in **3-Hg**_**2**_ was significantly shorter than 2*r*_vdW_, implying a significant, ligand-supported mercurophilic interaction.^3,52^

The attempted syntheses of heterobimetallic cages using two different metal salts in the reaction resulted in mixtures of products that could not be effectively separated. Hence, the synthesis of **3-M** ^**1**^ **M** ^**2**^ required the use of a precursor cage containing a single metal cation, which could subsequently be transformed to the heterobimetallic product. Hence, the reaction of **1** and **2** with cadmium­(II) acetate (1 equiv) in *n*-BuOH was carried out, yielding **3-Cd** in 96% yield (Scheme 1).

The elemental composition of **3-Cd** was confirmed by ESI-MS (Figure S46). The ^1^H NMR spectrum exhibited six HC=N resonances (Figure S18). Three lines at 8.23, 8.21, and 8.10 ppm showed well-resolved satellites (^3^ *J*_Cd,H_ = 32 Hz), whereas the remaining singlets at 7.23, 6.29, and 6.13 ppm suggested that the corresponding imines were not involved in metal binding. The six β-pyrrolic signals appeared between 7.40 and 6.58 ppm, followed by methylene multiplets spanning 4.16–2.67 ppm. SCXRD confirmed the incorporation of a single Cd­(II) within the cage (Figure 2D). The six-coordinate metal was positioned near one pole of the cage and coordinated to three iminopyrrolides (Cd–N_im_: 2.3631(19)–2.3809(19) Å; Cd–N_pyrr_: 2.3561(18)–2.4043(18) Å). Of the three imine nitrogen atoms not involved in metal coordination, two were protonated. A single acetate compensating for the charge of the cationic cryptate was present in the asymmetric unit.

**3-Cd** proved to be a suitable precursor for heterobimetallic cryptates (Scheme 1). The reaction of **3-Cd** with Zn­(OAc)_2_ or Hg­(OAc)_2_ in methanol, followed by anion exchange to hexafluorophosphate, afforded **3-CdZn** and **3-CdHg** in 55% and 59% yield, respectively. The formation of heterobimetallic cages was confirmed by ESI-MS (Figures S48–S51).

The incorporation of two different metals in the cavity of **3-CdM** reduced molecular symmetry compared to **3-Cd**_**2**_. Accordingly, the ^1^H NMR spectrum of **3-CdZn** exhibited two imine resonances at 8.15 (^3^ *J*_Cd,H_ = 43 Hz) and 8.13 ppm, together with β-pyrrolic signals at 6.73 and 6.65 ppm (Figure 3A). Similarly, **3-CdHg** displayed a characteristic splitting pattern of the imine resonances, arising from the H–M coupling (Figure 3B). The coupling constants of ^3^ *J*_Hg,H_ = 170 Hz, and ^3^ *J*_Cd,H_ = 45 Hz were indicative of the introduction of Cd­(II) and Hg­(II) into the cavity.

**Figure 3:**
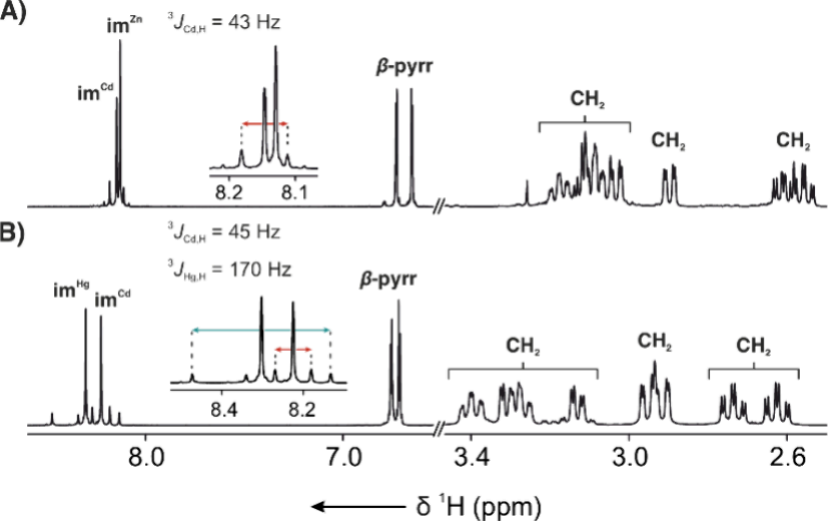
^1^H NMR spectrum of (A) **3-CdZn** and (B) **3-CdHg** (CD_3_CN, 300 K).

The smaller Zn­(II) in **3-CdZn** occupied the space available in the cavity of **3-Cd** without perturbing the coordination environment of Cd­(II) (Figure 2E). Accordingly, the six-coordinate Cd­(II) was bound by three pairs of iminopyrrolides (Cd–N_im_: 2.333(5)–2.374(5) Å; Cd–N_pyr_: 2.407(5)–2.487(5) Å), with the Cd–N bond distances essentially unaltered, compared to **3-Cd**. The three remaining imine donors, together with a bridgehead nitrogen, coordinated Zn­(II), positioning it close to the pole (Zn–N_im_: 1.927(5)–1.982(5) Å), while the apical position was occupied by the bridgehead nitrogen (Zn–N_brh_ = 2.342(5)/2.369(5) Å). The M···M separation exceeded 3.2 Å, precluding any significant attractive interaction between Cd­(II) and Zn­(II).

In contrast, incorporation of the larger Hg­(II) into the cavity of **3-Cd** led to a rearrangement of the donor positions around the metal centers (Figure 2F). The two metals were disordered within the cavity, precluding their unambiguous assignment. Notably, both cations exhibited coordination modes similar to homobimetallic **3-Cd**_**2**_ and **3-Hg**_**2**_. Specifically, one center was coordinated by three imine donors and one pyrrolide nitrogen atom, whereas the second cation bound to three imine nitrogens and two pyrrolides. The intermetallic separation ranged from 3.1205(13) (77% component of the disorder) to 3.147(4) Å (23%), intermediate between the distance observed in **3-Cd**_**2**_ (3.1626(12) Å) and **3-Hg**_**2**_ (3.0549(7) Å).

Considering that molecular geometry, flexibility, and bonding directionality may influence the M···M distance, the crystallographic studies were supported by theoretical calculations.^2–4,53^ To enable computational analysis of the cationic cryptate geometries derived from the SCXRD structures were optimized at the DFT level of theory (ωB97X-D4/def2-TZVP). For **3-Hg**_**2**_, the geometry optimization was performed in the absence of a counterion (**3-Hg**_**2**_^**+**^) and with a triflate counterion (**3-Hg**_**2**_^**OTf**^), with the latter showing substantially improved agreement with the experimental geometry (see the Supporting Information (SI)).

The bond orders for M···M pairs in **3-M**_**2**_ were determined computationally using Mayer, Wiberg, fuzzy, and Mulliken indices (Table 1). For **3-Zn**_**2**_ and **3-Cd**_**2**_, a weak M···M interaction could be inferred, as reflected by the WBI of 0.13 and 0.11, respectively. However, the negative Mulliken indices for **3-Zn**_**2**_ (−1.32) and **3-Cd**_**2**_ (−0.35) indicated a dominant antibonding character of the interaction between metals. In **3-Hg**_**2**_, the higher calculated WBI of 0.18 (**3-Hg**_**2**_^**+**^) and 0.22 (**3-Hg**_**2**_^**OTf**^), together with the increased, minimally positive Mulliken index (0.05), were consistent with the presence of a mercurophilic interaction. In the case of the heterobimetallic **3-CdZn**, only a weak M···M interaction could be inferred based on the WBI of 0.12, while in the case of **3-CdHg**, the value was minimally higher (0.15).

**Table 1:** Bond Indices Calculated for Bimetallic Cages

			Bond index			
Cage	M(1)	M(2)	Mayer	Wiberg	fuzzy	Mulliken
**3-Zn**_**2**_ ^a^	Zn	Zn	0.11	0.13	0.13	–1.32
**3-Cd**_**2**_ ^a^	Cd	Cd	0.06	0.11	0.18	–0.35
**3-Hg**_**2**_ ^a^	Hg	Hg	0.09	0.18	0.20	0.05
**3-Hg**_**2**_^**OTf**^	Hg	Hg	0.10	0.22	0.26	0.05
**3-CdZn** ^a^	Zn	Cd	0.06	0.12	0.13	–0.86
**3-CdHg** ^a^	Cd	Hg	0.06	0.15	0.21	–0.10

a Calculations were performed for **[3-M**_**2**_ **]** ^**+**^ without charge-compensating anion.

To further investigate the M···M interactions, a topological analysis of the electron density was performed using the quantum theory of atoms in molecules (QTAIM) (Figure 4, Table 2). For **3-Zn**_**2**_ and **3-Cd**_**2**_ cages, no bond critical points (CPs) were identified between the metal centers, as no stable saddle points in the electron-density topology were found. In these cases, the absence of AIM critical points reflected a nonzero density gradient (|∇ρ| ≠ 0). Moderate values of the reduced density gradient (RDG) and a positive Laplacian of the electron density (∇^2^ρ) indicated electron-density depletion in the intermetallic region, consistent with weak, noncovalent interactions. For **3-Zn**_**2**_, the positive value of the product of the local electron density and the sign of the second eigenvalue of the electron-density Hessian, sign­(λ_2_)­ρ, suggested a repulsive or weakly dispersive interaction. In contrast, the negative value of this quantity in **3-Cd**_**2**_ indicated an overall attractive interaction. Contrarily, in **3-Hg**_**2**_, a CP between the metal centers was identified (Figure 4C). The increased electron density in the intermetallic region, together with a positive Laplacian of the electron density (∇^2^ρ) and a negative value of sign­(λ_2_)­ρ, was consistent with the mercurophilic interaction.

**Figure 4:**
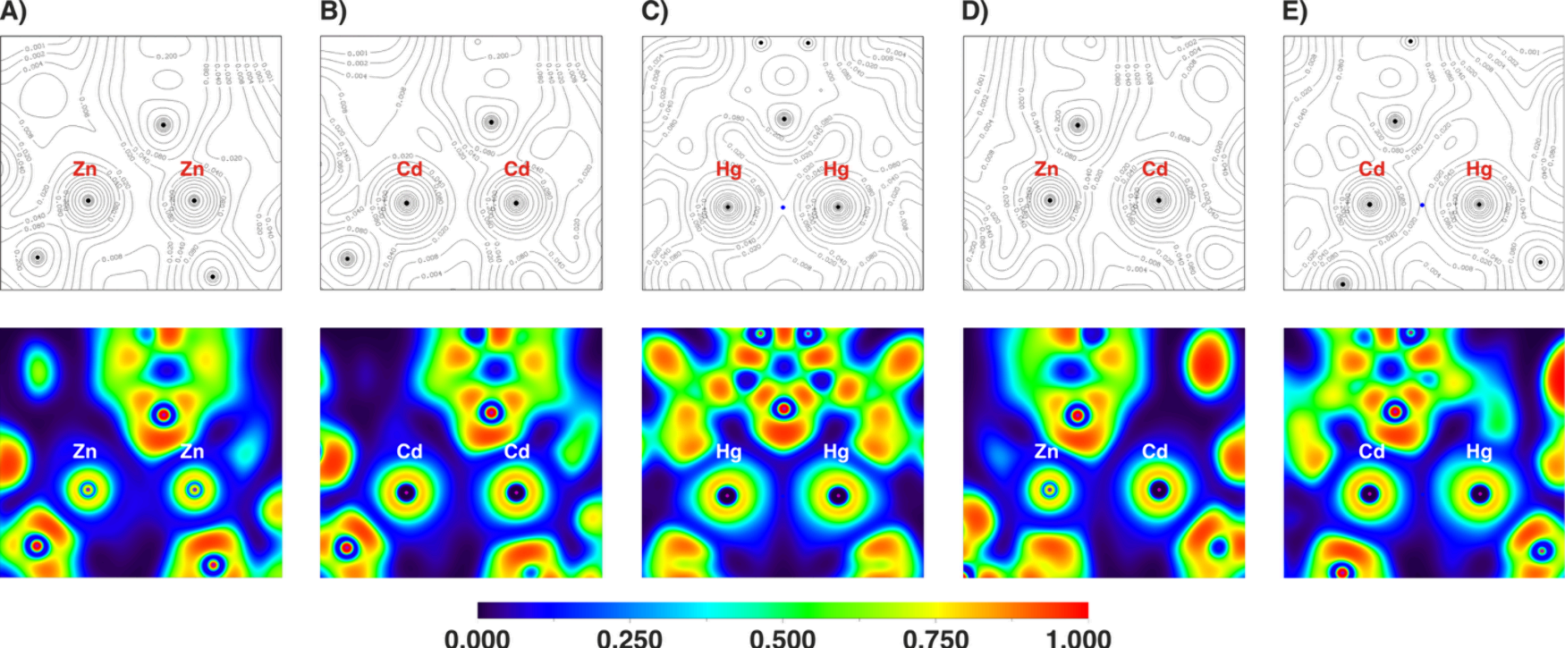
View of computed electron density map of (A) **3-Zn**_**2**_, (B) **3-Cd**_**2**_, (C) **3-Hg**_**2**_, (D) **3-CdZn**, and (E) **3-CdHg**.

**Table 2:** QTAIM Parameters (in a.u.) Calculated for Bimetallic Cages

	**Cage**				
Parameter	**3-Zn**_**2**_	**3-Cd**_**2**_	**3-Hg**_**2**_	**3-CdZn**	**3-CdHg**
**CP M(1)–M(2)**	–	–	+	–	+
**|∇ρ|**	0.0049	0.0022	8.5 × 10^–17^	0.0071	5.3 × 10^–17^
**ρ(r)**	0.016	0.017	0.021	0.015	0.019
**RDG**	0.20	0.084	2.3 × 10^–15^	0.32	1.7 × 10^–15^
**∇** ^**2**^ **ρ**	0.033	0.051	0.064	0.040	0.058
**sign(λ**_**2**_ **)·ρ**	+	–	–	–	–

The QTAIM analysis was also performed for heterobimetallic cages. For **3-CdZn**, a M···M bond CP was not detected, as the density gradient did not vanish. Higher values of RDG and ∇^2^ρ relative to **3-Zn**_**2**_ indicated significantly weaker noncovalent interactions and an overall unfavorable effect of the Zn → Cd substitution. However, the negative sign of (λ_**2**_)·ρ suggested an attractive character of the interaction between the metal centers, similar to that of the homometallic **3-Cd**_**2**_ cage. On the other hand, in **3-CdHg**, a CP between the Cd­(II) and Hg­(II) ions was detected (Figure 4E). Moreover, the values and signs of the QTAIM parameters were comparable to those found for **3-Hg**_**2**_, indicating a similar M···M interaction.

The iminopyrrole cage was shown to function as an effective ligand for metals forming mononuclear and binuclear coordination compounds of Zn­(II), Cd­(II), and Hg­(II). The specific spatial arrangement of donors within the cavity enabled the formation of complexes featuring short intermetallic distances. Notably, **3-Hg**_**2**_ exhibited an exceptionally short Hg···Hg separation, indicative of a significant mercurophilic interaction. Computational QTAIM analysis revealed bond critical points between the metals, corroborating this interpretation and suggesting that a similar attractive interaction may be operative in the heterobimetallic **3-CdHg**. Accordingly, the present results demonstrate that the confinement within a simple iminopyrrole cage promotes close intermetallic contacts and, for other ions, may facilitate electronic communication between the encapsulated centers.

## Supplementary Material



## Notes

Synthetic procedures and characterization data; Mass spectra, 1D and 2D NMR spectra; Crystallographic data; Bond orders, partial charges, interaction energies, DFT characterization; Cartesian coordinates of all studied systems; CIF file with all SCXRD molecular structures (PDF)

## Notes

Dedicated to Prof. Lechosław Latos-Grażyński on the occasion of his 75th birthday.
