# Supplementary material for: Metallophilicity Under Confinement: The Iminopyrrole Cage Promoting the HgII···MII (M = Hg, Cd) Interactions
Source: Inorg Chem. 2026 Mar 5;65(10):5280–5. doi: 10.1021/acs.inorgchem.6c00290 (PMC13298890; doi:10.1021/acs.inorgchem.6c00290)
Supplement: Supplementary file 1 [file ic6c00290_si_001.pdf]

Supporting Information

Metallophilicity Under Confinement: The Iminopyrrole Cage Promoting the $Hg^{II}\cdots M^{II}$ (M = Hg, Cd) Interactions

by Aleksandra Sarwa,[a] Piotr Krajewski,[b] Bartosz Trzaskowski,[b] Jędrzej P. Perdek,[a] Miłosz Siczek,[a] and Bartosz Szyszko[a]*

[a] Faculty of Chemistry, University of Wrocław, F. Joliot-Curie St. 14, 50-383 Wrocław, Poland; bartosz.szyszko@uwr.edu.pl, www.bszyszko.pl

[b] Centre of New Technologies, University of Warsaw, Banacha 2C, 02-097 Warszawa, Poland

ABSTRACT: Self-assembly of 2,5-diformylpyrrole, tris(2-aminoethyl)amine, and Cd(II) or Hg(II) afforded homobimetallic cryptates. In contrast, the synthesis of heterobimetallic Zn(II)–Cd(II) and Cd(II)–Hg(II) cages required a stepwise approach involving a mono-cadmium(II) precursor. The bis-mercury(II) cryptate exhibited a pronounced mercurophilic interaction, as evidenced by a short Hg···Hg separation of 3.0549(7) Å. Notably, a similarly close intracavity contact was observed for the Cd(II)–Hg(II) system, with a M···M distance of ca. 3.1 Å. The participation of the metal centers in the attractive interaction – enforced by confinement within the iminopyrrole cage – was verified through the DFT calculations of bond indices and was further corroborated by QTAIM analysis.

Table of Contents

Instrumentation ... 3
NMR spectroscopy ... 3
Mass spectrometry ... 3
X-ray diffraction data ... 3
Computational details ... 8
UV-vis spectroscopy ... 11
Synthesis ... 12
Synthesis of 3-Zn_2^{PF6} ... 12
Synthesis of 3-Cd_2^{OAc} ... 12
Synthesis of 3-Hg_2^{OTf} ... 13
Synthesis of 3-Cd^{OAc} ... 14
Synthesis of 3-$CdZn^{PF6}$ ... 14
Synthesis of 3-$CdHg^{PF6}$ ... 15
NMR spectra of 3-Zn_2^{PF6} ... 16
NMR spectra of 3-Cd_2^{OAc} cage ... 18
NMR spectra of 3-Hg_2^{OTf} cage ... 21
NMR spectra of 3-Cd^{OAc} cage ... 25
NMR spectra of 3-$CdZn^{PF6}$ cage ... 28
NMR spectra of 3-$CdHg^{PF6}$ cage ... 32
High-resolution mass spectra ... 36
Mass spectra of 3-Zn_2^{PF6} cage ... 36
Mass spectra of 3-Cd_2^{OAc} cage ... 37
Mass spectra of 3-Hg_2^{OTf} cage ... 38
Mass spectra of 3-Cd^{OAc} cage ... 39
Mass spectra of 3-$CdZn^{PF6}$ cage ... 40
Mass spectra of 3-$CdHg^{PF6}$ cage ... 41
Additional X-ray molecular structures ... 42
Computational results ... 47
UV-vis absorption spectra ... 62
Cartesian Coordinates ... 65
References ... 71

Instrumentation

NMR spectroscopy

The NMR spectra were recorded on high-field spectrometers: a 600.15 MHz instrument equipped with a broadband inverse and observe gradient probe, and a 500.16 MHz instrument equipped with a broadband observe gradient probe. The ^{1}H and ^{13}C NMR spectra were referenced to the residual solvent signal of $CDCl_3$ (^{1}H NMR: δ = 7.24 ppm, ^{13}C NMR: δ = 77.0 ppm), CD_3OD (^{1}H NMR: δ = 3.31 ppm, ^{13}C NMR: δ = 49.0 ppm), or CD_3CN (^{1}H NMR: δ = 1.94 ppm, ^{13}C NMR: δ = 118.26 ppm). The ^{19}F NMR spectra were referenced to hexafluorobenzene C_6F_6 (in CD_3CN ^{19}F NMR: δ = −164.38 ppm).[1]

Mass spectrometry

The mass spectra were recorded on a Bruker qTOF compact spectrometer.

X-ray diffraction data

Single-crystal X-ray diffraction data for all crystals were collected on a Rigaku XtalLAB Synergy R diffractometer equipped with a HyPix-Arc 150 HPAD detector and a rotating anode source (Cu-Kα or Mo-Kα). The diffraction images were processed using CrysAlisPro software.[2] All structures were solved by ShelXT[3] and refined by a ShelXL full matrix least-squares method[4] on F^2 using the Olex2 software suite.[5] All non-hydrogen atoms were refined anisotropically. Carbon-bound hydrogen atoms were constrained based on their corresponding positions using the riding model, unless stated otherwise. Detailed information about disorder treatment is available in the CIF files.

3-Cd: Crystals of **3-Cd**, suitable for SC-XRD analysis, were grown *via* slow evaporation from its solution in acetonitrile. The Gaussian absorption correction from crystal shape was applied. The metallacage was found to be doubly protonated on two of its non-coordinating imine nitrogen atoms. These hydrogen atoms were found on the Fourier difference map and refined without constraints on their atomic displacement parameters (ADPs) and geometry. Next to one iminium group, an acetate anion was located, compensating for the cage's overall +1 charge. The structure was deposited in CCDC with deposition number # 2519799.

3-Cd_2: Crystals of **3-Cd_2**, suitable for SC-XRD analysis, were grown *via* slow evaporation from its solution in acetonitrile. The Gaussian absorption correction from crystal shape was applied. The crystal was at least a 4-component non-merohedral twin; however, the best refinement was achieved by considering only the two major components. Due to the indispensable overlap of reflections from different twin domains, the integration mask size was scaled down by a factor of 0.85. The structure was solved from single-component data derived from twinned data. Refinement was carried out using the HKLF 5 command, with the batch scale factor of 0.8376(8) for the major component. In the crystal cell, two binuclear **[3-Cd_2]$^+$** cationic cages are present, along with one $[Cd_3(OAc)_8]^{2-}$ anion and one acetonitrile molecule, consistent with the crystallization conditions. The central cadmium atom of the linear $[Cd_3(OAc)_8]^{2-}$ cluster was located on an inversion centre. Likewise, the acetonitrile molecule was disordered over an inversion centre; hence, its modelling was facilitated by an appropriate set of 1,2- and 1,3-distance restraints and the rigid bond restraint (RIGU). Additionally, due to the close overlap of acetonitrile's nitrogen atom with the symmetry equivalent of the $-CH_3$ carbon atom, their ADPs were constrained to be the same using the EADP instruction. The structure was deposited in CCDC with deposition number # 2519800.
Alert B: Large Reported Max. (Positive) Residual Density 5.32 eA^{-3}.
Response: The elevated maximum positive residual electron density arises from the crystal being at least a four-component non-merohedral twin. Due to severe reflection overlap, the data could only be reliably integrated and scaled as a two-component twin. The extensive overlap between the remaining twin domains prevented an accurate absorption correction, which, in turn, contributed to the higher residual density in the final difference map.

3-Hg_2: Crystals of **3-Hg_2**, suitable for SC-XRD analysis, were grown *via* slow evaporation from its solution in acetonitrile. The Gaussian absorption correction from crystal shape was applied. The charge of the cationic cage was balanced by the trifluoromethanesulfonate anion, which was disordered over two positions in a ratio of 61:39. To support its modelling, the C–S bond length was restricted to 1.82(2) Å. Consequently, the C–F and S–O bonds were fixed to 1.33(2), and 1.44(2) Å, respectively. The C⋯O 1,3- distances were restricted to 2.57(4) Å, while the F⋯F and O⋯O 1,3- distances were restrained to be the same with a standard deviation of 0.02 Å. Lastly, the rigid bond restraint was applied to all of the triflate's atoms, and the EADP

instruction was implemented to two overlapping disordered oxygen atoms. The structure was deposited in CCDC with deposition number # 2519801.

3-Zn$_2$: Crystals of **3-Zn$_2$**, suitable for SC-XRD analysis, were grown *via* slow diffusion of diethyl ether into solution in acetonitrile. The Gaussian absorption correction from crystal shape was applied. The charge of the cationic cage was balanced by the hexafluorophosphate anion, which was disordered over two positions in a ratio of 58:42. The P–F and 1,3- F–F distances were restrained to be equal with a standard deviation of 0.01 and 0.02 Å, respectively. Additionally, three acetonitrile molecules were found in the crystal structure. They were all disordered, one of them over an inversion centre. An appropriate set of DFIX, DANG, RIGU, and SIMU restraints facilitated their modelling. The structure was deposited in CCDC with deposition number # 2519802.

Note: **3-CdZn** and **3-CdHg** were modelled relying on the mass spectra of the same single crystals, which were used for XRD data collection (see Figure S48 and S51). This revealed the heterometallic nature of the cages, allowing for confident refinement despite disorder.

3-CdZn: Crystals of **3-CdZn**, suitable for SC-XRD analysis, were grown *via* slow diffusion of diisopropyl ether into solution in acetonitrile. The multi-scan absorption correction was applied. Examination of reciprocal space reconstructions indicated non-merohedral twinning, with only *ca.* 58% of reflections indexed to the principal domain. As the twin components could not be reliably separated, the data were reduced as a single crystal, and the twinning was not explicitly accounted for in the refinement. Nevertheless, the integration mask size was scaled down by a factor of 0.8. Refinement included a primary extinction correction (EXTI). In the cage's cavity, Zn and Cd atoms were identified, which were involved in substitutional disorder. Due to a significant difference in the preferred coordination mode of these metals, each metal occupied a distinct position, which facilitated modelling of the disorder. In one pole of the cage's cavity, there was a Cd1 atom with a site occupation factor (sof) of 0.6 and a Zn2 atom (*sof* = 0.4). Consequently, the other pole featured Zn1 (*sof* = 0.6) and Cd2 (*sof* = 0.4). The positive charge of the cage was equalized by a hexafluorophosphate anion, whose equatorial fluorine atoms were disordered over two positions. Their modelling was supported by SADI, RIGU, and SIMU instructions. An acetonitrile

molecule was located on the difference Fourier map. The structure was deposited in CCDC with deposition number # 2519803.

3-CdHg: Crystals of **3-CdHg**, suitable for SC-XRD analysis, were grown *via* slow diffusion of diisopropyl ether into solution in acetonitrile. The analytical absorption correction from crystal shape was applied. In the cavity of the cage Cd and Hg atoms were located, which were disordered so that one metal centre consisted of Cd1 (*sof* = 0.77) and Hg1A (*sof* = 0.23), and the other comprised Hg1 (*sof* = 0.77) and Cd1A (*sof* = 0.23). The charge of the cationic cage was compensated by hexafluorophosphate anion. The structure was deposited in CCDC with deposition number # 2519804.

Table S1. Selected X-ray diffraction data.

	CCDC 2519799	CCDC 2519800	CCDC 2519801	CCDC 2519802	CCDC 2519803	CCDC 2519804
**structure name**	3-Cd	3-Cd_2	3-Hg_2	3-Zn_2	3-CdZn	3-CdHg
**space group**	$P2_1/c$	$P2_1/c$	$P2_1/c$	$P2_1/c$	P-1	$P2_1/c$
**crystal system**	monoclinic	monoclinic	monoclinic	monoclinic	triclinic	monoclinic
**empirical formula**	$C_{36}H_{47}N_{13}O_2Cd$	$C_{78}H_{99}N_{23}O_{16}Cd_7$	$C_{31}H_{36}F_3N_{11}O_3SHg_2$	$C_{33.90}H_{41.85}F_6N_{12.95}PZn_2$	$C_{32}H_{39}CdF_6N_{12}PZn$	$C_{30}H_{36}CdF_6HgN_{11}P$
**formula weight**	806.26	2401.60	1100.95	906.46	914.49	1008.66
**temperature [K]**	100.00(10)	101(2)	230.0(6)	100.00(10)	99.97(10)	100.00(10)
**a**	14.238(3)	14.274(3)	12.656(3)	13.490(3)	11.483(3)	13.308(3)
**b**	13.130(3)	21.257(5)	18.932(5)	23.030(5)	13.097(3)	18.036(4)
**c**	20.332(4)	14.420(3)	15.541(4)	14.044(3)	13.448(3)	15.333(3)
**α**	–	–	–	–	66.60(2)	–
**β**	100.41(2)	93.88(2)	110.76(2)	117.48(2)	79.52(3)	113.94(5)
**γ**	–	–	–	–	74.03(3)	–
**V**	3738.4(14)	4365.3(17)	3481.9(16)	3870.8(16)	1778.5(8)	3363.7(17)
**Z**	4	2	4	4	2	4
**μ [mm^{-1}]**	5.096	14.039	16.737	2.559	6.706	5.313
**F(000)**	1672	2384	2104	1860	924	1960
**2Θ range for data collection [°]**	3.156 to 73.232	3.103 to 74.158	3.735 to 73.074	3.693 to 74.584	3.593 to 76.119	2.019 to 44.575
**radiation type**	Cu K\α	Cu K\α	Cu K\α	Cu K\α	Cu K\α	Mo K\α
**index ranges**	-17 ≤ h ≤ 16; -15 ≤ k ≤ 12; -23 ≤ l ≤ 24	-17 ≤ h ≤ 17; -26 ≤ k ≤ 26; -16 ≤ l ≤ 17	-15 ≤ h ≤ 15; -19 ≤ k ≤ 23; -18 ≤ l ≤ 19	-16 ≤ h ≤ 16; -28 ≤ k ≤ 28; -17 ≤ l ≤ 15	-14 ≤ h ≤ 14; -16 ≤ k ≤ 16; -16 ≤ l ≤ 16	-22 ≤ h ≤ 20; -30 ≤ k ≤ 35; -30 ≤ l ≤ 25
**reflections collected**	27377	17243	25632	41570	31521	82796
**independent reflections**	7266	17243	6788	7769	7271	21482
**reflections observed I>=2σ(I)**	6525	13276	5967	7060	7127	12205
**completeness**	0.999	0.998	0.999	1.000	0.998	0.999
**R int**	0.0337	–	0.0242	0.0238	0.0294	0.0298
**Final R indexes [I>=2σ(I)]**	R_1=0.0303, wR_2=0.0789	R_1=0.0753, wR_2=0.2165	R_1=0.0325, wR_2=0.0846	R_1=0.0379, wR_2=0.1012	R_1=0.0804, wR_2=0.2040	R_1=0.0494, wR_2=0.1026
**Final R indexes (all data)**	R_1=0.0343, wR_2=0.0810	R_1=0.0918, wR_2=0.2333	R_1=0.0379, wR_2=0.0873	R_1=0.0419, wR_2=0.1035	R_1=0.0810, wR_2=0.2043	R_1=0.1140, wR_2=0.1182
**goodness of fit on F^2**	1.066	1.061	1.045	1.070	1.152	1.032
**Δρ min, Δρ max [eÅ^{-3}]**	-0.975, 0.552	-1.666, 5.318	-1.999, 1.224	-0.622, 0.758	-2.018, 2.307	-0.889, 1.885

Computational details

The calculations were performed using Orca 6.1.0 software[6] and xTB ver. 6.4.1 program[7]. The geometries of all starting structures were optimized at both the GFN2-xTB[8] and DFT levels of theory. For semiempirical tight-binding calculations, an analytical linearized Poisson-Boltzmann model of methanol was implemented. DFT calculations were performed at the ωB97X-D level of theory with the latest D4 dispersion correction,[9–11] which, based on benchmark results from the GMTKN55 and TMC32 databases, is among the best-performing and most computationally efficient approaches for the energetics and geometry optimization of transition-metal complexes.[12,13] Atoms were described with the Ahlrichs def2 basis set family[14] with def2-ECP pseudopotentials for Cd and Hg. The RIJCOSX approximation with the def2/J auxiliary basis set was utilized as the default for hybrid functionals in Orca software. DFT results presented in the main part of the manuscript are for systems with no solvent model; however, we also used the SMD implicit solvation model of acetonitrile for comparison. Conformational and tautomeric searches were performed on molecules optimized at the GFN2-xTB level of theory using the Conformer-Rotamer Ensemble Sampling Tool (CREST) ver. 2.1 program.[15,16] Metal-ligand and metal-metal bond indices were evaluated for DFT optimized structures using Multiwfn ver. 3.8 program.[17,18] VMD ver. 1.9.3 software was used to prepare the figures.[19]

Crystallographic structures of the synthesized homobimetallic cages were shown to feature each metal center being coordinated by three imine sites, one amine group, and three pyrrolide anions acting as bridging ligands. In **3-Zn$_2$**, the metal–ligand bond lengths at both metal centers were found to be fairly similar across the corresponding coordinating groups. In contrast, in the **3-Cd$_2$** and **3-Hg$_2$** cages, more asymmetric ligand arrangements around the two metal centers were observed. In the case of **3-Hg$_2$**, this desymmetrization was evident, with two pyrrolides displaced toward one metal center and the third toward the other. To determine whether this effect was caused by the interaction of the counterion with one of the metal centers, [**3-Hg$_2$**]$^+$ was studied in both the presence and absence of its triflate counterion, while the remaining cages were examined without counterions.

In the initial studies, the geometries of the cryptates were optimized at the GFN2-xTB level of theory, and the resulting structures were subjected to a conformational search using CREST. An energy cutoff of 30 kcal/mol was applied, along with the root mean

square deviation (RMSD) and energy difference thresholds of 1 Å and 1 kcal/mol, respectively, to eliminate redundant conformers with nearly identical geometries. Three conformers were identified for **3-Zn_2** and **3-Cd_2** cages and four for **3-Hg_2**, with energy differences relative to the lowest-energy conformer ranging from 5.6 kcal/mol to 14.6 kcal/mol (Table S2). With increasing ionic radius of the metal, a slight expansion of the coordination sphere was observed, leading to changes in bond lengths and angles within the cage. As a consequence, an increase in cage flexibility was inferred, and the relative conformer energies were found to increase with the atomic mass of the coordinated metal ions. Nevertheless, at room temperature, such energy separations correspond to very low Boltzmann populations, indicating that the higher-energy conformers are unlikely to be significantly populated and that the cages can be regarded as conformationally rigid.

To further analyze cage flexibility, molecular dynamics simulations were performed. The simulations were carried out at 300 K using a 1 fs time step for propagation. The total simulation time was set to 100 ps, corresponding to 100000 integration steps, with atomic coordinates being saved every 100 fs. Temperature control was achieved using a Nosé–Hoover chain thermostat at 300 K with a coupling strength of 20 fs. Throughout the simulations, for all cages, the metal ions were found to remain bound within the cage, while the pyrrolide bridging ligands were observed to alternate their degree of coordination between the two metal centers. Additionally, shifts in the positions of the metal centers and slight changes in the metal–metal distances were detected.

The cage geometries derived from the crystallographic structures were further optimized at the DFT level of theory. To identify a basis set that provides an appropriate balance between computational cost and accuracy, calculations were performed using the def2-SVP, def2-TZVP, def2-TZVPP, and def2-QZVP basis sets. Expansion of the basis set beyond TZVP was found to have only a minor effect on the molecular geometries, as evidenced by small changes in the RMSD between the crystallographic and optimized structures (Table S3). The DFT-optimized structures were determined to be essentially symmetric, as inferred from the lengths of selected ligand–metal bond pairs. The RMSD values for the **3-Zn_2** and **3-Cd_2** cages indicate that the chosen functional, in combination with the def2-TZVP basis set, yields geometries consistent with the experimental data. In contrast, a higher RMSD value was obtained for **3-Hg_2**, which can be attributed to desymmetrization of the crystallized cage resulting from

ligand shifts toward one of the metal ions. Optimization of **3-Hg$_2$** geometry in the presence of the triflate counterion resulted in significantly improved agreement with the crystallographic structure. This optimized structure was characterized by a shift of one pyrrolide bridging ligand toward one of the Hg ions, presumably driven by electrostatic interactions between the triflate counterion and the adjacent Hg center. This behavior is reflected in the ligand–metal bond lengths and in the lower RMSD value obtained for the cage alone, excluding the anion. Additionally, the geometries of the lowest-energy conformers obtained from the CREST conformational search were optimized at the DFT level of theory using the def2-TZVP basis set. The RMSD values between the DFT-optimized geometries derived from the crystallographic and CREST structures were below 0.02 Å for all three cages, further confirming the high accuracy of the CREST conformational search procedure.

The same analysis was carried out for the heterometallic **3-CdZn** and **3-CdHg** cages. For **3-CdZn**, similarly to the **3-Hg$_2$** cage, the pyrrolide ligands were shifted toward one of the metal ions, in this case, the cadmium center. To compensate for this effect, the coordination sphere of the Zn cation was stabilized by significantly stronger coordination of the tertiary amine nitrogen. To verify whether such a geometry could arise from interactions with counterions and/or solvent molecules present in the crystallographic structure, calculations were performed in the presence of a BF_4^- anion and an acetonitrile molecule, as well as exclusively with the anion. The starting geometry of **3-CdHg** was constructed based on the **3-CdZn** cage structure.

For the heterobimetallic cage structures optimized at the GFN2-xTB level of theory, a conformational search was conducted using CREST. For **3-CdZn**, three conformers were identified, whereas for **3-CdHg**, the presence of a metal ion with a larger ionic radius was found to expand the cage cavity, increasing flexibility around the coordination centers, and leading to the formation of four conformers. In all conformers, similarly to the homometallic cages, a distinct shift of two pyrrolide ligands toward individual metal ions was observed, while the third pyrrolide anion was shared between both metal centers. The energy differences relative to the lowest-energy conformer were determined to range from 5.9 to 14.8 kcal/mol, indicating that the higher-energy conformers are expected to be only sparsely populated at room temperature.

The most significant difference between the conformers was identified as the extent of involvement of the tertiary amine groups in the metal coordination. In **3-CdZn**, the lowest-energy conformer featured amine groups located relatively far from the metal ions, whereas at higher conformer energies the amine groups approached the metal centers, accompanied by a slight elongation of the metal–metal distance. This behavior indicates that the bending of the rigid amine fragment is energetically unfavorable. In the **3-CdHg** cage, a similar shift of the amine groups was observed; however, no systematic elongation of the metal–metal distance was detected with increasing conformer energy, although the lowest-energy conformer exhibited the shortest metal–metal separation.

Molecular dynamics simulations of **3-CdZn** and **3-CdHg** cages were performed and found to proceed in a manner analogous to that observed for the previously analyzed homometallic cages. Throughout the simulations, the metal ions remained confined within the cages, while the pyrrolide ligands alternated their coordination between the two metal centers. Small shifts in the positions of the metal ions and minor variations in the metal–metal distances were also detected.

The geometries of **3-CdZn** derived from the crystallographic structure, as well as those of the CREST-generated conformers, were optimized at the DFT level of theory using the def2-TZVP basis set. The RMSD value between the DFT-optimized crystallographic structure and the lowest-energy conformer was determined to be 0.03 Å, confirming that the most stable conformer is formed in the solid state. The RMSD between the DFT-optimized geometry and the crystallographic structure was 0.55 Å. Optimization of the crystallographic structure in the presence of the counterion, and subsequently with inclusion of a solvent molecule, led to only marginal improvement in agreement between the experimental and calculated geometries (RMSD values of 0.53 and 0.51 Å, respectively), suggesting that desymmetrization of the crystallographically-determined structure of the cage is induced primarily by interactions with neighboring cages in the solid state.

UV-vis spectroscopy

Electronic spectra were recorded on a Varian Cary-60 Bio spectrophotometer equipped with a Peltier unit. The spectra were collected using a quartz cuvette at 25°C.

Synthesis

2,5-Diformylpyrrole **1** was synthesized as described in the literature.[20]

Chloroform for synthesis, and chloroform-*d* was freshly prepared prior to use by passing it through a basic alumina column. All other reagents and solvents not mentioned were used without further purification.

Synthesis of 3-Zn_2^{PF6}

3-Zn_2^{OAc} was obtained as described in the literature.[21] **3-Zn_2^{OAc}** (6.4 mg, 0.0086 mmol) was placed in a glass vial and dissolved in 10 mL of MeOH. To the stirred solution, an excess of $TBAPF_6$ (80 mg, 0.21 mmol) was added to precipitate **3-Zn_2PF_6**. The suspension was then stirred for 5 minutes, then transferred to a polypropylene tube and centrifuged. The precipitate was washed 2 × 5 mL with MeOH and dried in vacuo for 2 hours to afford **3-Zn_2^{PF6}** as a brown solid. Yield: 95% (6.8 mg).

^{1}H NMR (CD_3CN, 600 MHz, 300 K) δ (ppm): 8.08 (s, 6H, im), 6.65 (s, 6H, β-pyrr), 3.05–2.95 (m, 18H, CH_2), 2.62–2.50 (m, 6H, CH_2).

^{13}C NMR (CD_3CN, 126 MHz, 300 K) δ (ppm): 161.5, 142.5, 119.5, 58.5, 55.6.

^{19}F NMR (CD_3CN, 471 MHz, 300 K) δ (ppm): −72.9 (d, $^1J_{P,F}$ = 710 Hz, PF_6^-).

HR-MS (ESI+, TOF): *m/z* 682.2196 calcd. for $[C_{30}H_{36}N_{11}Zn_2]^+$ 682.1691.

Synthesis of 3-Cd_2^{OAc}

Diformylpyrrole **1** (123 mg, 1 mmol, 3 equiv.) and $Cd(OAc)_2 \cdot 2H_2O$ (360 mg, 1.3 mmol, 4 equiv.) were placed in a 25 mL round-bottom flask, and *n*-butanol (50 mL) was added. After 5 minutes of stirring, DIPEA (260 µL, 1.5 mmol, 4.5 equiv.) was added *via* a Hamilton syringe, followed by tris(2-aminoethylamine) **2** (100 µL, 0.034 mmol, 2 equiv.). The mixture was refluxed for 20 hours. After that time, the reaction mixture was evaporated to dryness using a rotary evaporator. Next, the solid was dissolved in ca. 10 mL of MeOH, and an excess of diethyl ether was added to precipitate **3-Cd_2^{OAc}**. The solution was then removed, and the procedure was repeated twice. The remaining solid was washed with diethyl ether and dried *in vacuo* for 2 hours to afford **3-Cd_2^{OAc}** as a brown solid. Yield: 72% (200 mg). *Due to the inability to remove the excess $Cd(OAc)_2$, the yield was quantified by NMR using an external standard (C_6H_6).*

^{1}**H NMR** (CD_3CN, 600 MHz, 300 K) δ (ppm): 8.18 (s, $^3J_{Cd,H}$ = 49 Hz, 6H, im), 6.71 (s, 6H, β-pyrr), 3.29–3.22 (m, 6H, CH_2), 3.20–3.11 (m, 6H, CH_2), 2.92 (dd, *J* = 13.6 Hz, 3.0 Hz, CH_2), 2.62 (td, *J* = 13.2 Hz, 4.0 Hz, CH_2), 1.89 (b, CH_3COO). *Due to an excess of $Cd(OAc)_2$ in the sample, the integration of the C**H_3**COO⁻ signal couldn't be precisely determined.*

^{13}**C NMR** (CD_3CN, 151 MHz, 300 K) *δ* (ppm): 161.9, 142.9, 121.4, 59.0, 55.6, 21.6. *One signal from the counterion (acetate, CH_3**C**OO⁻) couldn't be detected.*

HR-MS (ESI+, TOF): *m/z* 776.1240 calcd. for $[C_{30}H_{36}N_{11}Cd_2]^+$ 776.1225.

Synthesis of 3-Hg_2^{OTf}

Diformylpyrrole **1** (12.4 mg, 0.10 mmol, 3 equiv.) was placed in a 25 mL round-bottom flask and dissolved in $CHCl_3$ (10 mL). Next, $Hg(OTf)_2$ (51 mg, 0.10 mmol, 3 equiv.) was added. After stirring for 5 minutes, DIPEA (26 µL, 0.15 mmol, 4.5 equiv.) was added *via* a Hamilton syringe, followed by tris(2-aminoethylamine) **2** (10 µL, 0.067 mmol, 2 equiv.). The mixture was stirred for 20 hours at reflux under a reflux condenser. The crude reaction mixture was then transferred to a polypropylene tube and centrifuged. The solution was then removed, and the solid was washed with 10 mL of $CHCl_3$. Next, the remaining solid was extracted with 3 × 5 mL of acetonitrile. The MeCN extracts were combined and concentrated to ca. 5 mL, after which diethyl ether was added to precipitate the product. The solid was then washed with 5 mL diethyl ether and 5 mL of MeOH and dried under vacuum for 2 hours to afford **3-Hg_2^{OTf}** as an orange solid. Yield: 26% (9.6 mg).

^{1}**H NMR** (CD_3CN, 600 MHz, 300 K) δ (ppm): 8.34 (s, $^3J_{Hg,H}$ = 161 Hz, 6H, im), 6.75 (t, *J* = 4.6 Hz, 6H, β-pyrr), 3.37 (td, *J* = 12.1 Hz, 3.3 Hz, 6H, CH_2), 3.27 (dd, *J* = 12.1 Hz, 3.6 Hz, 6H, CH_2), 2.95 (dd, *J* = 13.5 Hz, 2.9 Hz, 6H, CH_2), 2.71 (td, *J* = 13.3 Hz, 3.9 Hz, 6H, CH_2).

^{13}**C NMR** (CD_3CN, 151 MHz, 300 K) δ (ppm): 160.9, 141.2, 121.8, 58.5, 57.2.

^{19}**F NMR** (CD_3CN, 471 MHz, 300 K) δ (ppm): −79.3 (s, OTf⁻).

HR-MS (ESI+, TOF): *m/z* 952.2592 calcd. for $[C_{30}H_{36}N_{11}Hg_2]^+$ 952.2621.

Synthesis of 3-Cd^{OAc}

Diformylpyrrole **1** (12.4 mg, 0.10 mmol, 3 equiv.) and $Cd(OAc)_2·2H_2O$ (8.8 mg, 0.33 mmol, 1 equiv.) were placed in a 25 mL round-bottom flask and dissolved in *n*-butanol (10 mL). After complete dissolution of reagents, DIPEA (26 µL, 0.15 mmol, 4.5 equiv.) was added *via* a Hamilton syringe, followed by tris(2-aminoethylamine) **2** (10 µL, 0.067 mmol, 2 equiv.). The mixture was stirred for 20 hours at 25 ºC. Next, the solution was evaporated to dryness, then dissolved in 10 mL of MeOH and filtered. The methanolic solution was evaporated and dried in vacuo for 2 hours to afford **3-Cd^{OAc}** as yellow solid. Yield: 96% (23.1 mg).

^{1}H NMR ($CDCl_3$, 500 MHz, 300 K) δ (ppm): 8.23 (d, ^4J = 1.7 Hz, $^3J_{Cd,H}$ = 32 Hz, 1H, im^{Cd}), 8.21 (^4J = 1.7 Hz, $^3J_{Cd,H}$ = 32 Hz, 1H, im^{Cd}), 8.10 (d, ^4J = 1.2 Hz, $^3J_{Cd,H}$ = 32 Hz, 1H, im^{Cd}), 7.40 (m, 1H, β-pyrr), 7.23 (b, 1H, im), 7.07 (m, 1H, β-pyrr), 6.94 (d, ^3J = 3.9 Hz, 1H, β-pyrr), 6.75 – 6.70 (m, 2H, β-pyrr), 6.58 (m, 1H, β-pyrr), 6.29 (b, 1H, im), 6.13 (b, 1H, im), 4.16 (t, J = 11.6 Hz, 1H, CH_2), 3.91 (t, J = 12.6 Hz, 2H, CH_2), 3.81 (t, J = 11.9 Hz, 1H, CH_2), 3.63 (d, J = 12.1 Hz, 1H, CH_2), 3.42–3.18 (m, 8H, CH_2), 3.06 (d, J = 13.8 Hz, 1H, CH_2), 2.99–2.88 (m, 6H, CH_2), 2.85–2.67 (m, 4H, CH_2), 1.99 (s, 3H, CH_3COO^-).

^{13}C NMR ($CDCl_3$, 151 MHz, 300 K) δ (ppm): 177.5, 160.6, 159.6, 159.1, 157.5, 157.1, 156.8, 145.8, 144.6, 140.9, 140.7, 135.5, 132.6, 121.5, 121.2, 119.8, 119.1, 118.1, 113.7, 64.5, 62.5, 62.0, 61.9, 57.0, 56.9, 56.2, 55.5, 55.0, 54.8, 54.6, 49.0, 23.9.

HR-MS (ESI+, TOF): *m/z* 666.2320 calcd. for $[C_{30}H_{36}N_{11}Cd+2H]^+$ 666.2347.

Synthesis of 3-CdZn^{PF6}

3-Cd^{OAc} (23 mg, 0.032 mmol, 1 equiv.) was placed in a glass vial and dissolved in 10 mL of methanol. To the stirred solution, $Zn(OAc)_2$ (10.5 mg, 0.048 mmol, 1.5 equiv.) was added in one portion. The mixture was stirred for 5 minutes before an excess of $TBAPF_6$ (37 mg, 0.095 mmol, 3 equiv.) was added to precipitate the product. The suspension was then transferred to a polypropylene tube and centrifuged. The methanolic solution was removed, and the solid was washed 2 × 5 mL of methanol. The residue was dried in vacuo for 2 hours to afford **3-CdZn^{PF6}** as a yellow solid. Yield: 15.2 mg (55 %). *Prolonged stirring with an excess of $Zn(OAc)_2$ (prior to the addition of $TBAPF_6$) resulted in the formation of a mixture of **3-CdZn^{OAc}**, **3-Zn$_2^{OAc}$**, and **3-Cd$_2^{OAc}$**, with the homobimetallic cages being the major products.*

^{1}H NMR (CD_3CN, 600 MHz, 300 K) δ (ppm): 8.15 (d, ^{4}J = 1.0 Hz, $^{3}J_{Cd,H}$ = 43 Hz, 3H, im^{Cd}), 8.13 (s, 3H, im^{Zn}), 6.73 (m, 3H, β-pyrr), 6.65 (m, 3H, β-pyrr), 3.22–3.00 (m, 12H, CH_2), 2.90 (dd, J = 13.6 Hz, 3.3 Hz, 4H, CH_2), 2.65–2.52 (m, 8H, CH_2).

^{13}C NMR (CD_3CN, 151 MHz, 300 K) δ (ppm): 162.9, 160.8, 144.1, 141.4, 121.5, 119.8, 59.0, 58.7, 55.7, 55.6.

^{19}F NMR (CD_3CN, 471 MHz, 300 K) *δ* (ppm): −72.8 (d, $^{1}J_{F,P}$ = 710 Hz, PF_6^-).

HR-MS (ESI+, TOF): *m/z* 728.1441 calcd. for $[C_{30}H_{36}N_{11}CdZn]^+$ 728.1467.

Synthesis of 3-CdHg^{PF6}

3-Cd^{OAc} (23 mg, 0.032 mmol, 1 equiv.) was placed in a glass vial and dissolved in 10 mL of methanol. To the stirred solution, $Hg(OAc)_2$ (12.2 mg, 0.038 mmol, 1.2 equiv.) was added in one portion. The mixture was stirred for 5 minutes before an excess of $TBAPF_6$ (38 mg, 0.095 mmol, 3 equiv.) was added to precipitate the product. The suspension was then transferred to a polypropylene tube and centrifuged. The methanolic solution was removed, and the solid was washed 2 × 5 mL of methanol. The residue was dried in vacuo for 2 hours to afford **3-CdHg^{PF6}** as a yellow solid. Yield: 19 mg (59 %). *Prolonged stirring with an excess of $Hg(OAc)_2$ (prior to the addition of $TBAPF_6$) resulted in a mixture of **3-CdHg^{OAc}**, **3-Cd$_2^{OAc}$**, and **3-Hg$_2^{OAc}$**, with the homobimetallic cages being the major products.*

^{1}H NMR (CD_3CN, 600 MHz, 300 K) δ (ppm): 8.30 (b, $^{3}J_{Hg,H}$ = 170 Hz, 3H, im^{Hg}), 8.22 (b, $^{3}J_{Cd,H}$ = 45 Hz, im^{Cd}), 6.75 (m, 3H, β-pyrr), 6.71 (m, 3H, β-pyrr), 3.44–3.36 (m, 3H, CH_2), 3.34–3.24 (m, 6H, CH_2), 3.18–3.08 (m, 3H, CH_2), 3.00–2.89 (m, 6H, CH_2), 2.74 (td, $J_{H,H}$ = 13.0 Hz, 3.8 Hz, 3H, CH_2), 2.63 (td, $J_{H,H}$ = 13.2 Hz, 3.9 Hz, 3H, CH_2).

^{13}C NMR (CD_3CN, 151 MHz, 300 K) δ (ppm): 161.7, 161.2, 143.0, 140.9, 122.6, 120.8, 59.3, 57.9, 56.7, 56.1.

^{19}F NMR (CD_3CN, 471 MHz, 300 K) *δ* (ppm): −72.8 (d, $^{1}J_{F,P}$ = 710 Hz, PF_6^-).

HR-MS (ESI+, TOF): 864.1878 calcd. for $[C_{30}H_{36}N_{11}CdHg]^+$ 864.1888.

NMR spectra of 3-Zn_2^{PF6}

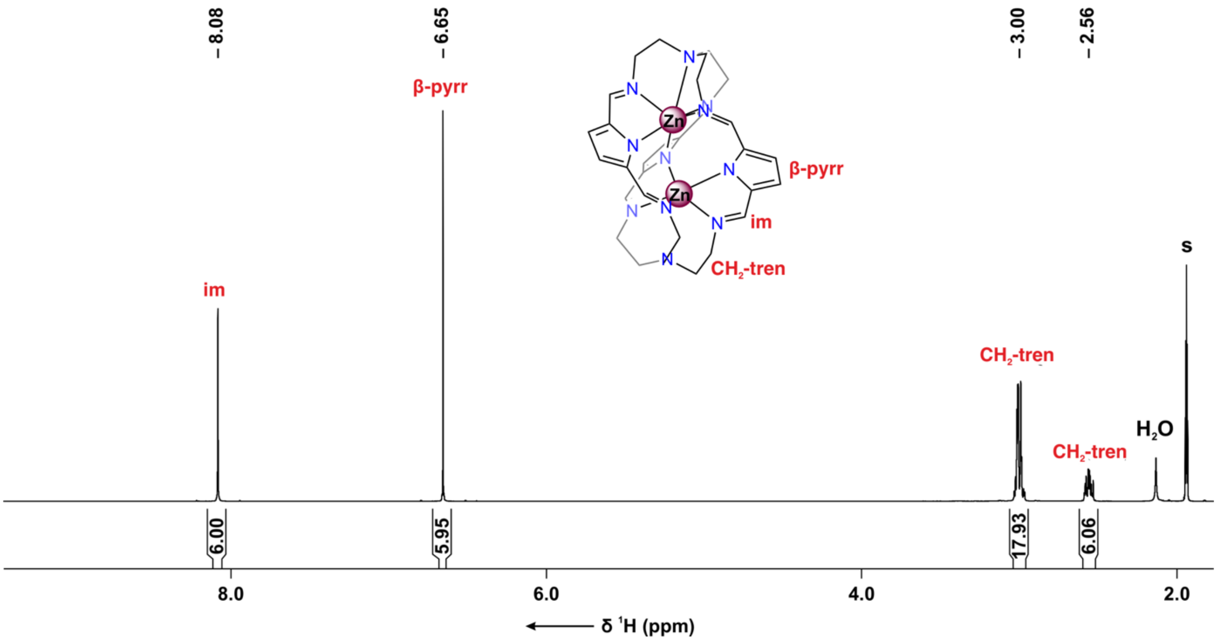

Figure S1. The ^{1}H NMR spectrum of **3-Zn_2^{PF6}** (600 MHz, CD_3CN, 300 K).

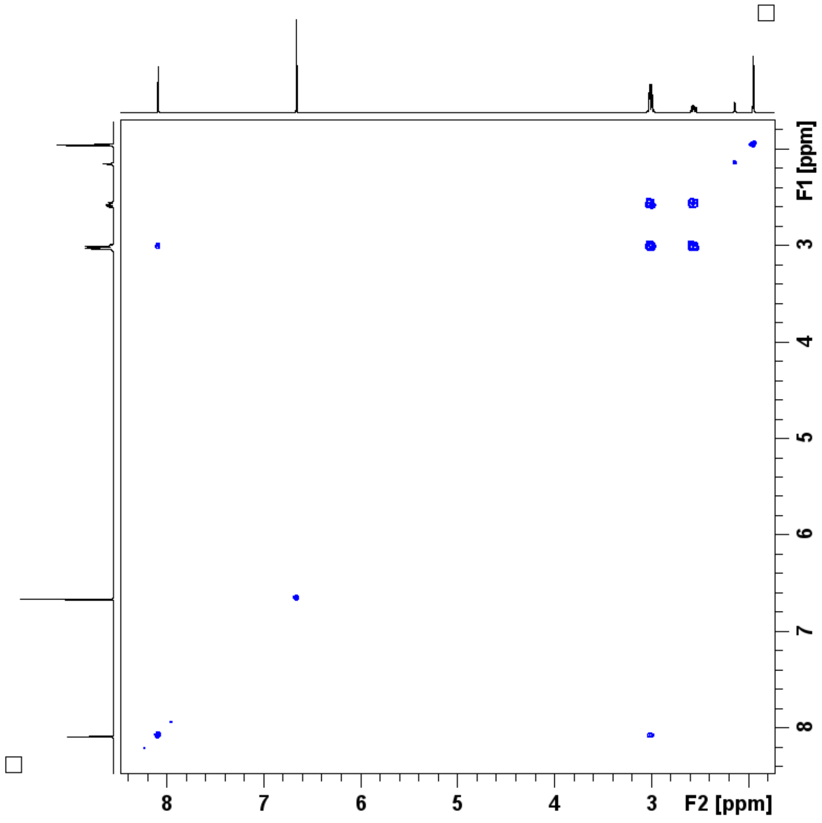

Figure S2. The ^{1}H-^{1}H COSY spectrum of **3-Zn_2^{PF6}** (600 MHz, CD_3CN, 300 K).

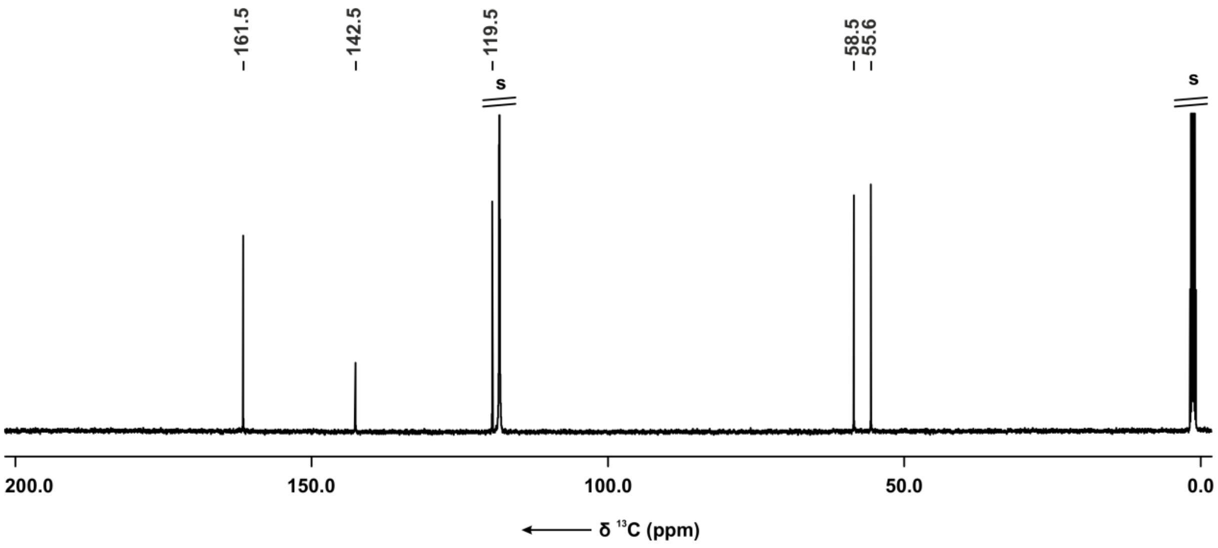

Figure S3. The ^{13}C NMR spectrum of **3-Zn$_2$^{PF6}** (126 MHz, CD_3CN, 300 K).

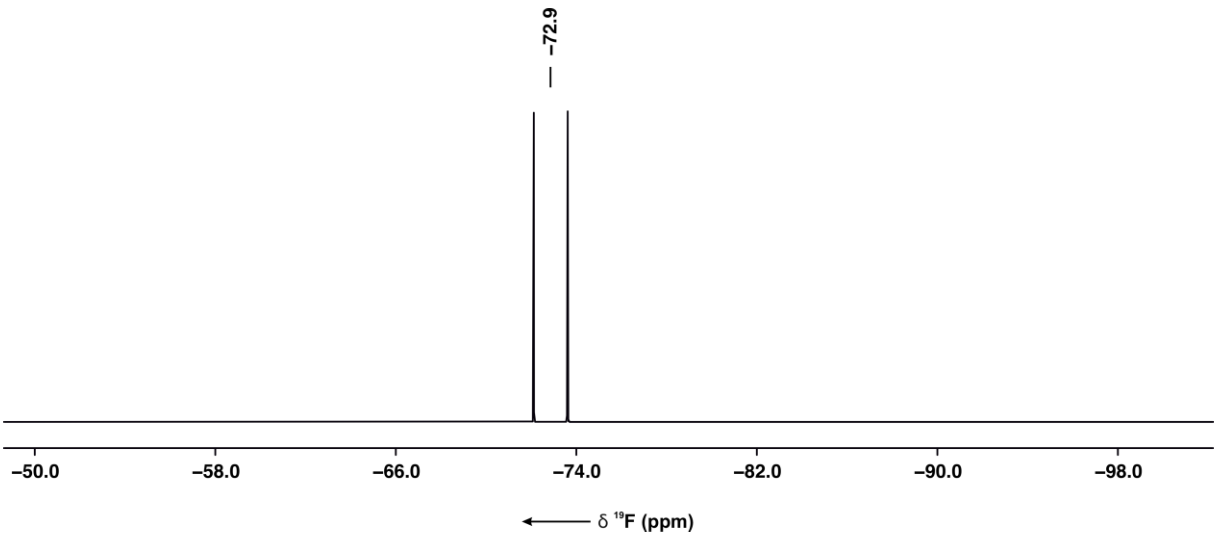

Figure S4. The ^{19}F NMR spectrum of **3-Zn$_2$^{PF6}** (471 MHz, CD_3CN, 300 K).

NMR spectra of 3-Cd_2^{OAc} cage

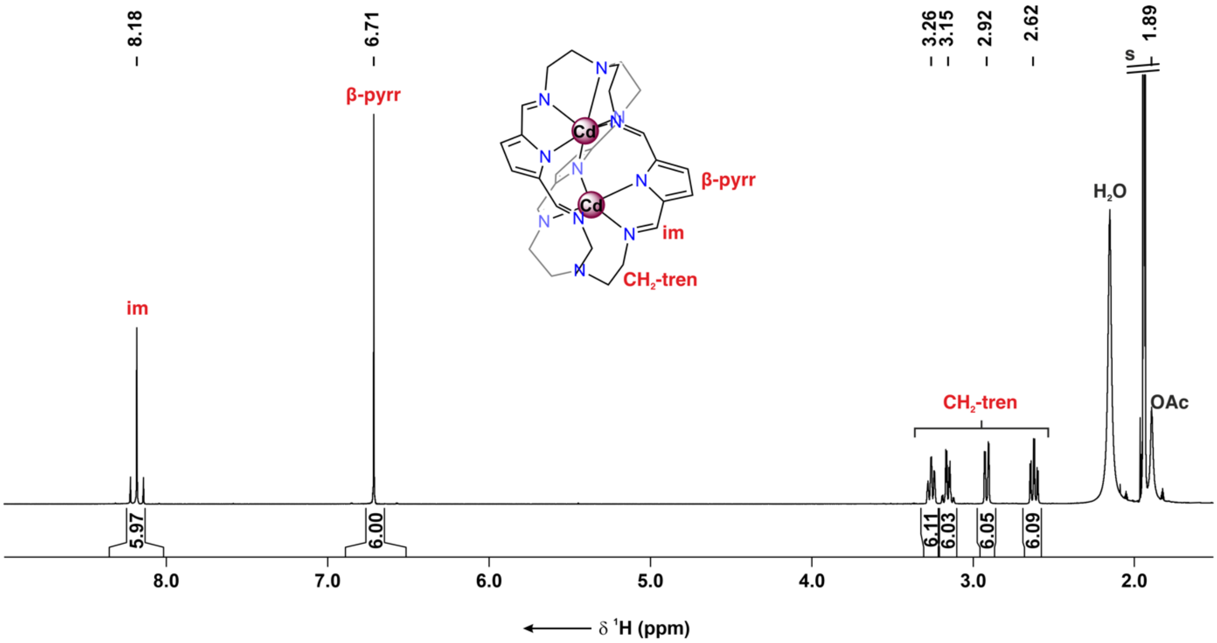

Figure S5. The ^{1}H NMR spectrum of **3-Cd_2^{OAc}** (600 MHz, CD_3CN, 300 K).

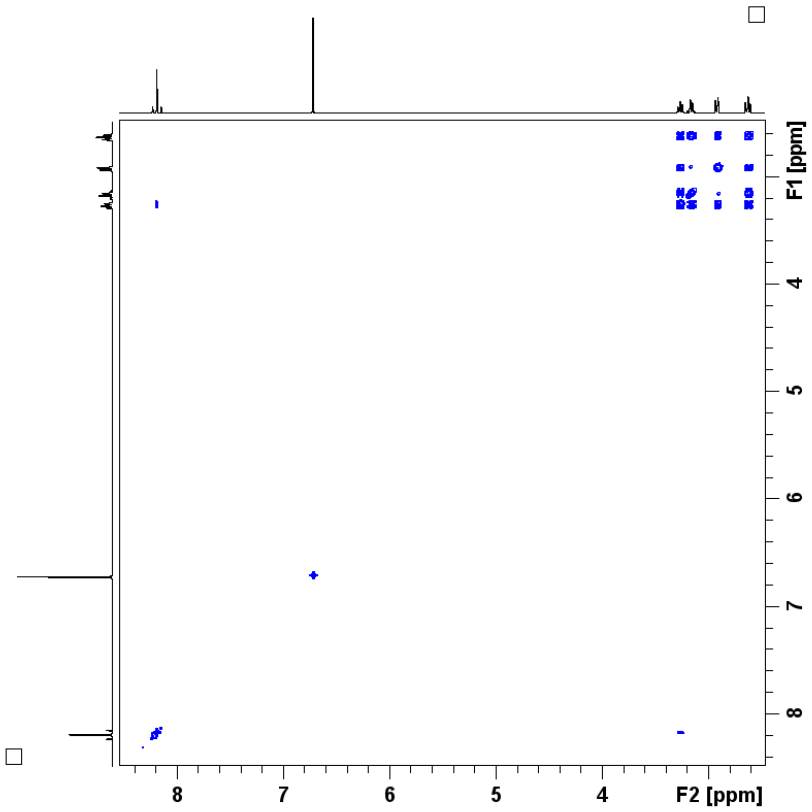

Figure S6. The ^{1}H-^{1}H COSY spectrum of **3-Cd_2^{OAc}** (600 MHz, CD_3CN, 300 K).

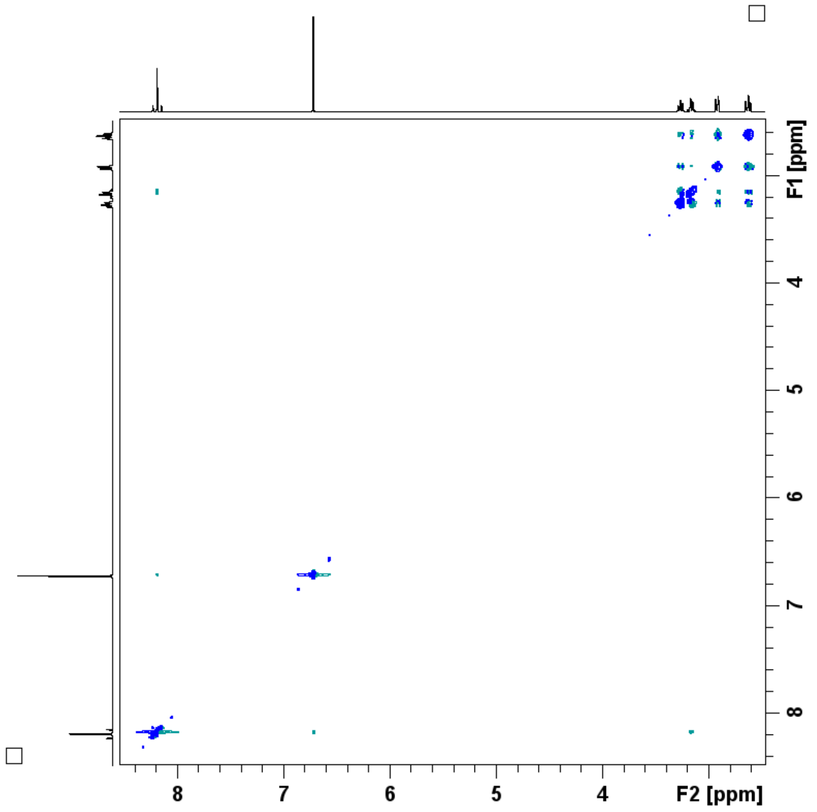

Figure S7. The ^{1}H-^{1}H NOESY spectrum of **3-Cd$_2$^{OAc}** (600 MHz, CD_3CN, 300 K).

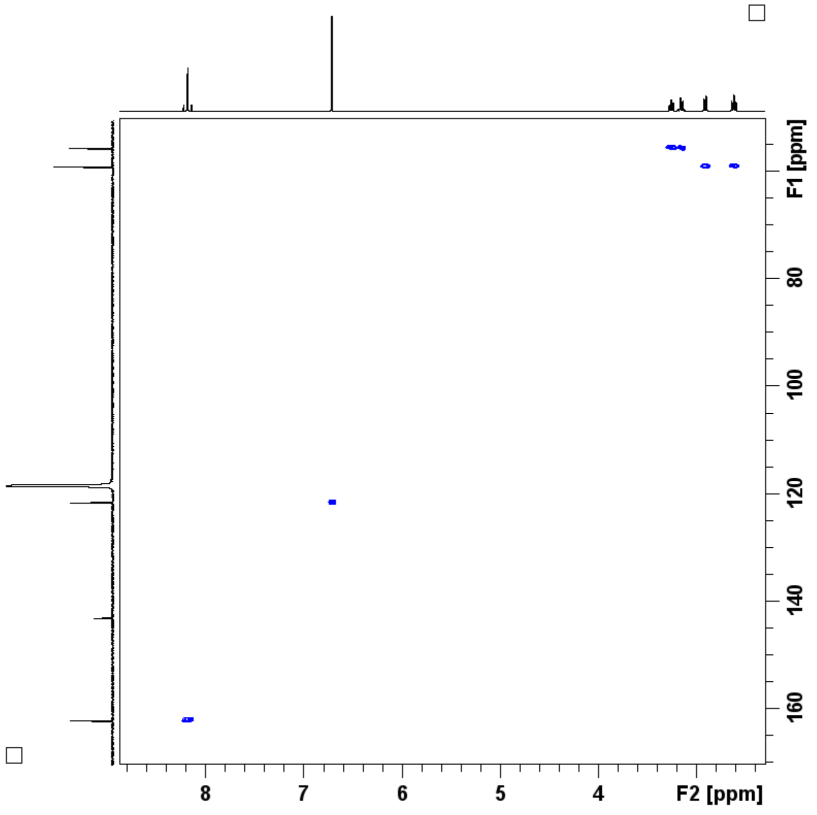

Figure S8. The ^{1}H-^{13}C HMQC spectrum of **3-Cd$_2$^{OAc}** (600 MHz, CD_3CN, 300 K).

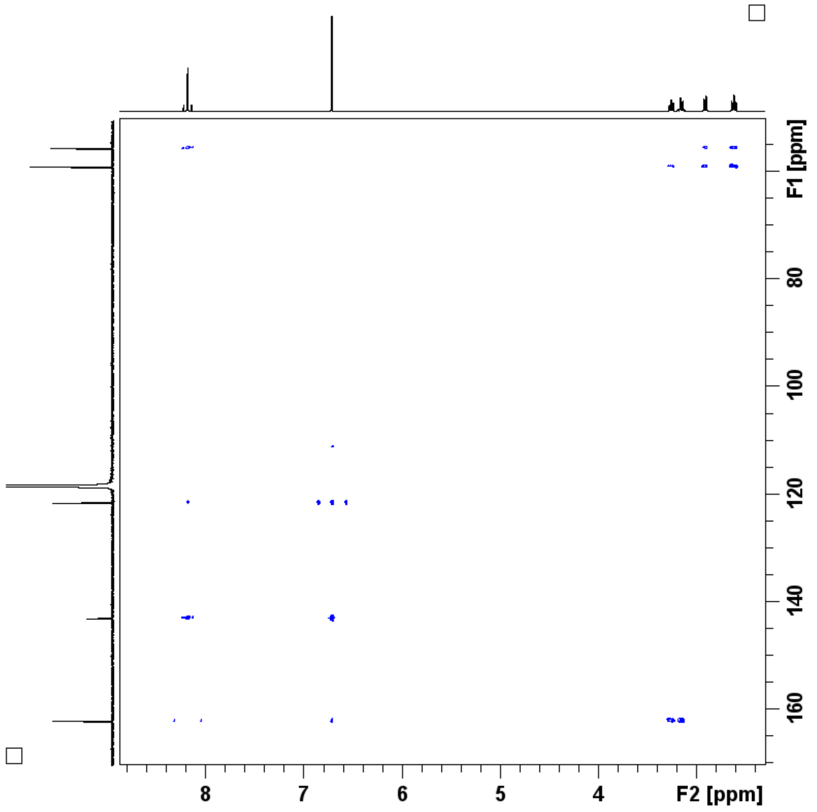

Figure S9. The ^{1}H-^{13}C HMBC spectrum of **3-Cd$_2^{OAc}$** (600 MHz, CD_3CN, 300 K).

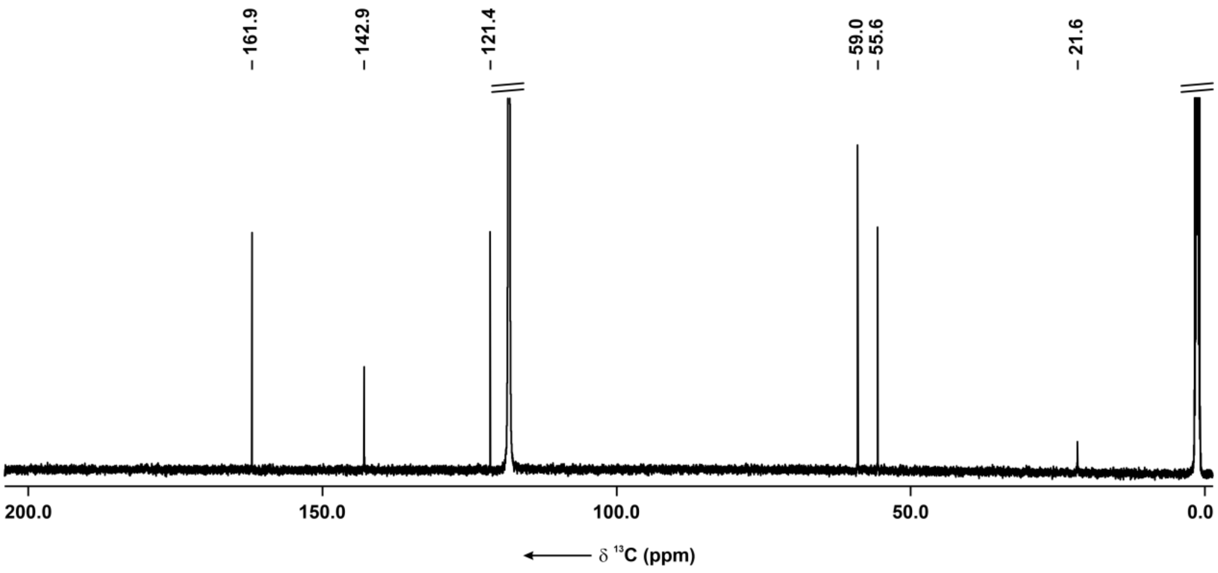

Figure S10. The ^{13}C NMR spectrum of **3-Cd$_2^{OAc}$** (151 MHz, CD_3CN, 300 K).

NMR spectra of 3-Hg$_2^{OTf}$ cage

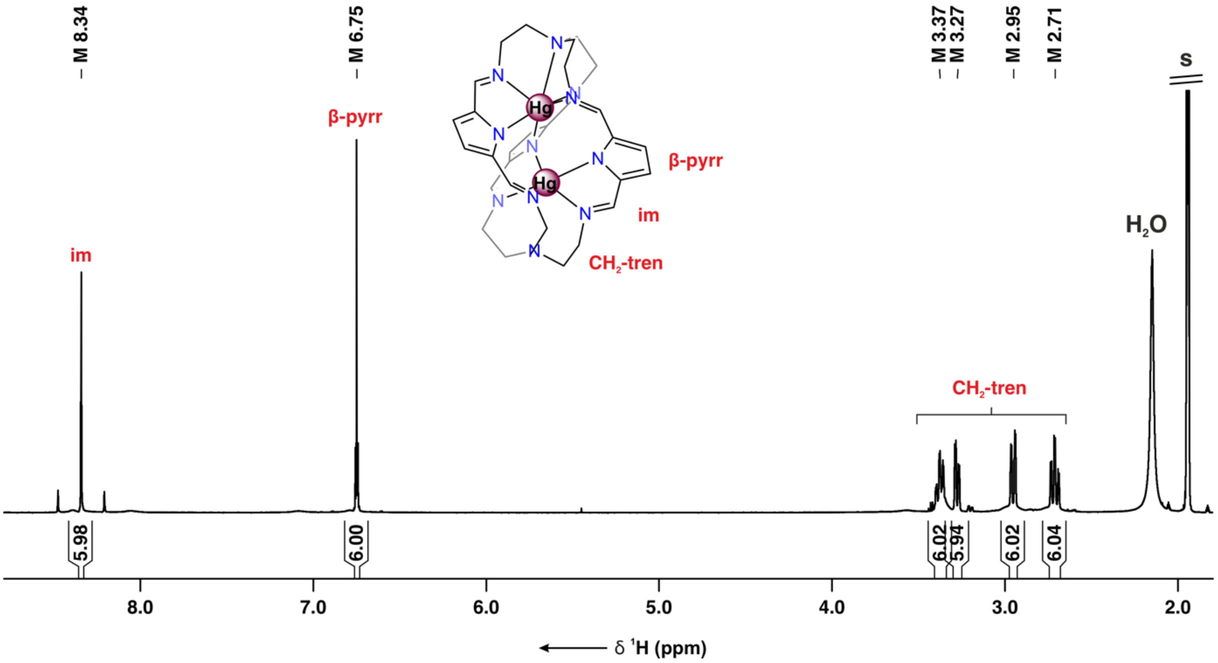

Figure S11. The ^{1}H NMR spectrum of **3-Hg$_2^{OTf}$** (600 MHz, CD_3CN, 300 K).

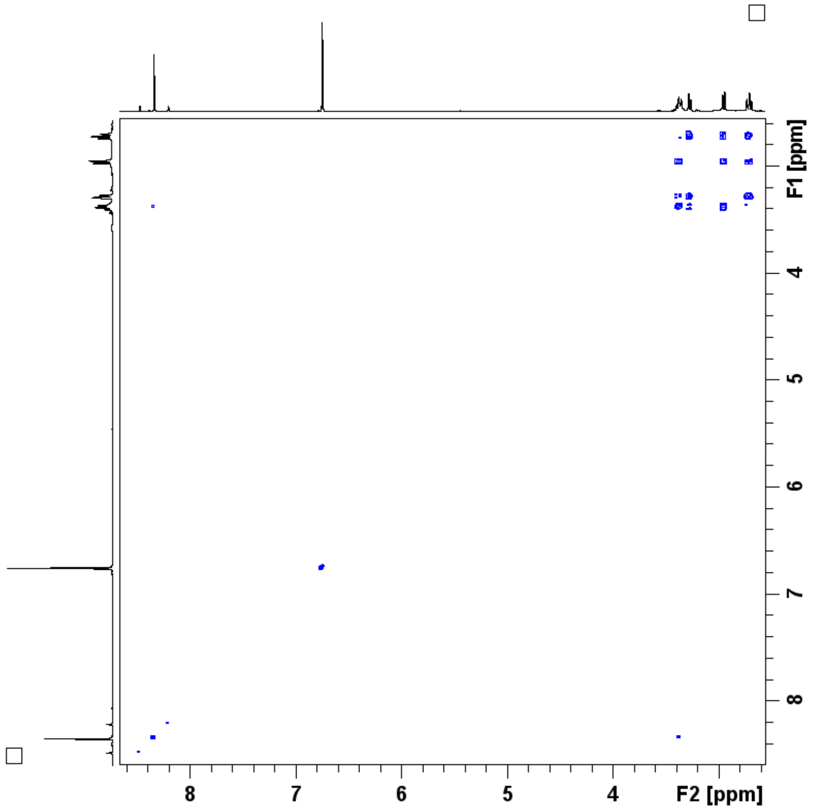

Figure S12. The ^{1}H-^{1}H COSY spectrum of **3-Hg$_2^{OTf}$** (600 MHz, CD_3CN, 300 K).

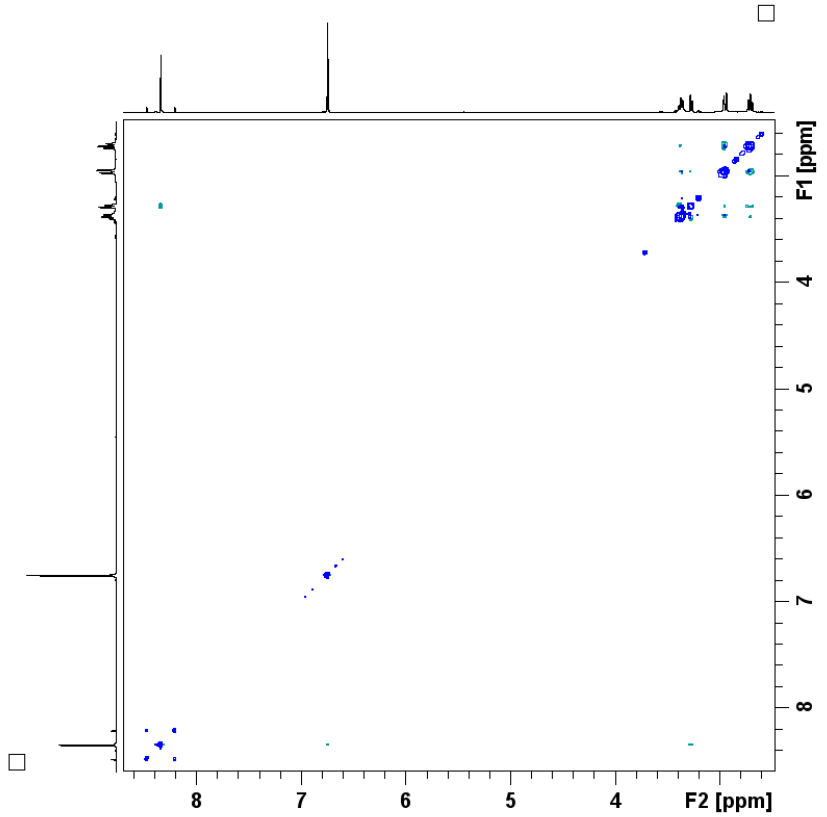

Figure S13. The ^{1}H-^{1}H NOESY spectrum of **3-Hg$_2$^{OTf}** (600 MHz, CD_3CN, 300 K).

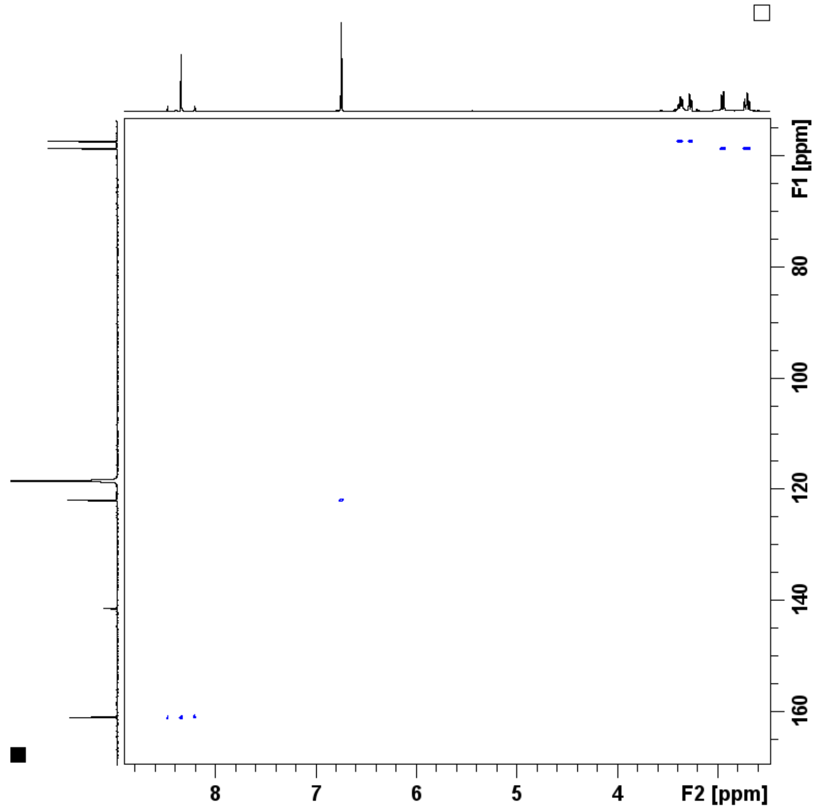

Figure S14. The ^{1}H-^{13}C HSQC spectrum of **3-Hg$_2$^{OTf}** (600 MHz, CD_3CN, 300 K).

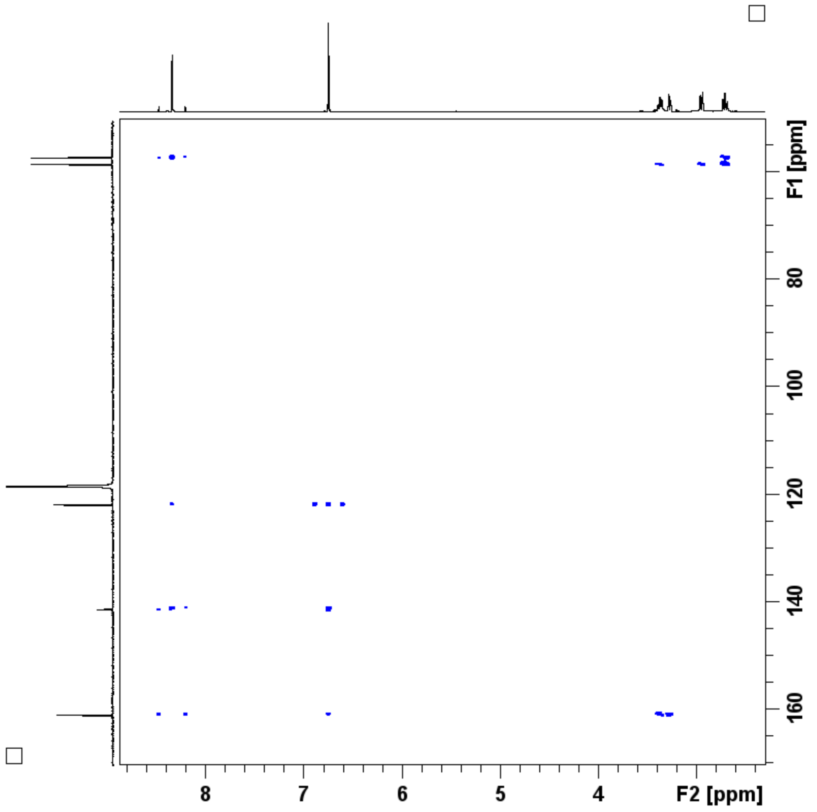

Figure S15. The ^1H-^{13}C HMBC spectrum of **3-Hg$_2$^{OTf}** (600 MHz, CD_3CN, 300 K).

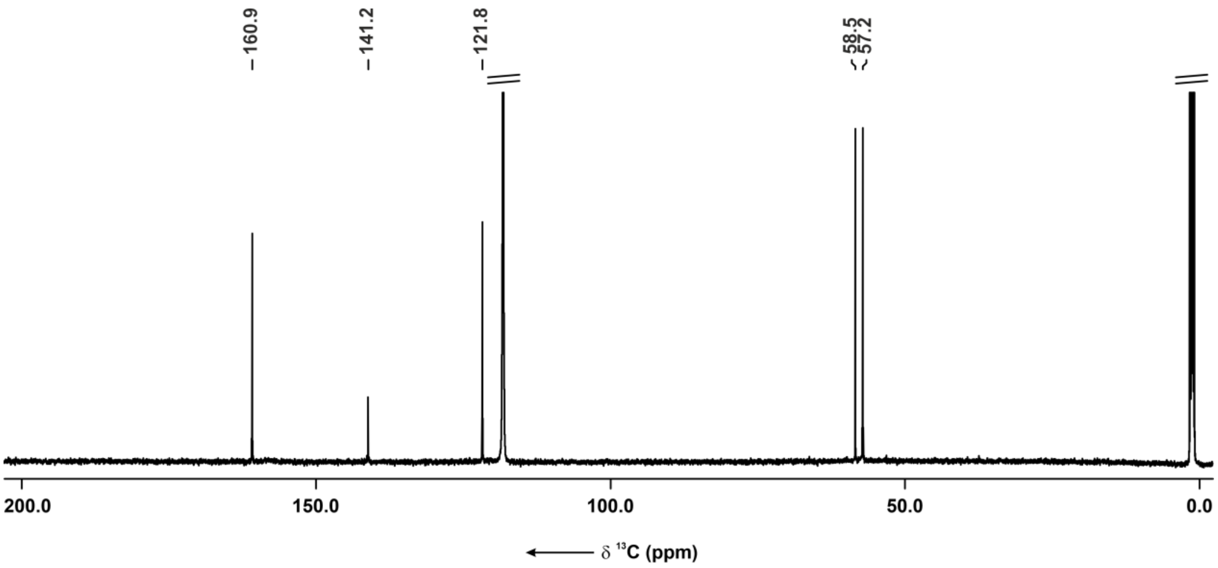

Figure S16. The ^{13}C NMR spectrum of **3-Hg$_2$^{OTf}** (151 MHz, CD_3CN, 300 K).

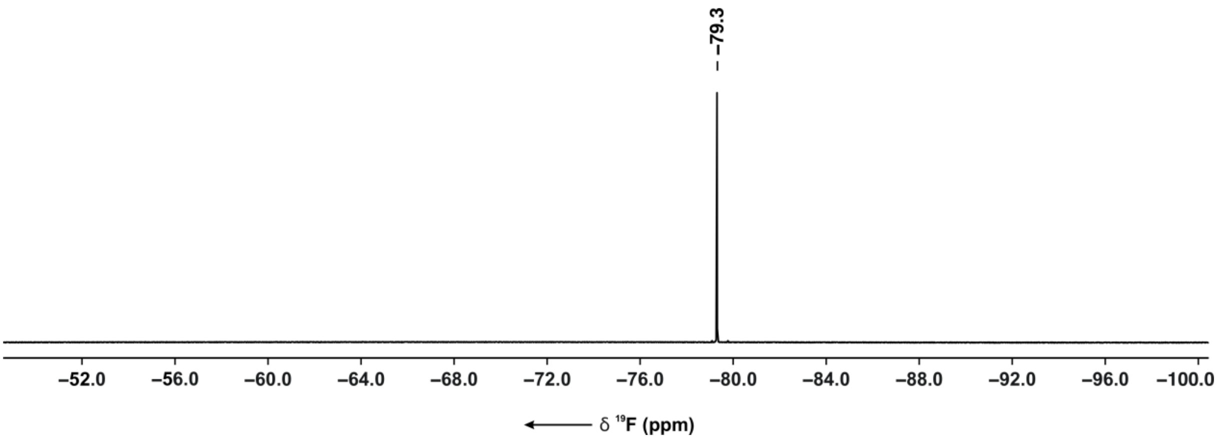

Figure S17. The ^{19}F NMR spectrum of **3-Hg$_2$^{OTf}** (471 MHz, CD_3CN, 300 K).

NMR spectra of 3-Cd^{OAc} cage

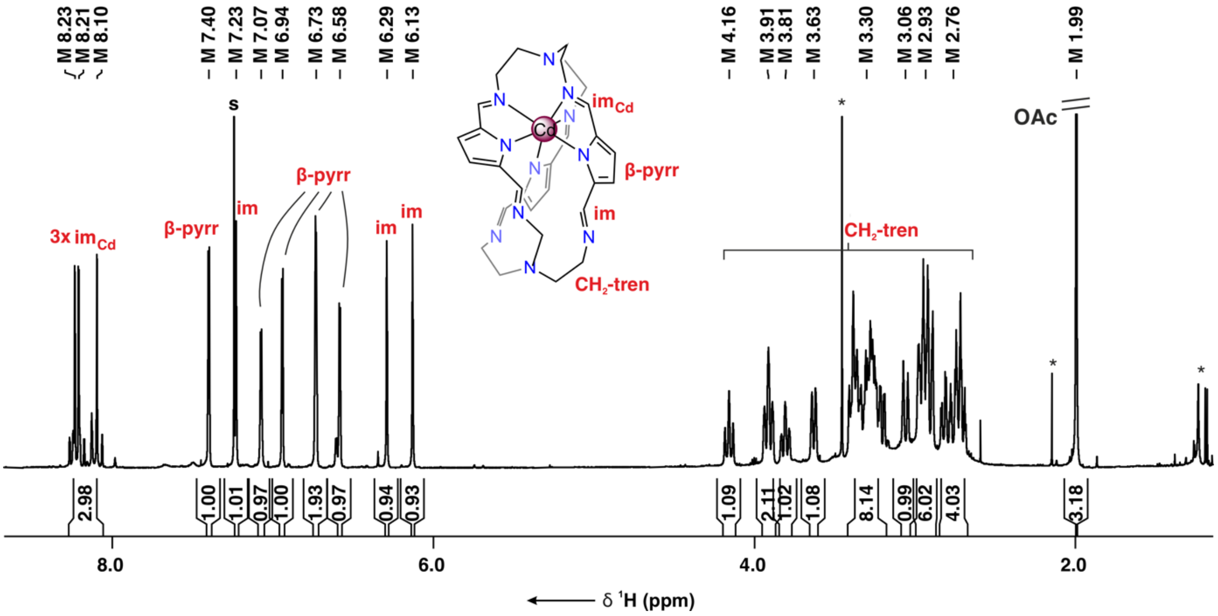

Figure S18. The ^{1}H NMR spectrum of **3-Cd^{OAc}** (500 MHz, $CDCl_3$, 300 K). Signals corresponding to impurities were marked with asterisks.

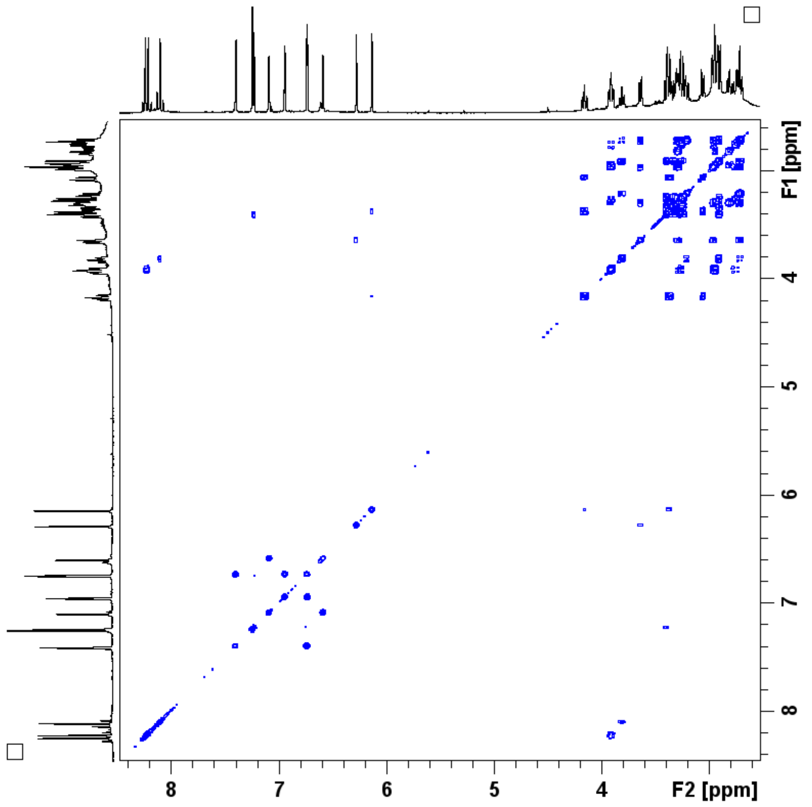

Figure S19. The ^{1}H-^{1}H COSY spectrum of **3-Cd^{OAc}** (600 MHz, $CDCl_3$, 300 K).

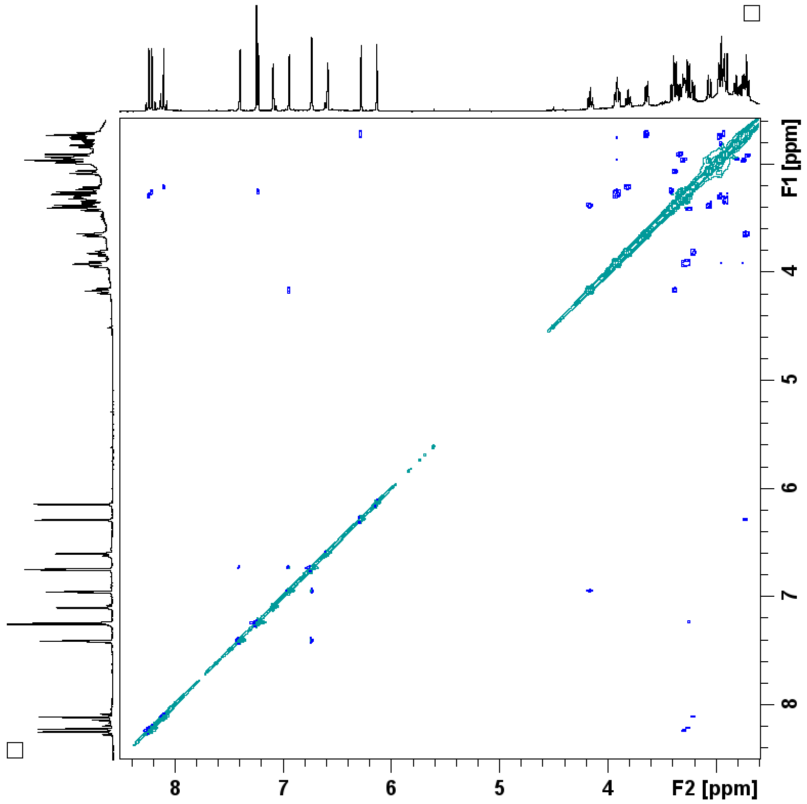

Figure S20. The ^{1}H-^{1}H NOESY spectrum of **3-Cd^{OAc}** (600 MHz, $CDCl_3$, 300 K).

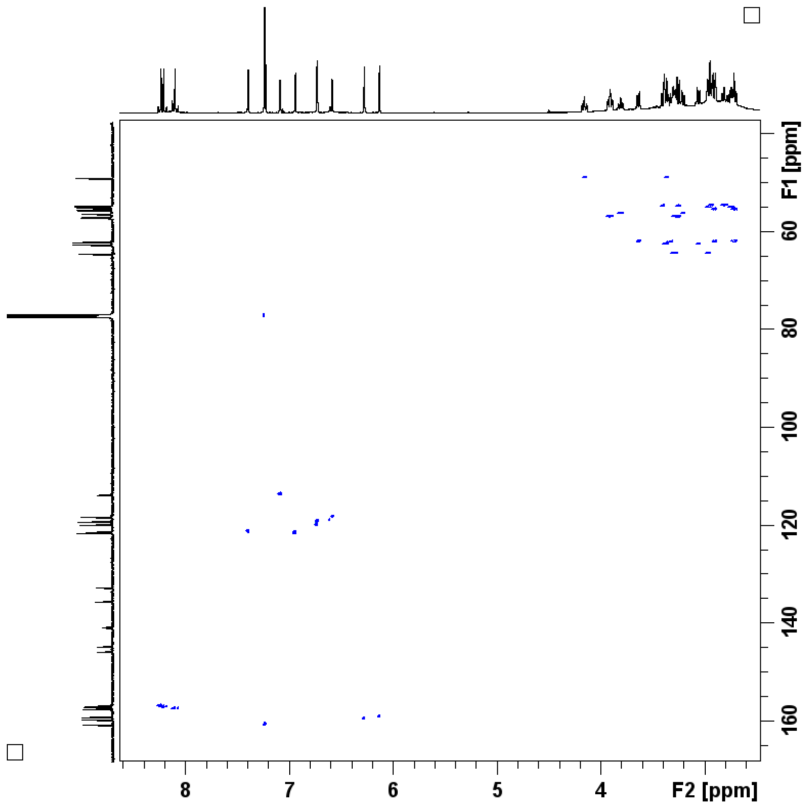

Figure S21. The ^{1}H-^{13}C HSQC spectrum of **3-Cd^{OAc}** (600 MHz, $CDCl_3$, 300 K).

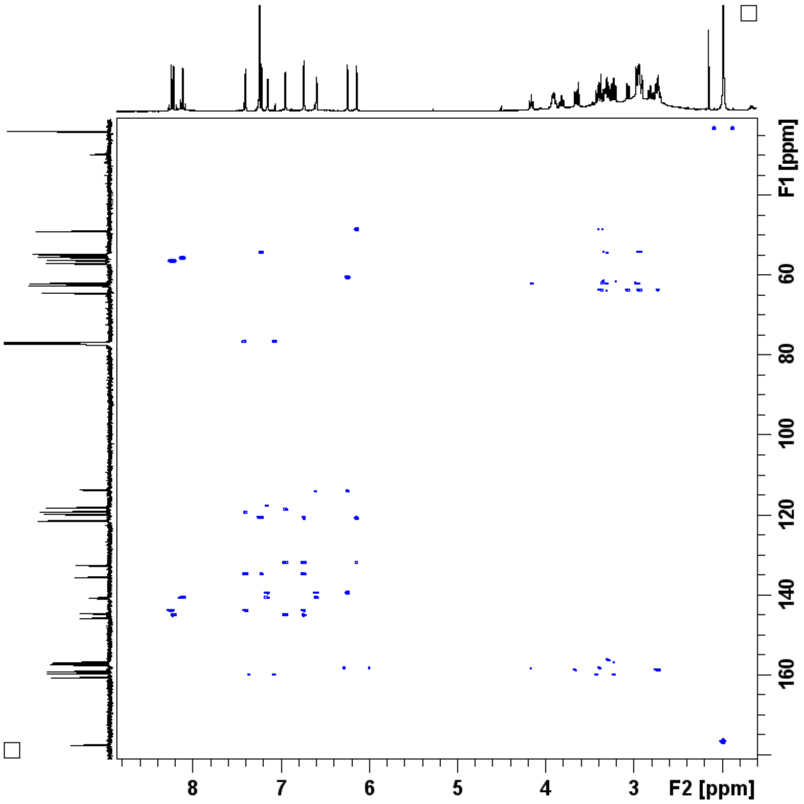

Figure S22. The ^{1}H-^{13}C HMBC spectrum of **3-Cd^{OAc}** (600 MHz, $CDCl_3$, 300 K).

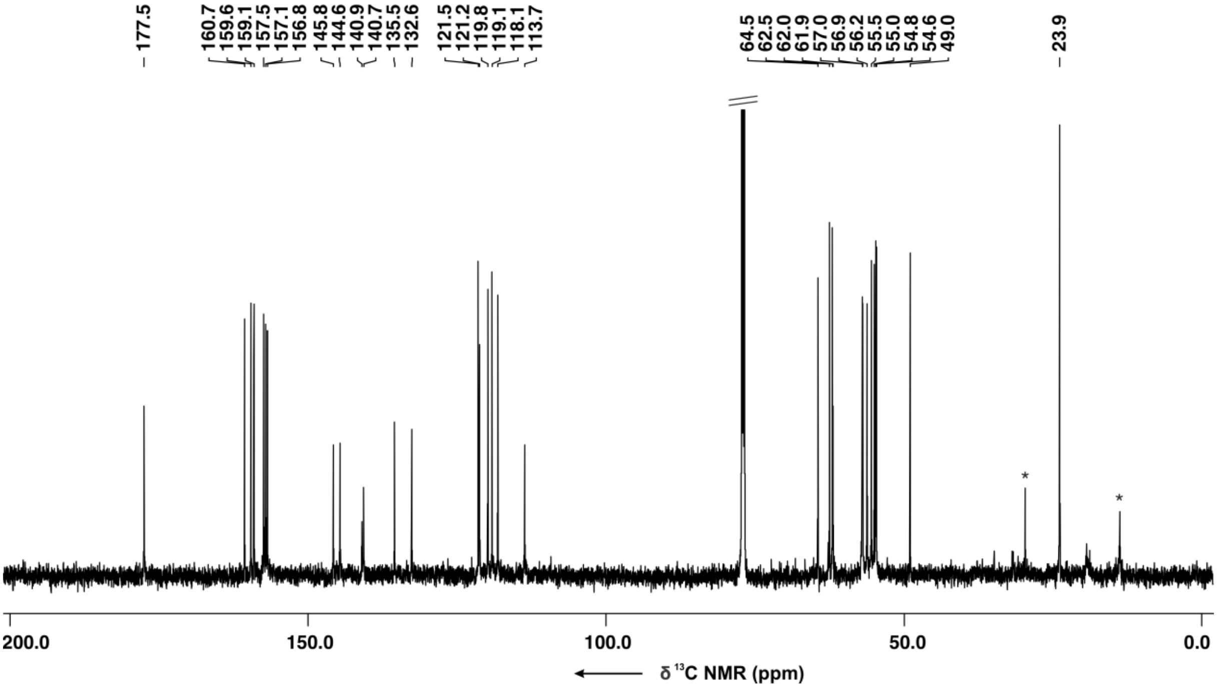

Figure S23. The ^{13}C NMR spectrum of **3-Cd^{OAc}** (151 MHz, $CDCl_3$, 300 K).

NMR spectra of 3-CdZn^{PF6} cage

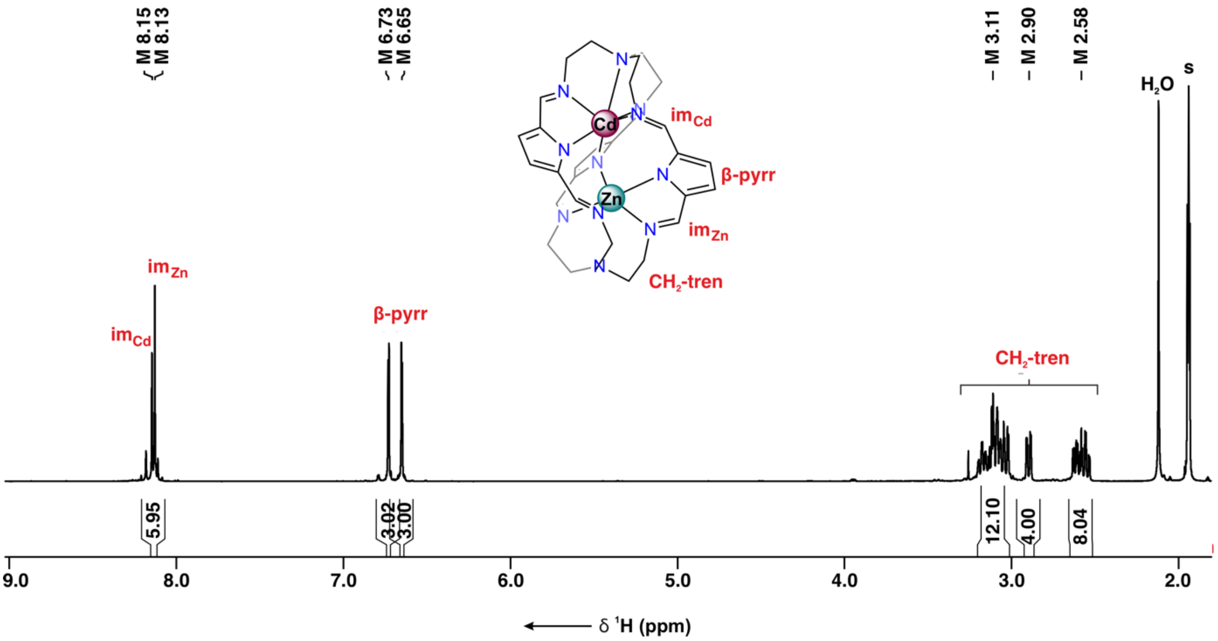

Figure S24. The ^{1}H NMR spectrum of **3-CdZn^{PF6}** (600 MHz, CD3CN, 300 K).

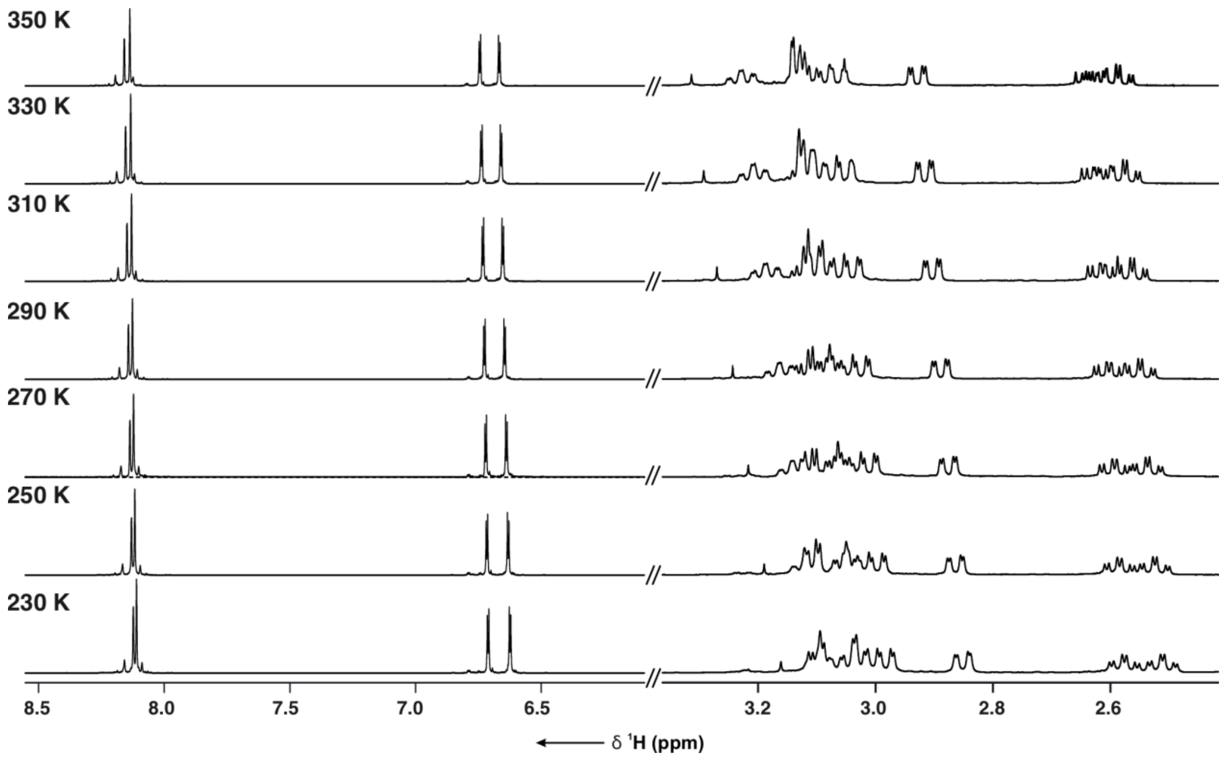

Figure S25. The ^{1}H NMR spectra of **3-CdZn^{PF6}** recorded in the 350 K – 230 K temperature range (600 MHz, CD3CN).

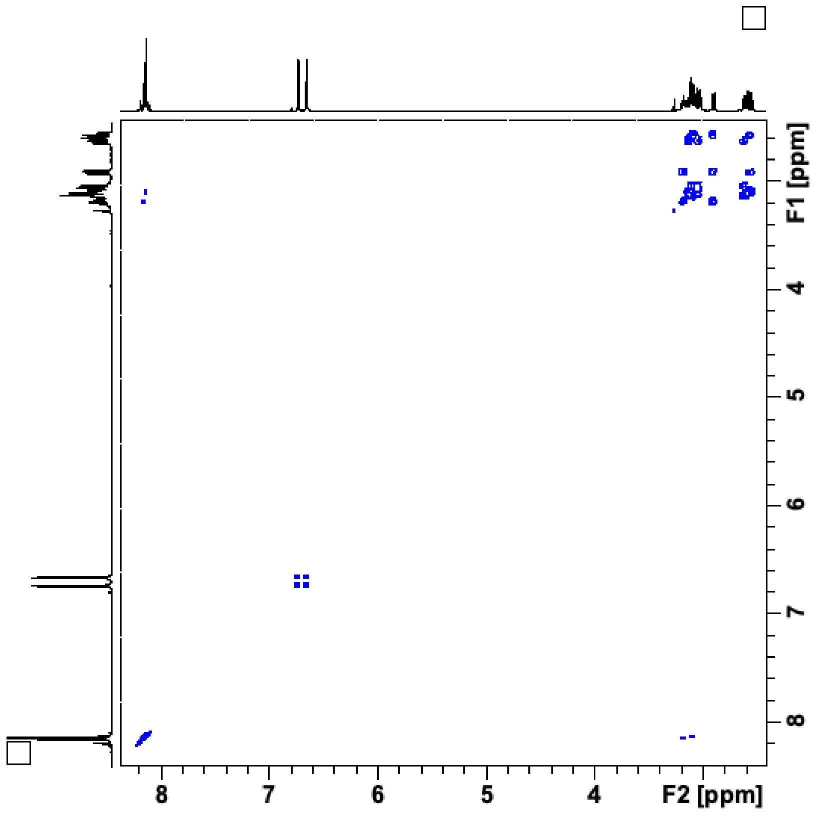

Figure S26. The ^{1}H-^{1}H COSY spectrum of **3-CdZn^{PF6}** (600 MHz, CD_3CN, 300 K).

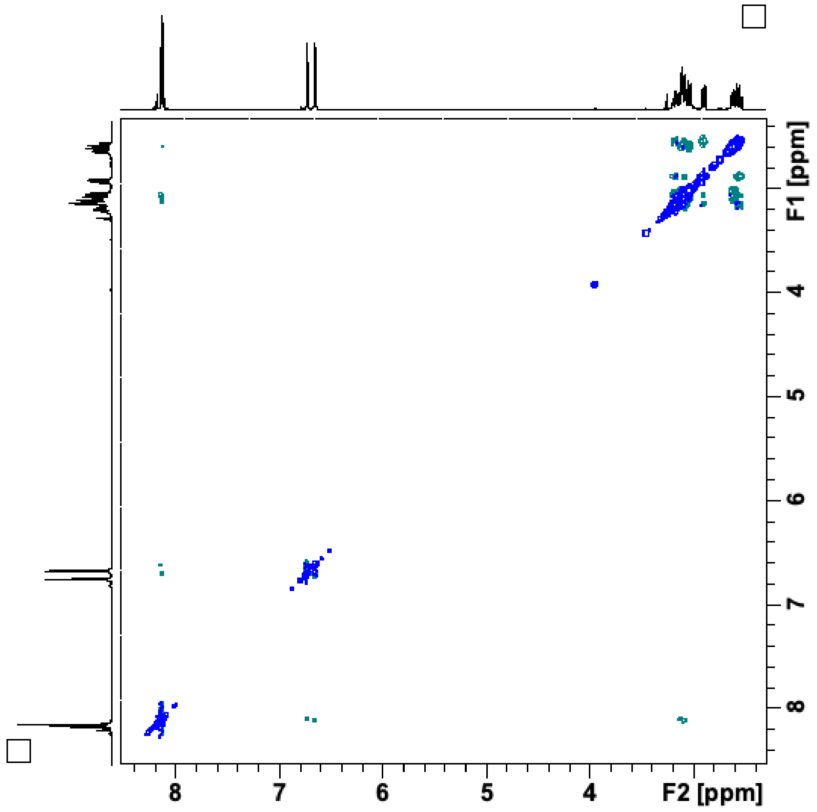

Figure S27. The ^{1}H-^{1}H NOESY spectrum of **3-CdZn^{PF6}** (600 MHz, CD_3CN, 300 K).

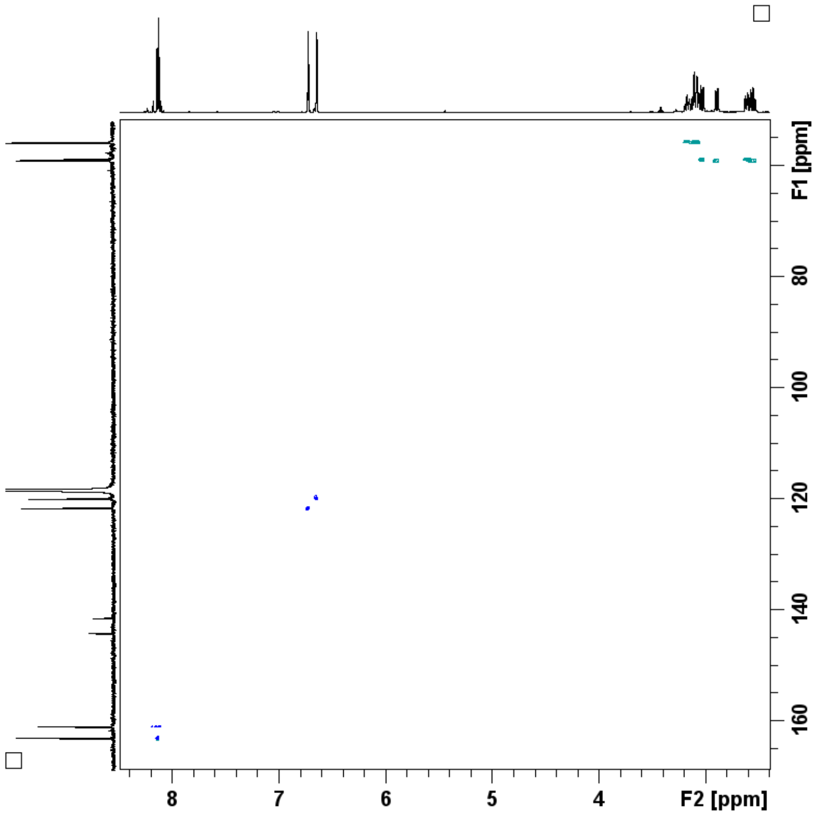

Figure S28. The ^{1}H-^{13}C HSQC spectrum of **3-CdZn^{PF6}** (600 MHz, CD_3CN, 300 K).

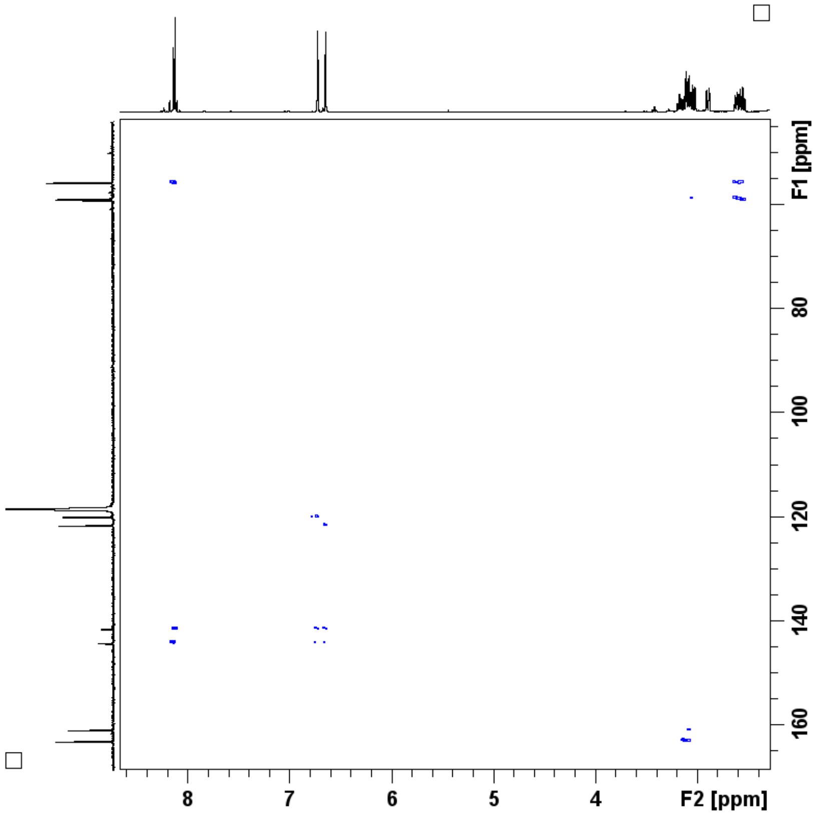

Figure S29. The ^{1}H-^{13}C HMBC spectrum of **3-CdZn^{PF6}** (600 MHz, CD_3CN, 300 K).

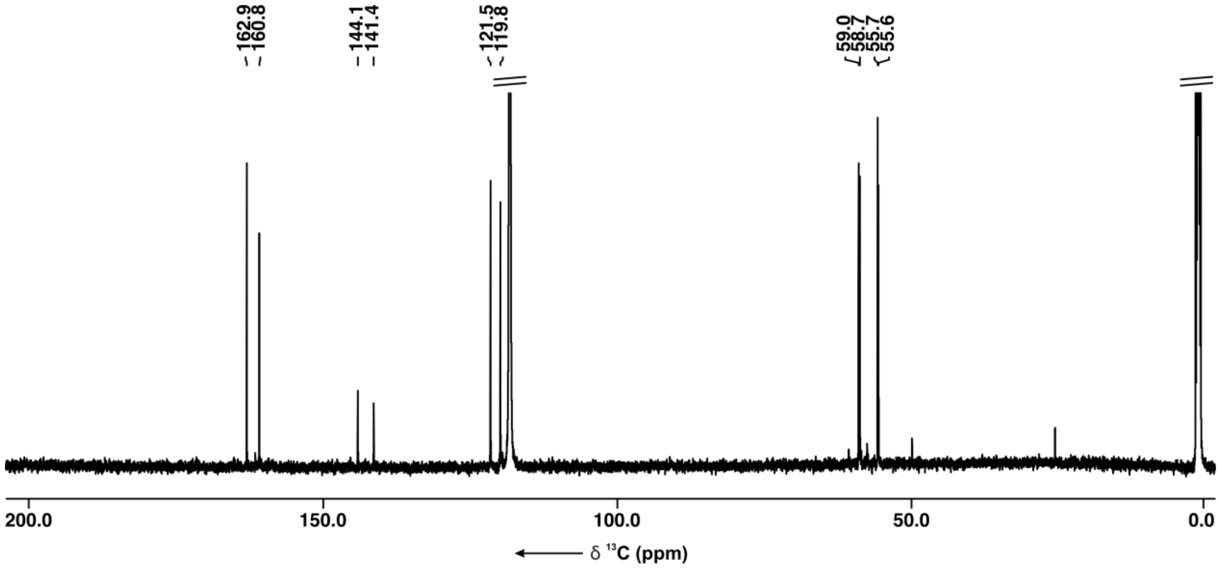

Figure S30. The ^{13}C NMR spectrum of **3-CdZn^{PF6}** (151 MHz, CD_3CN, 300 K).

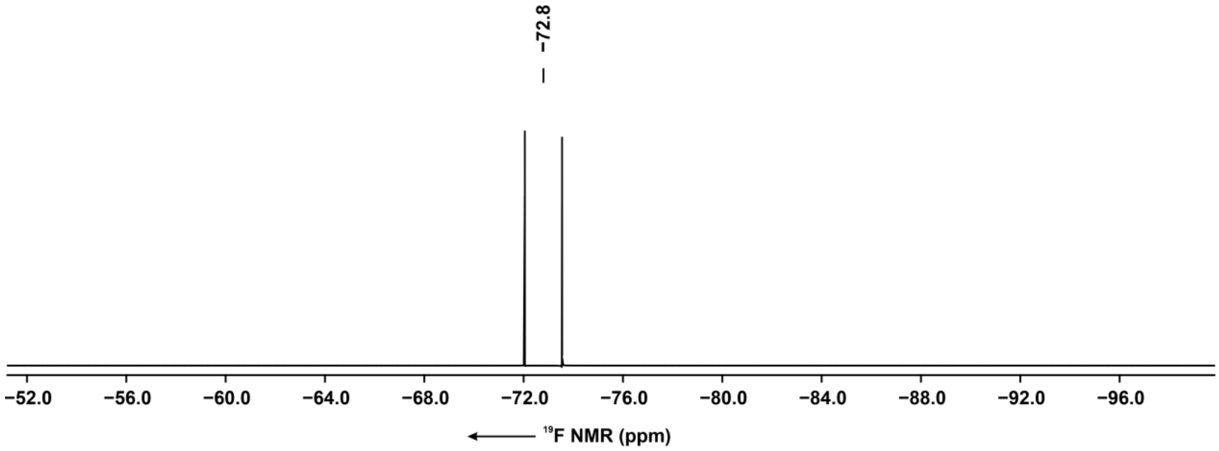

Figure S31. The ^{19}F NMR spectrum of **3-CdZn^{PF6}** (471 MHz, CD_3CN, 300 K).

NMR spectra of 3-CdHg^{PF6} cage

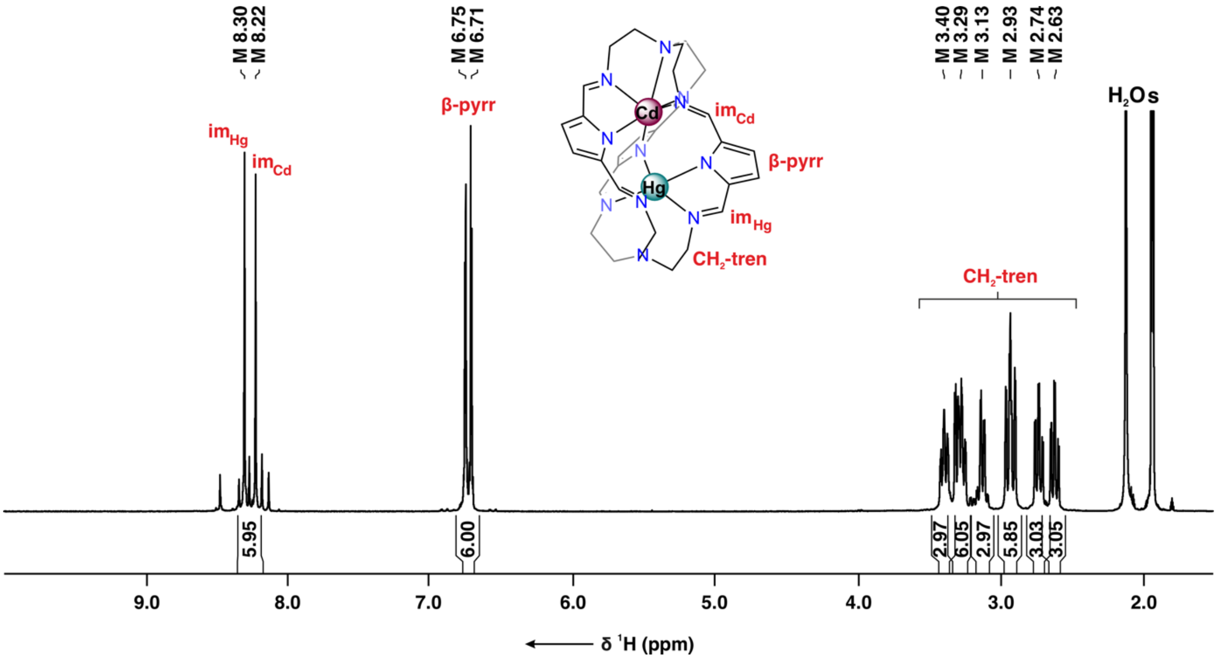

Figure S32. The ^{1}H NMR spectrum of **3-CdHg^{PF6}** (600 MHz, CD_3CN, 300 K).

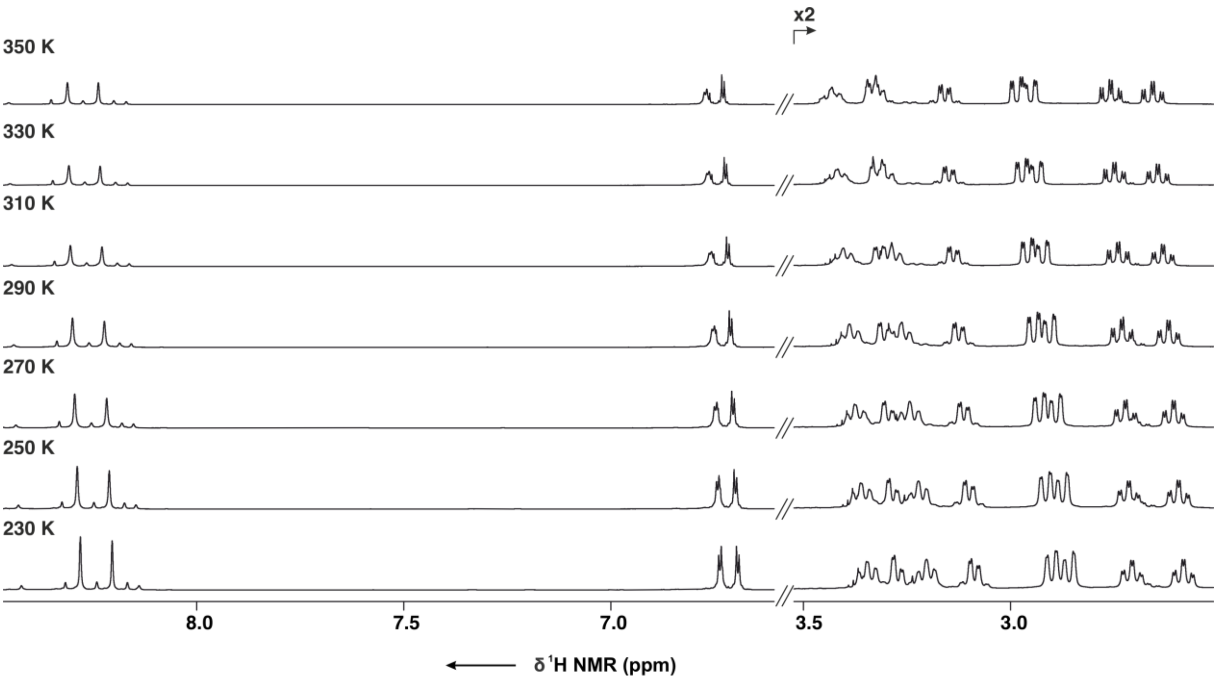

Figure S33. The ^{1}H NMR spectra of **3-CdHg^{PF6}** recorded in the 350 K – 230 K temperature range (600 MHz, CD_3CN).

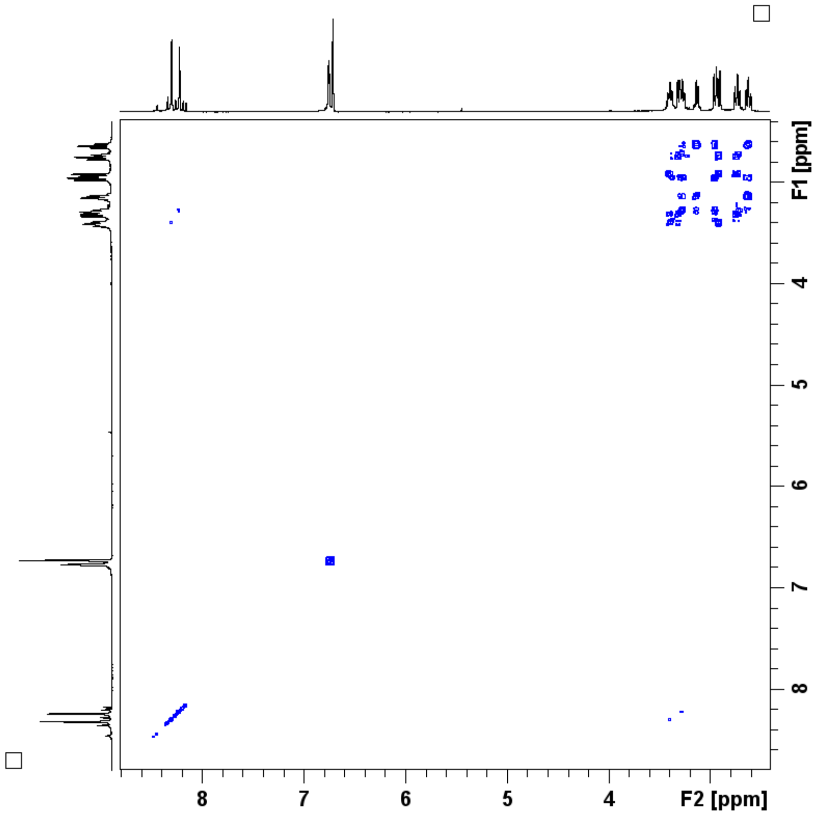

Figure S34. The ^{1}H-^{1}H COSY spectrum of **3-CdHg^{PF6}** (600 MHz, CD_3CN, 300 K).

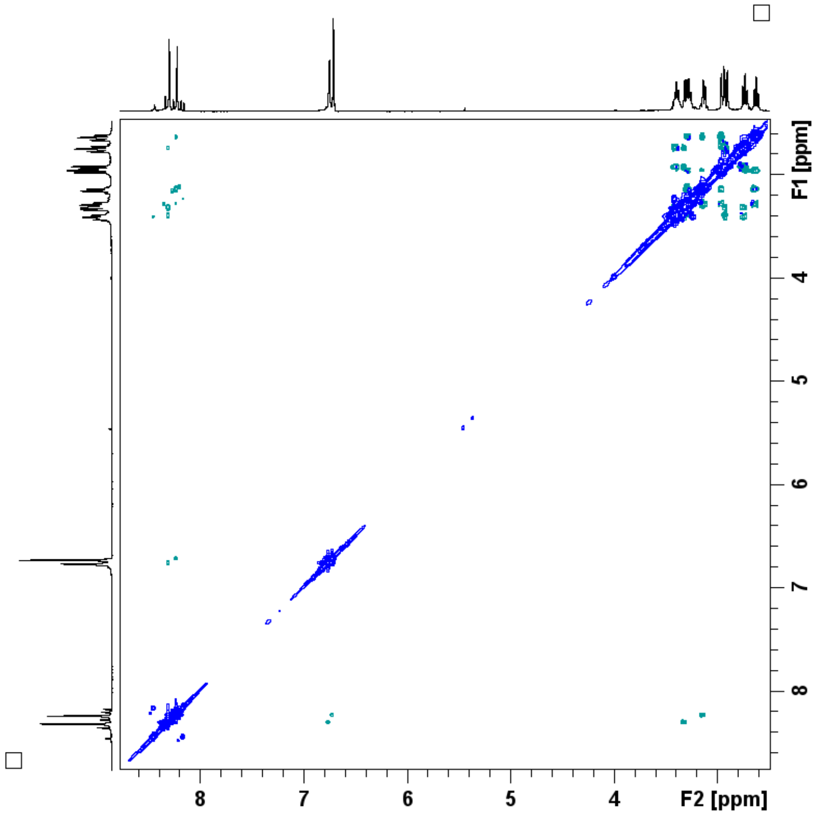

Figure S35. The ^{1}H-^{1}H NOESY spectrum of **3-CdHg^{PF6}** (600 MHz, CD_3CN, 300 K).

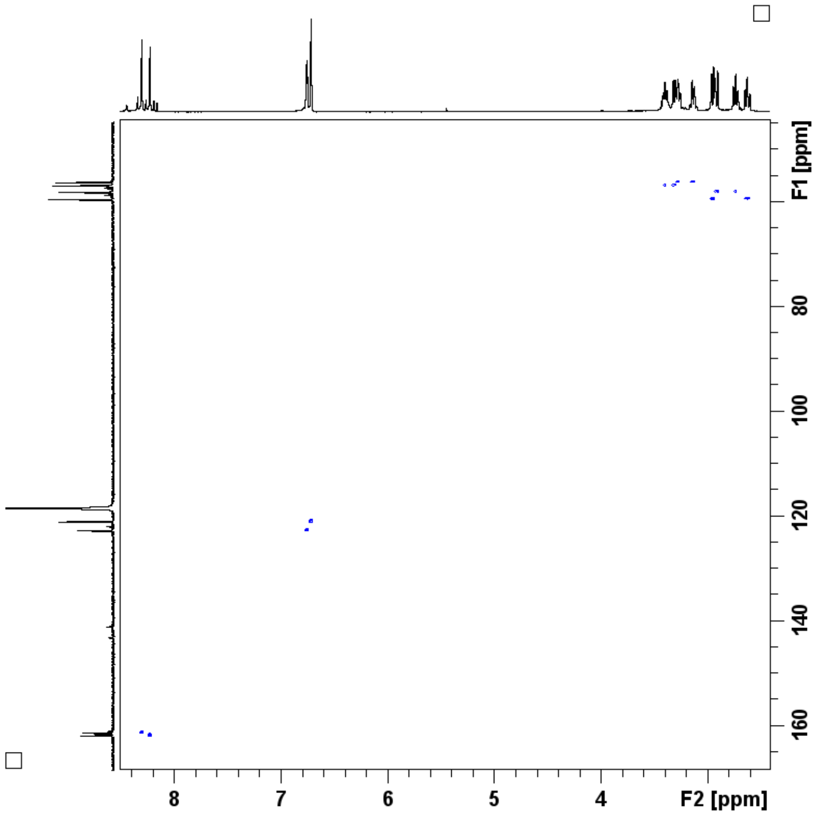

Figure S36. The ^{1}H-^{13}C HSQC spectrum of **3-CdHg^{PF6}** (600 MHz, CD_3CN, 300 K).

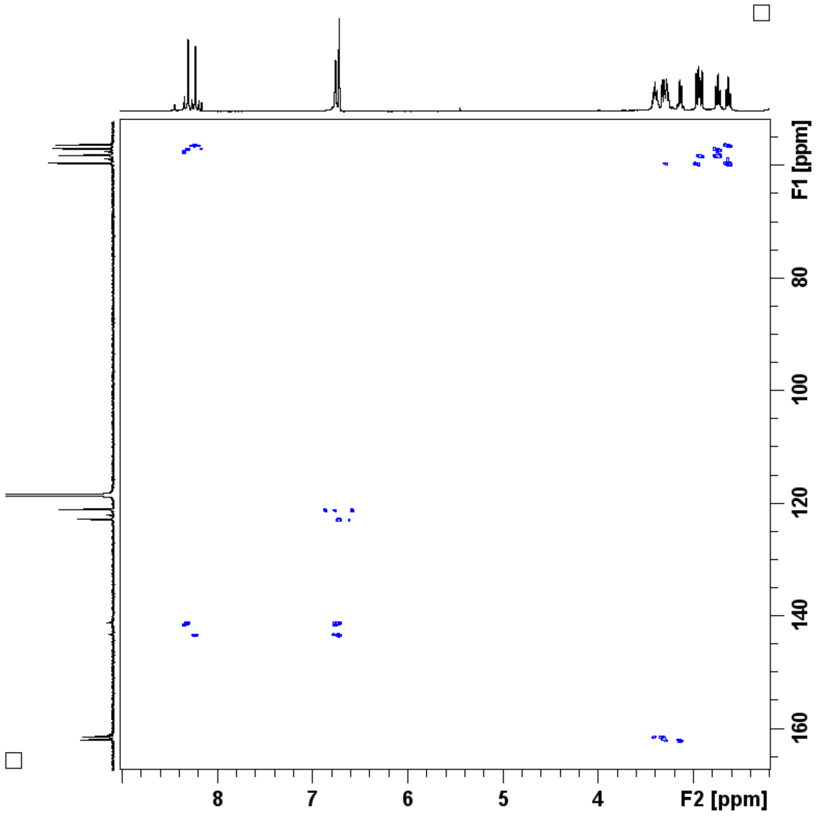

Figure S37. The ^{1}H-^{13}C HMBC spectrum of **3-CdHg^{PF6}** (600 MHz, CD_3CN, 300 K).

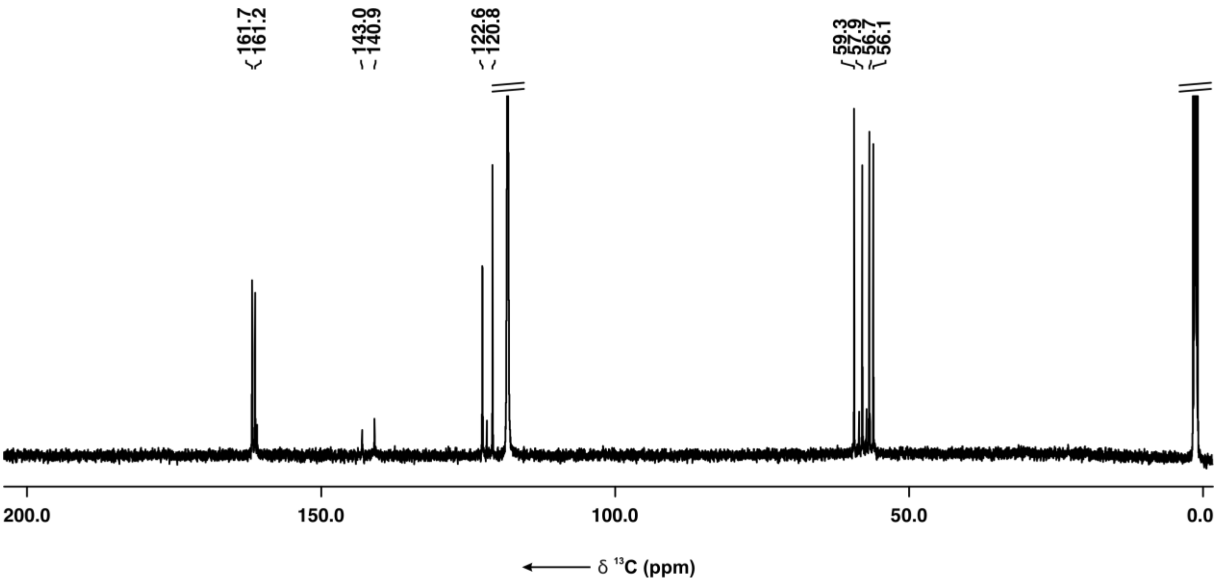

Figure S38. The ^{13}C NMR spectrum of **3-CdHg^{PF6}** (151 MHz, CD_3CN, 300 K).

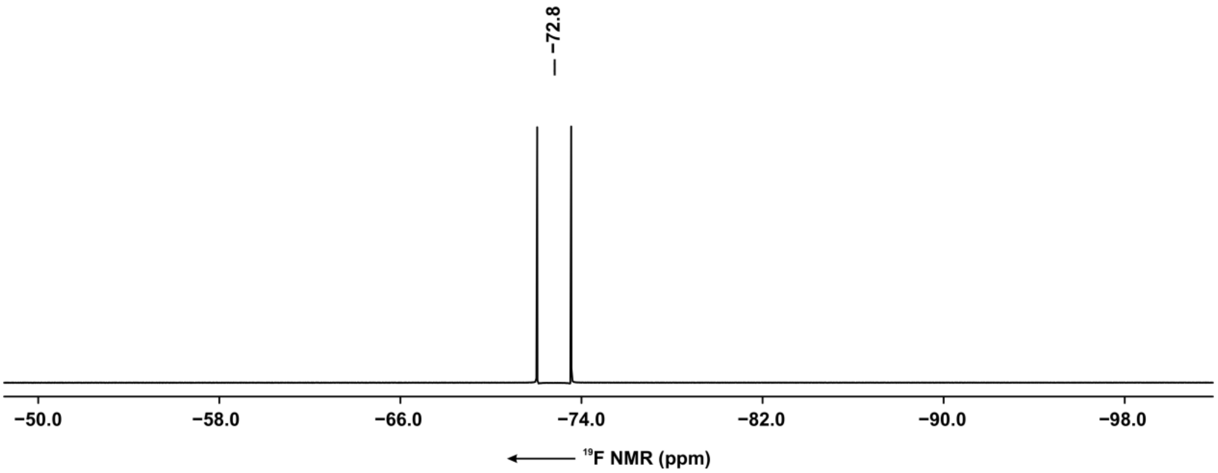

Figure S39. The ^{19}F NMR spectrum of **3-CdHg^{PF6}** (471 MHz, CD_3CN, 300 K).

High-resolution mass spectra

Mass spectra of 3-Zn_2^{PF6} cage

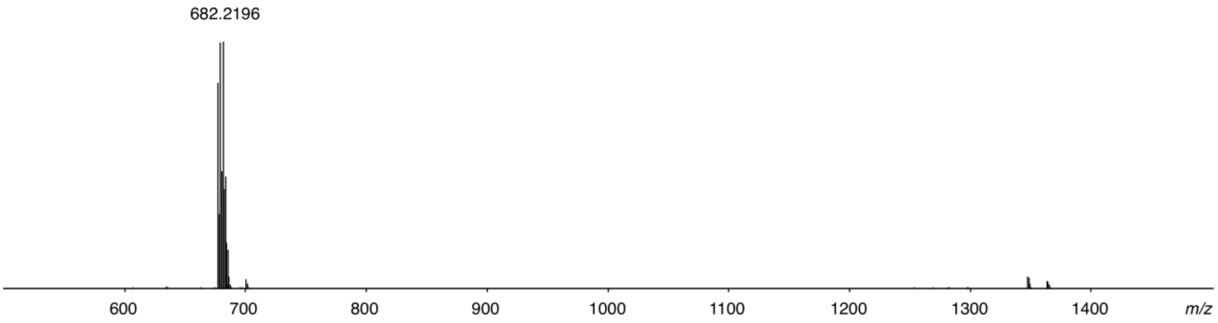

Figure S40. The 500-1500 *m/z* range of the low-resolution mass spectrum of **3-Zn_2^{PF6}** (ESI+, TOF).

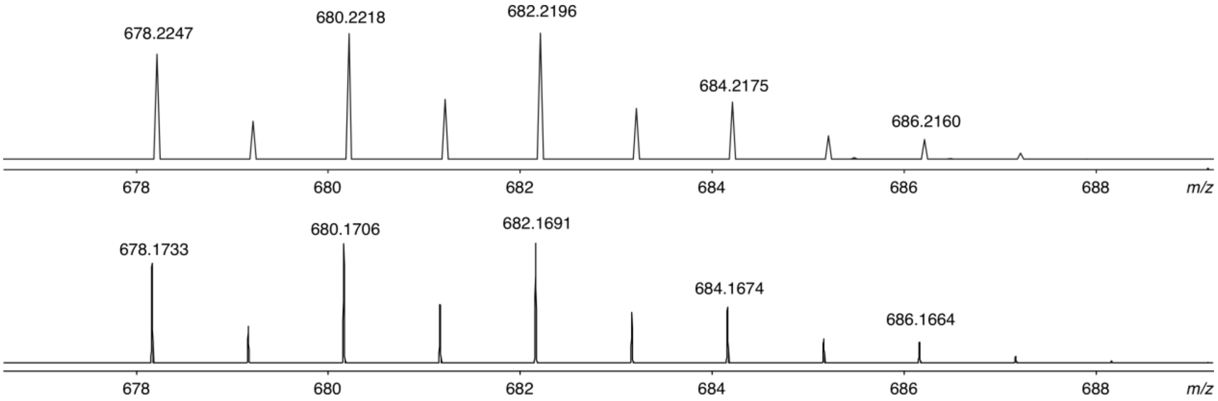

Figure S41. The low-resolution mass spectrum of **3-Zn_2^{PF6}** (ESI+, TOF, $[C_{30}H_{36}N_{11}Zn_2]^+$). Top: experimental spectrum, bottom: simulated pattern.

Mass spectra of 3-Cd_2^{OAc} cage

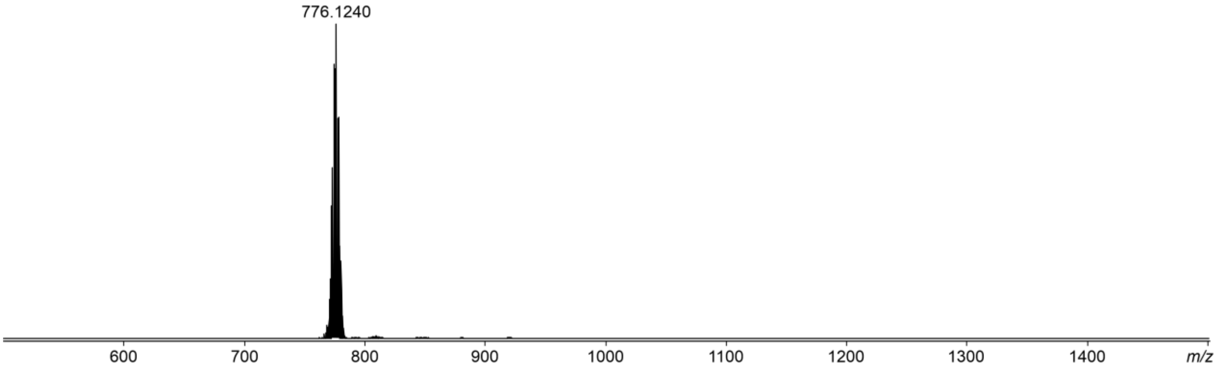

Figure S42. The 500-1500 *m/z* range of the high-resolution mass spectrum of **3-Cd_2^{OAc}** (ESI+, TOF).

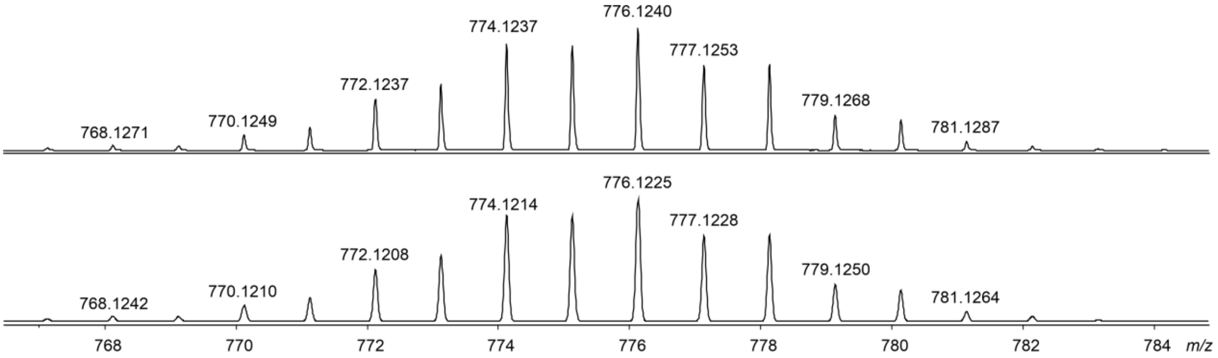

Figure S43. The high-resolution mass spectrum of **3-Cd_2^{OAc}** (ESI+, TOF, $[C_{30}H_{36}N_{11}Cd_2]^+$). Top: experimental spectrum, bottom: simulated pattern.

Mass spectra of 3-Hg_2^{OTf} cage

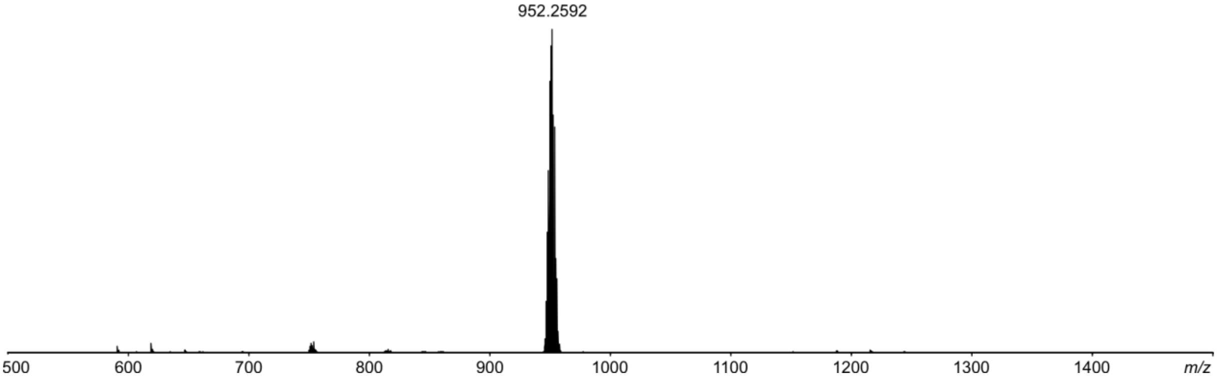

Figure S44. The 500-1500 *m/z* range of the high-resolution mass spectrum of **3-Hg_2^{OTf}** (ESI+, TOF).

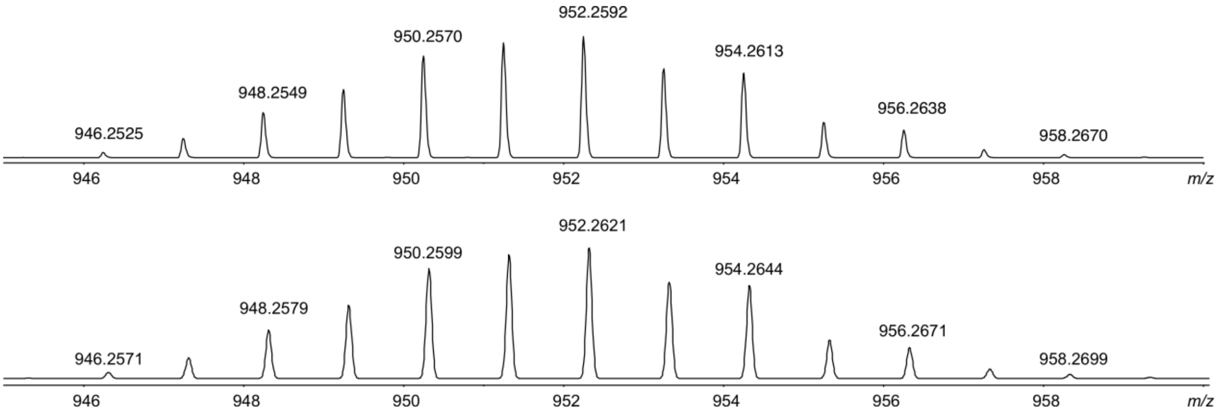

Figure S45. The high-resolution mass spectrum of **3-Hg_2^{OTf}** (ESI+, TOF, $[C_{30}H_{36}N_{11}Hg_2]^+$). Top: experimental spectrum, bottom: simulated pattern.

Mass spectra of 3-Cd^{OAc} cage

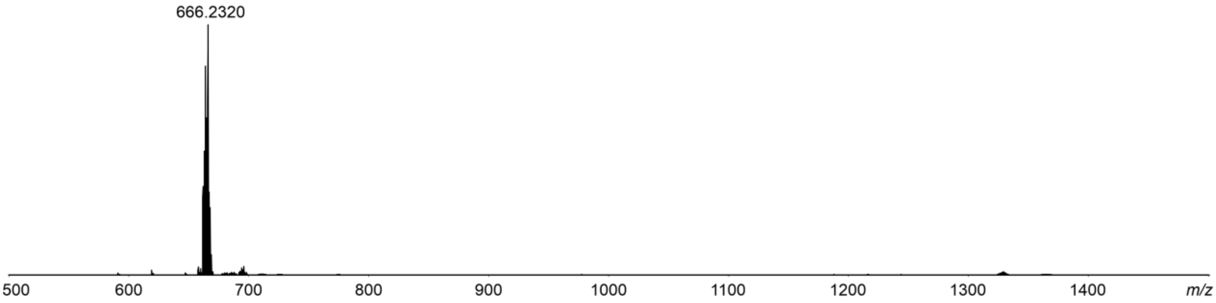

Figure S46. The 500-1500 *m/z* range of the high-resolution mass spectrum of **3-Cd^{OAc}** (ESI+, TOF).

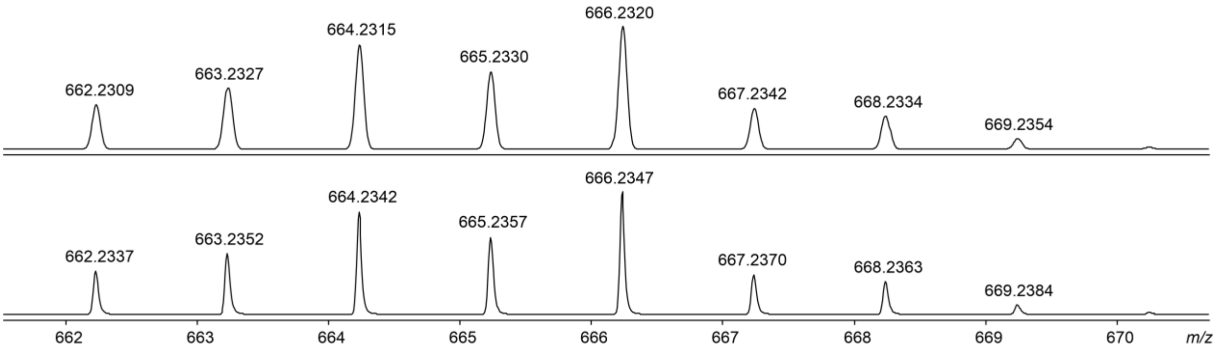

Figure S47. The high-resolution mass spectrum of **3-Cd^{OAc}** (ESI+, TOF, $[C_{30}H_{36}N_{11}Cd+2H]^+$). Top: experimental spectrum, bottom: simulated pattern.

Mass spectra of 3-CdZn^{PF6} cage

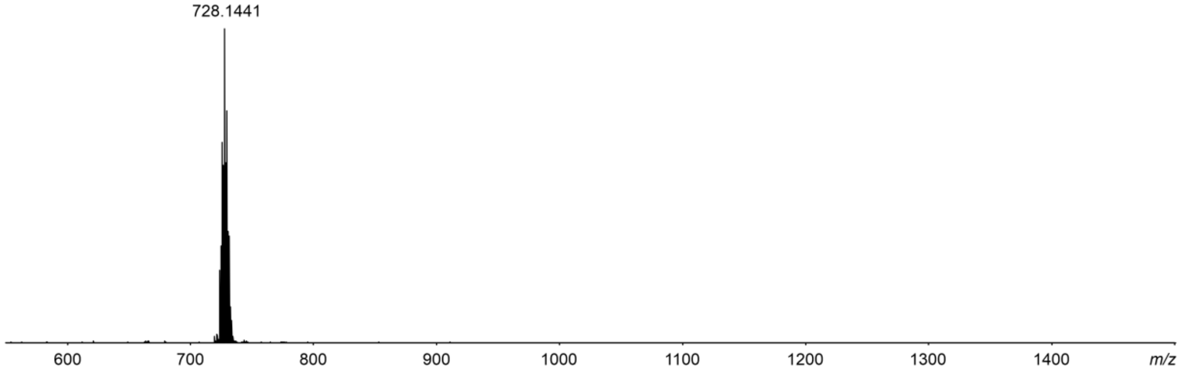

Figure S48. The 500-1500 *m/z* range of the high-resolution mass spectrum of **3-CdZn^{PF6}** (ESI+, TOF).

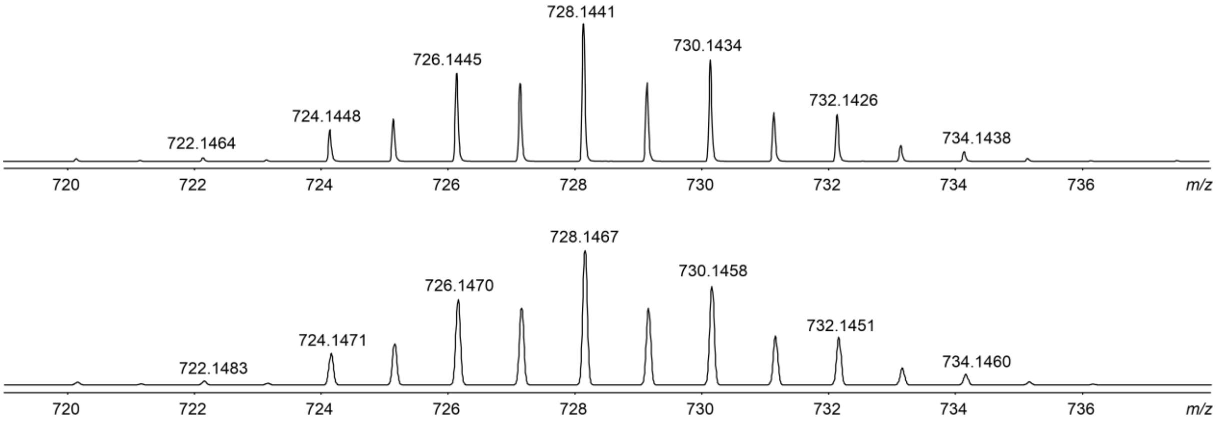

Figure S49. The high-resolution mass spectrum of **3-CdZn^{PF6}** (ESI+, TOF, $[C_{30}H_{36}N_{11}CdZn]^{+}$). Top: experimental spectrum, bottom: simulated pattern.

Mass spectra of 3-CdHg^{PF6} cage

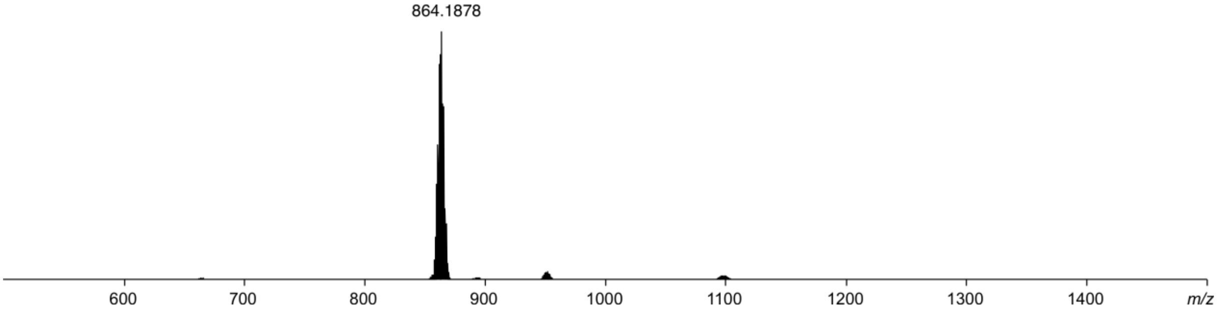

Figure S50. The 500-1500 *m/z* range of the high-resolution mass spectrum of **3-CdHg^{PF6}** (ESI+, TOF).

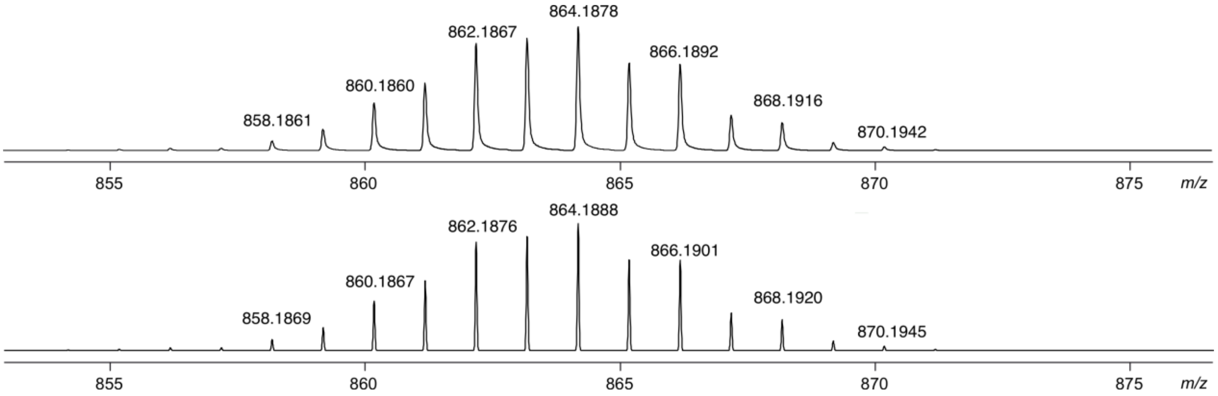

Figure S51. The high-resolution mass spectrum of **3-CdHg^{PF6}** (ESI+, TOF, $[C_{30}H_{36}N_{11}CdHg]^+$). Top: experimental spectrum, bottom: simulated pattern.

Additional X-ray molecular structures

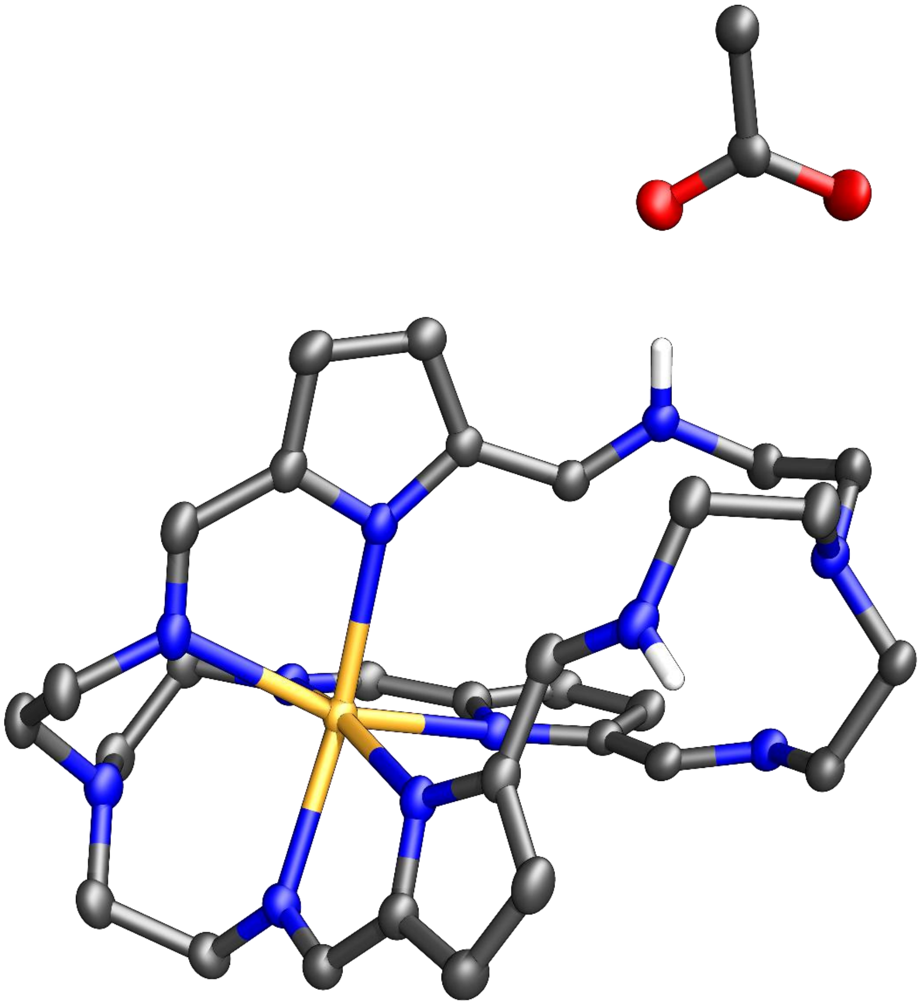

Figure S52. The crystal structure of **H_2[3-Cd]OAc.** Carbon-bound hydrogen atoms and solvent molecules were omitted for clarity. The thermal ellipsoids were depicted at 50% probability level.

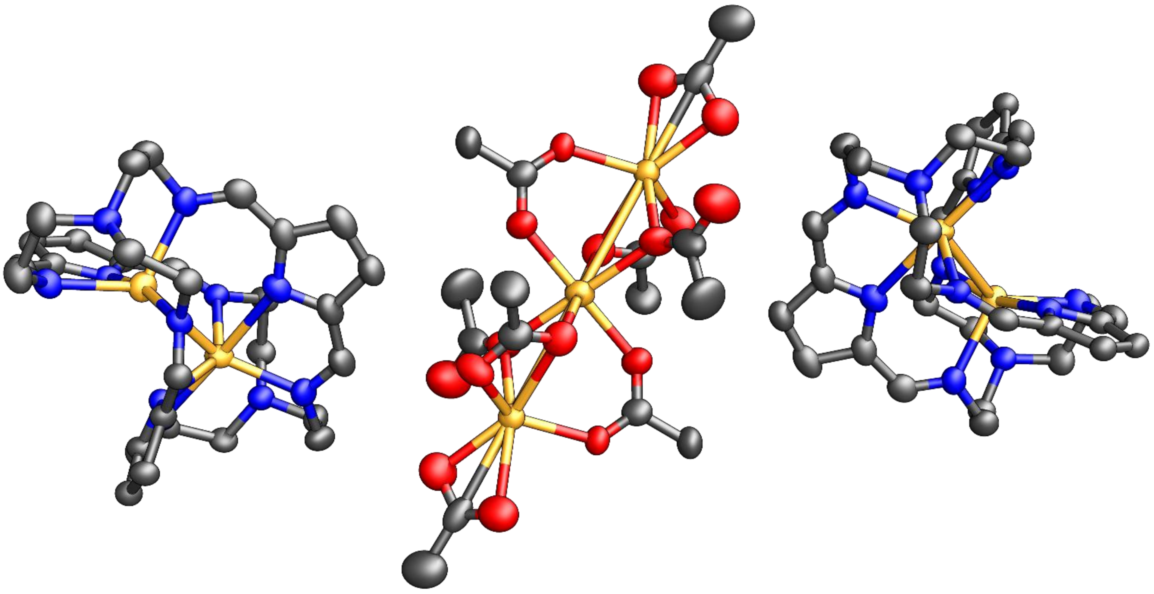

Figure S53. The crystal structure of **[3-Cd$_2$]$_2$[Cd$_3$(OAc)$_8$]**. Hydrogen atoms and solvent molecules were omitted for clarity. The thermal ellipsoids were depicted at 50% probability level.

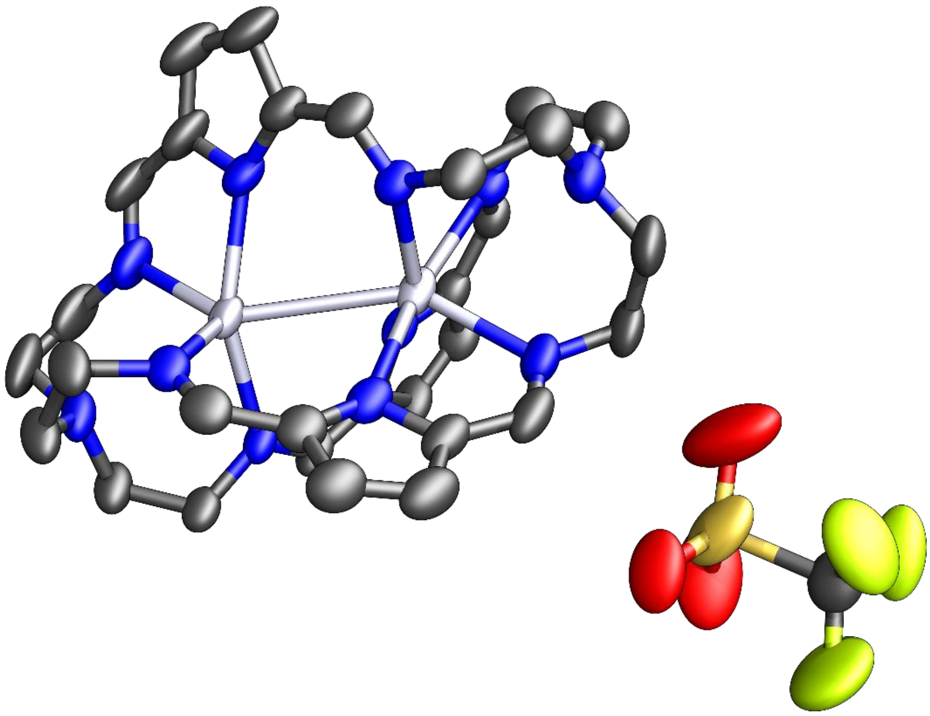

Figure S54. The crystal structure of **[3-Hg$_2$]OTf**. Hydrogen atoms and the minor disordered component of the triflate were omitted for clarity. The thermal ellipsoids were depicted at 50% probability level.

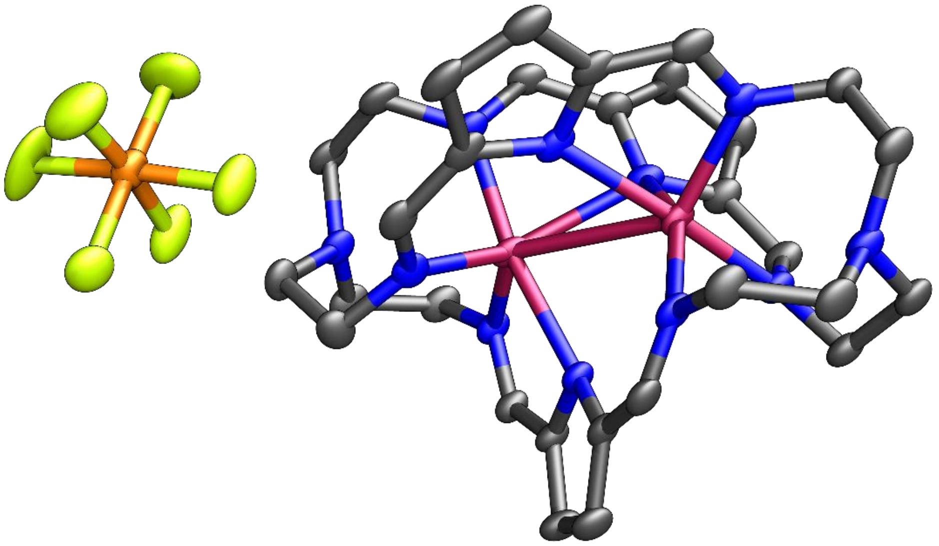

Figure S55. The crystal structure of **[3-Zn_2]PF_6**. Hydrogen atoms, solvent molecules, and the minor disordered component of the hexafluorophosphate were omitted for clarity. The thermal ellipsoids were depicted at 50% probability level.

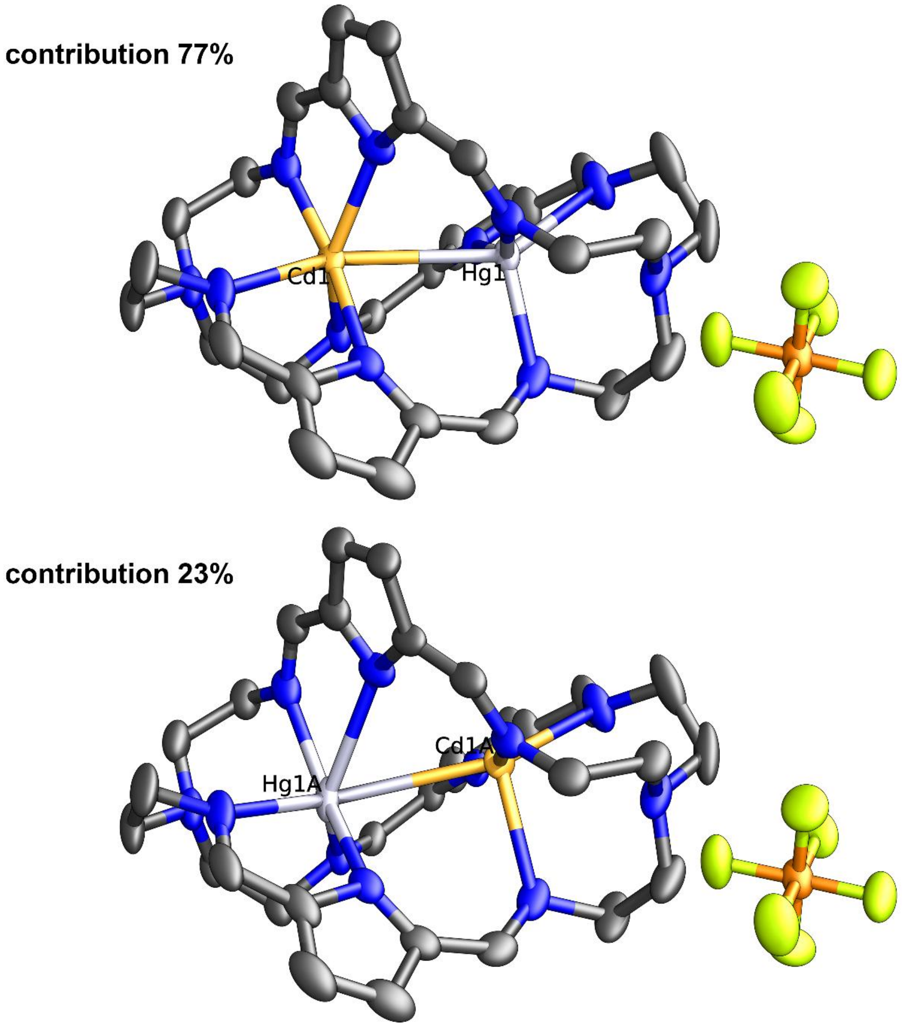

Figure S56. The crystal structure of **[3-CdHg]PF$_6$** with two modes of metal coordination present in the crystal. Hydrogen atoms were omitted for clarity. The thermal ellipsoids were depicted at 50% probability level.

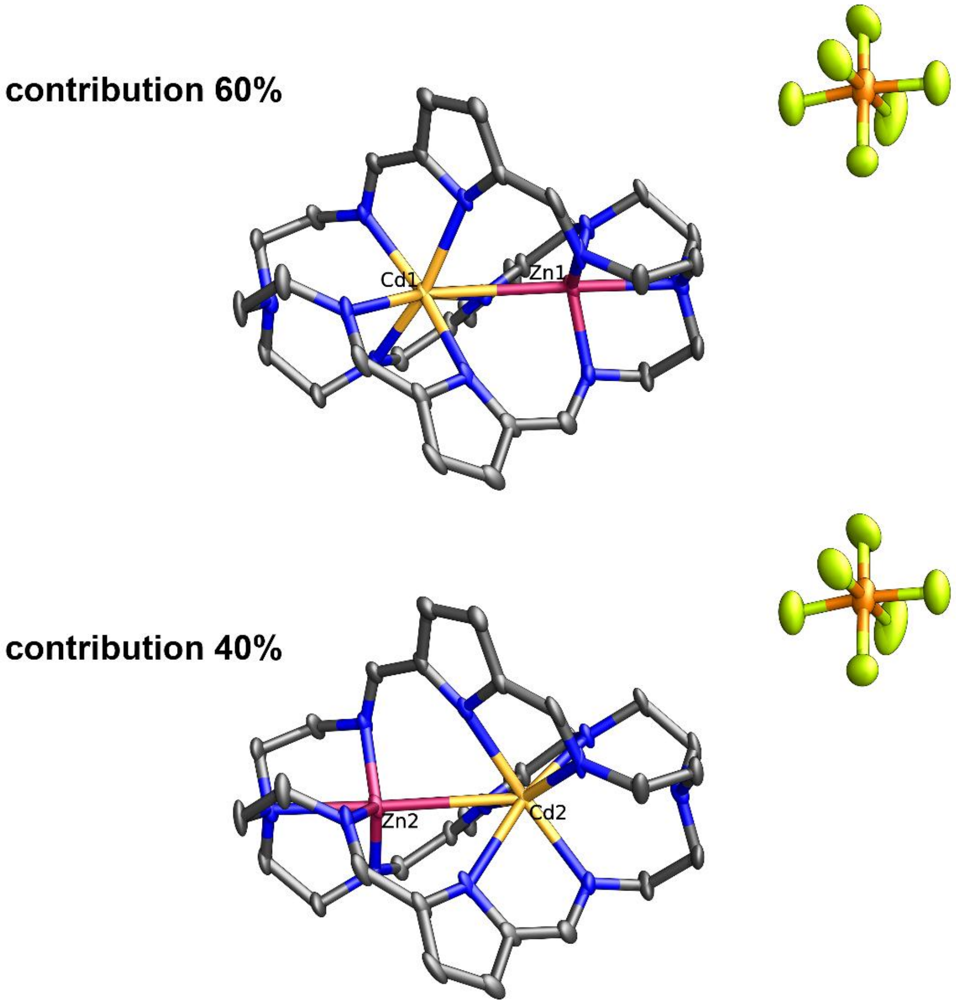

Figure S57. The crystal structure of **[3-CdZn]PF$_6$** with two modes of metal coordination present in the crystal. Hydrogen atoms, solvent molecules, and the minor disordered component of the hexafluorophosphate were omitted for clarity.

Computational results

Table S2. Differences in relative energies between the conformers of the **3-Zn₂**, **3-Cd₂**, and **3-Hg₂** cages in kcal/mol and RMSD values between conformer structures and crystallographic structures.

	ΔE (kcal/mol)			$RMSD_{cryst}$		
Conformer	**3-Zn_2**	**3-Cd_2**	**3-Hg_2**	**3-Zn_2**	**3-Cd_2**	**3-Hg_2**
1	0	0	0	0.25	0.24	0.44
2	5.6	6.4	6.6	0.56	0.59	0.47
3	10.0	11.3	13.0	0.90	0.65	0.69
4	-	-	14.2	-	-	0.87

Table S3. Root-mean-square deviation values between the crystallographic structures and the geometries optimized with different basis set sizes.

	**RMSD**			
**Cage**	**def2-SVP**	**def2-TZVP**	**def2-TZVPP**	**def2-QZVP**
**3-Zn_2**	0.28	0.23	0.23	0.23
**3-Cd_2**	0.34	0.31	0.31	0.31
**3-Hg_2**	0.66	0.51	0.51	0.51
**3-Hg_2^{OTf}**	0.63	0.37	0.37	0.37

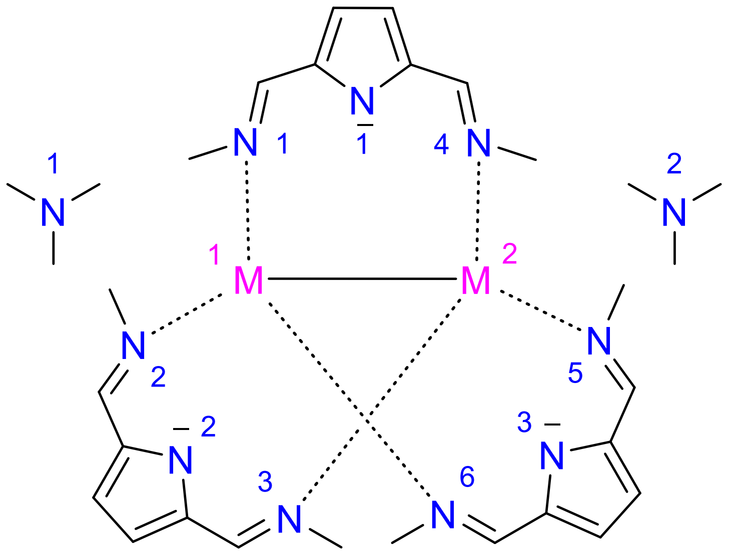

Figure S58. Schematic representation of the cage's structure and ligand indices.

Table S4. Mayer, Wiberg, fuzzy, and Mulliken bond indexes for **3-Zn₂**.

Atom		Mayer	Wiberg	fuzzy	Mulliken
1(Zn)	2(Zn)	0.11	0.13	0.13	-1.32
1(Zn)	N_{imine}(1)	0.51	0.52	0.81	0.38
1(Zn)	N_{amine}(1)	0.17	0.09	0.14	0.22
1(Zn)	N_{imine}(2)	0.40	0.48	0.75	0.21
1(Zn)	N_{imine}(3)	0.05	-	-	0.14
1(Zn)	N_{imine}(4)	0.05	-	-	0.13
1(Zn)	N_{imine}(5)	-	-	-	0.06
1(Zn)	N_{imine}(6)	0.33	0.52	0.79	-
1(Zn)	$N_{pyrrolide}$(1)	0.45	0.44	0.63	0.43
1(Zn)	$N_{pyrrolide}$(2)	0.22	0.36	0.48	0.11
1(Zn)	$N_{pyrrolide}$(3)	-	0.12	0.12	0.06
2(Zn)	N_{imine}(2)	0.05	-	-	0.14
2(Zn)	N_{imine}(3)	0.40	0.48	0.75	0.21
2(Zn)	N_{amine}(2)	0.17	0.09	0.14	0.22
2(Zn)	N_{imine}(4)	0.33	0.52	0.79	-
2(Zn)	N_{imine}(5)	0.51	0.52	0.81	0.39
2(Zn)	N_{imine}(6)	0.05	-	-	0.13

2(Zn)	$N_{pyrrolide}$(1)	-	0.12	0.12	-
2(Zn)	$N_{pyrrolide}$(2)	0.22	0.36	0.49	0.12
2(Zn)	$N_{pyrrolide}$(3)	0.45	0.44	0.63	0.43

Table S5. Mayer, Wiberg, fuzzy, and Mulliken bond indexes for **3-Cd$_2$**.

Atom		Mayer	Wiberg	fuzzy	Mulliken
1(Cd)	2(Cd)	0.06	0.11	0.18	-0.35
1(Cd)	N_{imine}(1)	0.35	0.41	0.83	0.26
1(Cd)	N_{amine}(1)	0.15	0.09	0.27	0.25
1(Cd)	N_{imine}(2)	0.24	0.38	0.76	0.10
1(Cd)	N_{imine}(3)	-	-	-	0.06
1(Cd)	N_{imine}(4)	-	-	-	0.08
1(Cd)	N_{imine}(5)	-	-	-	-
1(Cd)	N_{imine}(6)	0.20	0.41	0.82	-0.06
1(Cd)	$N_{pyrrolide}$(1)	0.30	0.38	0.69	0.24
1(Cd)	$N_{pyrrolide}$(2)	0.13	0.27	0.48	-
1(Cd)	$N_{pyrrolide}$(3)	-	0.06	0.08	-
2(Cd)	N_{imine}(2)	-	-	-	0.06
2(Cd)	N_{imine}(3)	0.24	0.38	0.75	0.11
2(Cd)	N_{amine}(2)	0.14	0.09	0.27	0.24
2(Cd)	N_{imine}(4)	0.20	0.41	0.82	-0.06
2(Cd)	N_{imine}(5)	0.35	0.41	0.83	0.26
2(Cd)	N_{imine}(6)	-	-	-	0.08
2(Cd)	$N_{pyrrolide}$(1)	-	0.06	0.08	-
2(Cd)	$N_{pyrrolide}$(2)	0.13	0.28	0.48	-
2(Cd)	$N_{pyrrolide}$(3)	0.30	0.38	0.69	0.24

Table S6. Mayer, Wiberg, fuzzy, and Mulliken bond indexes for **3-Hg$_2$**.

Atom		Mayer	Wiberg	fuzzy	Mulliken
1(Hg)	2(Hg)	0.09	0.18	0.20	0.05
1(Hg)	N_{imine}(1)	0.38	0.53	0.86	0.17

1(Hg)	$N_{amine}(1)$	0.12	0.09	0.18	0.17
1(Hg)	$N_{imine}(2)$	0.25	0.36	0.59	0.17
1(Hg)	$N_{imine}(3)$	-	-	-	-
1(Hg)	$N_{imine}(4)$	-	-	-	-
1(Hg)	$N_{imine}(5)$	-	-	-	-
1(Hg)	$N_{imine}(6)$	0.31	0.46	0.76	0.13
1(Hg)	$N_{pyrrolide}(1)$	0.10	0.14	0.21	0.08
1(Hg)	$N_{pyrrolide}(2)$	0.43	0.54	0.80	0.23
1(Hg)	$N_{pyrrolide}(3)$	-	0.08	0.08	-
2(Hg)	$N_{imine}(2)$	-	-	-	-
2(Hg)	$N_{imine}(3)$	0.31	0.46	0.75	0.13
2(Hg)	$N_{amine}(2)$	0.12	0.09	0.19	0.18
2(Hg)	$N_{imine}(4)$	0.38	0.53	0.85	0.17
2(Hg)	$N_{imine}(5)$	0.25	0.36	0.60	0.17
2(Hg)	$N_{imine}(6)$	-	-	-	-
2(Hg)	$N_{pyrrolide}(1)$	0.09	0.13	0.17	0.07
2(Hg)	$N_{pyrrolide}(2)$	-	0.08	0.08	-
2(Hg)	$N_{pyrrolide}(3)$	0.43	0.54	0.80	0.23

Table S7. Mayer, Wiberg, fuzzy, and Mulliken bond indexes for **3-Hg$_2^{OTf}$**.

**Atom**		**Mayer**	**Wiberg**	**fuzzy**	**Mulliken**
1(Hg)	2(Hg)	0.10	0.22	0.26	0.05
1(Hg)	$N_{imine}(1)$	0.43	0.55	0.87	0.22
1(Hg)	$N_{amine}(1)$	0.19	0.16	0.28	0.26
1(Hg)	$N_{imine}(2)$	0.27	0.38	0.65	0.16
1(Hg)	$N_{imine}(3)$	-	-	-	-
1(Hg)	$N_{imine}(4)$	-	-	-	-
1(Hg)	$N_{imine}(5)$	-	-	-	-
1(Hg)	$N_{imine}(6)$	0.32	0.45	0.74	0.15
1(Hg)	$N_{pyrrolide}(1)$	-	0.06	0.06	-
1(Hg)	$N_{pyrrolide}(2)$	0.39	0.50	0.75	0.21
1(Hg)	$N_{pyrrolide}(3)$	-	0.08	0.09	-

2(Hg)	N_{imine}(2)	-	-	-	-
2(Hg)	N_{imine}(3)	0.27	0.44	0.71	0.09
2(Hg)	N_{amine}(2)	-	-	0.07	0.07
2(Hg)	N_{imine}(4)	0.32	0.45	0.73	0.16
2(Hg)	N_{imine}(5)	0.19	0.26	0.40	0.17
2(Hg)	N_{imine}(6)	-	-	-	-
2(Hg)	$N_{pyrrolide}$(1)	0.31	0.41	0.61	0.21
2(Hg)	$N_{pyrrolide}$(2)	-	0.07	0.07	-
2(Hg)	$N_{pyrrolide}$(3)	0.41	0.54	0.80	0.20

Table S8. Selected Mulliken partial charges (in |e|) for homobimetallic cages.

**Atom**	**Mulliken partial charge**			
	**3-Zn$_2$**	**3-Cd$_2$**	**3-Hg$_2$**	**3-Hg$_2^{OTf}$**
M(1)	0.44	0.96	0.86	0.79
M(2)	0.44	0.96	0.86	0.87
N_{imine}(1)	-0.13	-0.25	-0.32	-0.32
N_{amine}(1)	-0.09	-0.16	-0.14	-0.16
N_{imine}(2)	-0.12	-0.25	-0.29	-0.28
N_{imine}(3)	-0.12	-0.25	-0.26	-0.25
N_{amine}(2)	-0.09	-0.16	-0.14	-0.11
N_{imine}(4)	-0.08	-0.26	-0.32	-0.32
N_{imine}(5)	-0.13	-0.25	-0.29	-0.26
N_{imine}(6)	-0.08	-0.26	-0.26	-0.25
$N_{pyrrolide}$(1)	-0.20	-0.36	-0.35	-0.37
$N_{pyrrolide}$(2)	-0.27	-0.50	-0.36	-0.34
$N_{pyrrolide}$(3)	-0.20	-0.36	-0.36	-0.38

Table S9. QTAIM parameters (in a.u.) calculated for homobimetallic cages.

	**Cage**		
**Parameter**	**3-Zn$_2$**	**3-Cd$_2$**	**3-Hg$_2$**
CP	-	-	+

$\lvert\nabla\rho\rvert$	0.0049	0.0022	8.5×10^{-17}
$\rho(r)$	0.016	0.017	0.021
RDG	0.20	0.084	2.3×10^{-15}
$\nabla^2\rho$	0.033	0.051	0.064
$\text{sign}(\lambda_2)\cdot\rho$	+	-	-

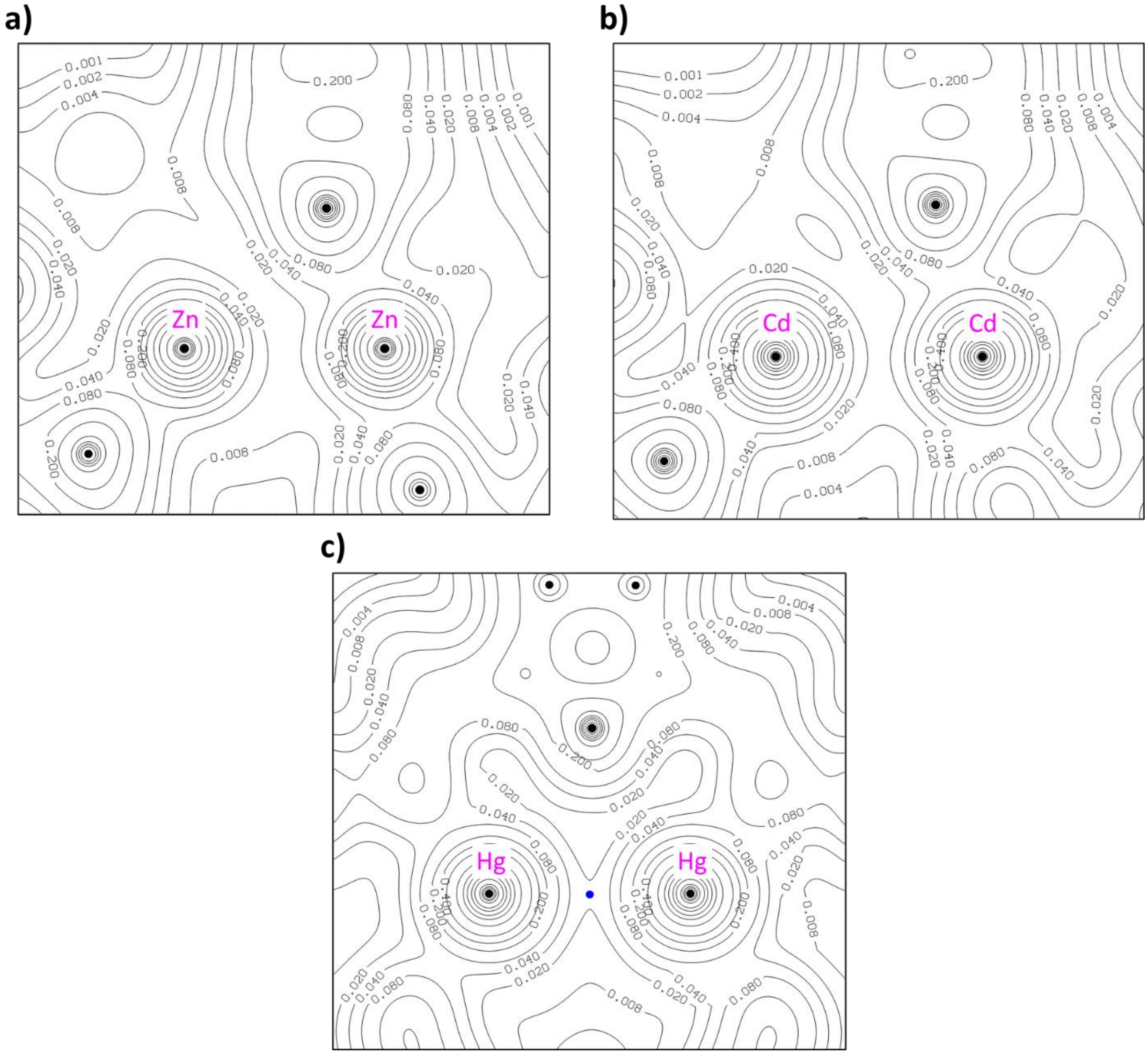

Figure S59. Electron density maps of a) **3-Zn$_2$**, b) **3-Cd$_2$,** and c) **3-Hg$_2$** cages.

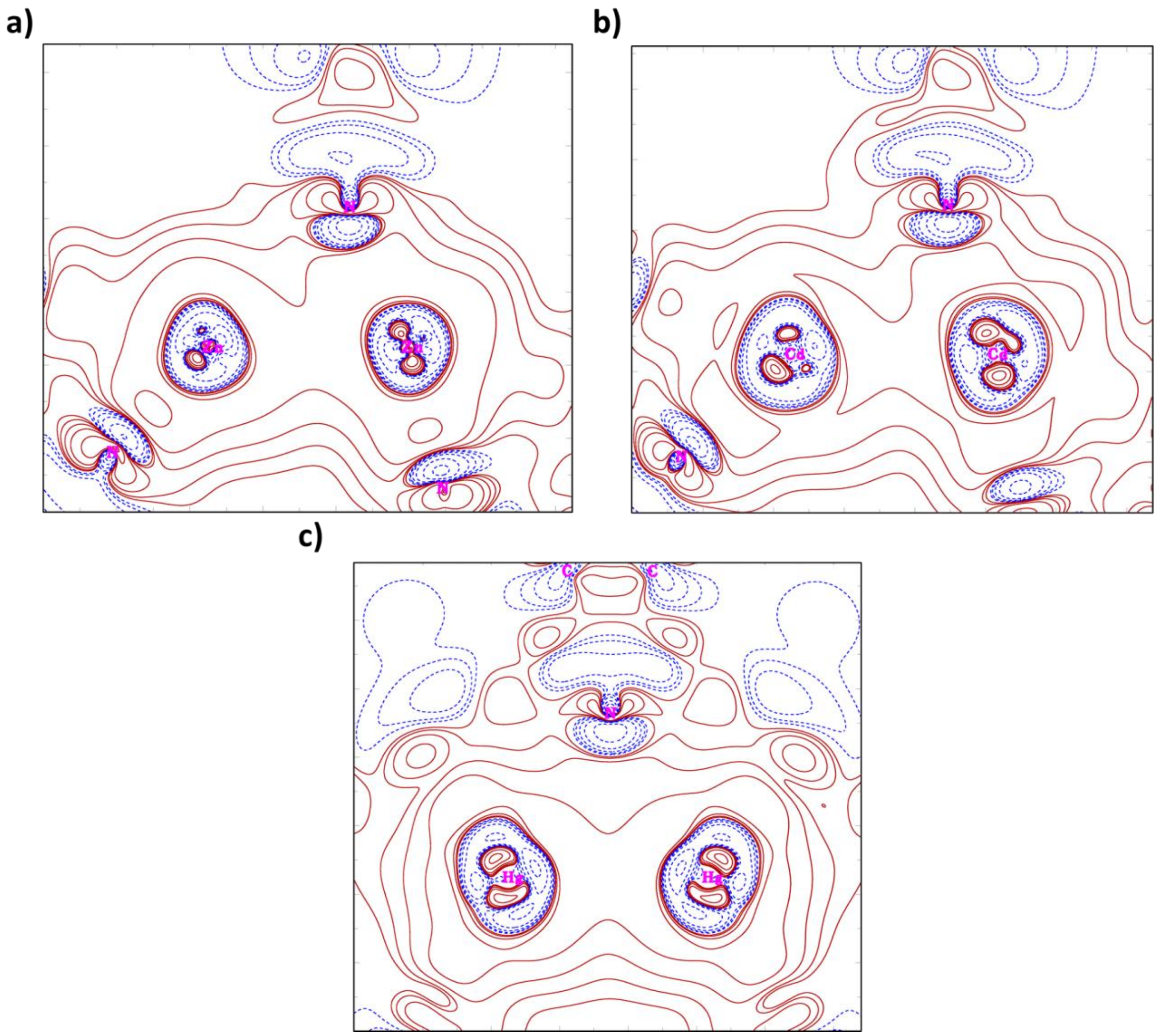

Figure S60. Electron density difference maps of a) **3-Zn$_2$**, b) **3-Cd$_2$,** and c) **3-Hg$_2$** cages.

Table S10. Differences in relative energies between the conformers of **3-CdZn** and **3-CdHg** cages in kcal/mol and RMSD values between conformers structures and crystallographic structures.

	**ΔE (kcal/mol)**		**RMSD$_{cryst}$**	
**Conformer**	**3-CdZn**	**3-CdHg**	**3-CdZn**	**3-CdHg**
1	0	0	0.46	-
2	5.9	6.2	0.71	-
3	11.3	11.4	1.15	-
4	-	14.8	-	-

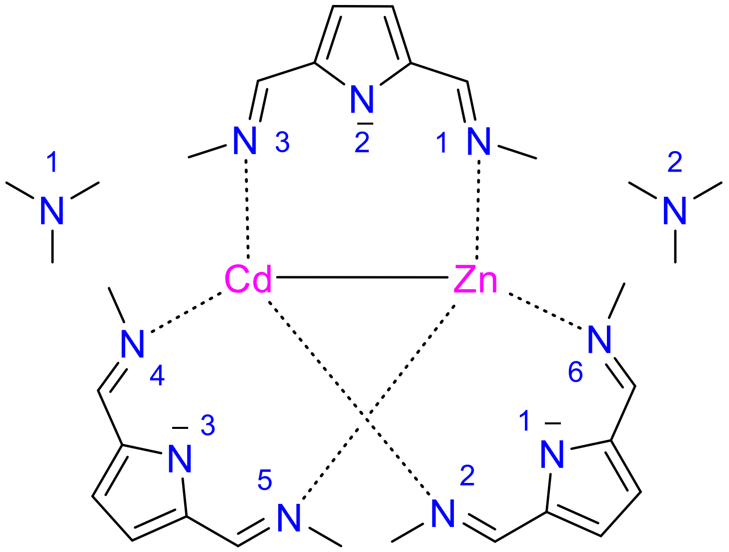

Figure S61. Schematic representation of the **3-CdZn** cage structure and ligand indices

Table S11. Mayer, Wiberg, fuzzy, and Mulliken bond indexes for **3-CdZn**.

Atoms		Mayer	Wiberg	fuzzy	Mulliken
Cd	Zn	0.06	0.12	0.13	-0.86
Cd	$N_{imine}(1)$	-	-	-	0.09
Cd	$N_{pyrrolide}(1)$	-	-	-	-
Cd	$N_{imine}(2)$	0.06	0.39	0.80	-0.37
Cd	$N_{imine}(3)$	0.24	0.36	0.72	0.12
Cd	$N_{imine}(4)$	0.34	0.41	0.82	0.18
Cd	$N_{imine}(5)$	-	-	-	-
Cd	$N_{pyrrolide}(2)$	0.18	0.35	0.65	-
Cd	$N_{pyrrolide}(3)$	0.35	0.34	0.64	0.37
Cd	$N_{imine}(6)$	0.10	0.07	0.14	0.08
Cd	$N_{amine}(1)$	0.13	0.08	0.23	0.22
Cd	$N_{amine}(2)$	-	-	-	-
Zn	$N_{imine}(1)$	0.41	0.52	0.78	0.17
Zn	$N_{pyrrolide}(1)$	0.53	0.53	0.81	0.44
Zn	$N_{imine}(2)$	0.10	-	-	0.20

Zn	$N_{imine}(3)$	-	-	-	0.12
Zn	$N_{imine}(4)$	-	-	-	-
Zn	$N_{imine}(5)$	0.51	0.55	0.83	0.35
Zn	$N_{pyrrolide}(2)$	0.11	0.23	0.24	0.06
Zn	$N_{pyrrolide}(3)$	-	0.09	0.07	-0.12
Zn	$N_{imine}(6)$	0.41	0.50	0.67	0.29
Zn	$N_{amine}(1)$	-	-	-	-
Zn	$N_{amine}(2)$	0.21	0.11	0.19	0.31

Table S12. Selected Mulliken partial charges (in |e|) for **3-CdZn** cage.

**Atom**	**Mulliken partial charge**
Cd	1.02
Zn	0.37
$N_{imine}(1)$	-0.10
$N_{pyrrolide}(1)$	-0.12
$N_{imine}(2)$	-0.27
$N_{imine}(3)$	-0.26
$N_{imine}(4)$	-0.26
$N_{imine}(5)$	-0.11
$N_{pyrrolide}(2)$	-0.41
$N_{pyrrolide}(3)$	-0.36
$N_{imine}(6)$	-0.21
$N_{amine}(1)$	-0.15
$N_{amine}(2)$	-0.09

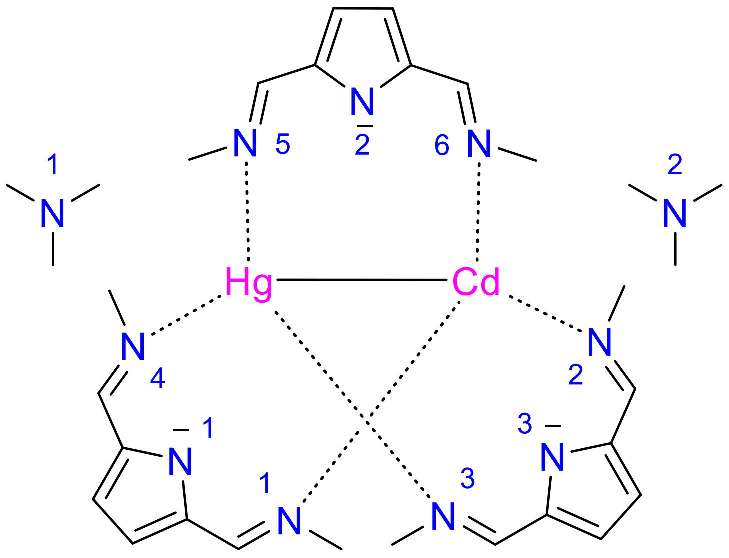

Figure S62. Schematic representation of the **3-CdHg** cage structure and ligand indices.

Table S13. Mayer, Wiberg, fuzzy, and Mulliken bond indexes for the **3-CdHg** cage.

**Atoms**		**Mayer**	**Wiberg**	**fuzzy**	**Mulliken**
Hg	Cd	0.06	0.15	0.21	-0.10
Hg	$N_{imine}(1)$	-	-	-	0.08
Hg	$N_{imine}(2)$	-	-	-	-
Hg	$N_{imine}(3)$	0.32	0.47	0.78	0.14
Hg	$N_{imine}(4)$	0.27	0.40	0.66	0.14
Hg	$N_{imine}(5)$	0.37	0.49	0.81	0.19
Hg	$N_{imine}(6)$	-	-	-	-
Hg	$N_{pyrrolide}(1)$	0.44	0.53	0.78	0.28
Hg	$N_{pyrrolide}(2)$	0.10	0.15	0.17	0.06
Hg	$N_{pyrrolide}(3)$	-	0.07	0.07	-
Hg	$N_{amine}(1)$	0.14	0.12	0.23	0.20
Hg	$N_{amine}(2)$	-	-	-	-
Cd	$N_{imine}(1)$	0.17	0.40	0.80	-0.11
Cd	$N_{imine}(2)$	0.34	0.41	0.83	0.22
Cd	$N_{imine}(3)$	-	-	-	-

Cd	$N_{imine}(4)$	-	-	-	-
Cd	$N_{imine}(5)$	-	-	-	-
Cd	$N_{imine}(6)$	0.23	0.34	0.69	0.18
Cd	$N_{pyrrolide}(1)$	-	-	0.07	
Cd	$N_{pyrrolide}(2)$	0.20	0.39	0.70	0.07
Cd	$N_{pyrrolide}(3)$	0.25	0.32	0.60	0.22
Cd	$N_{amine}(1)$	-	-	-	-
Cd	$N_{amine}(2)$	0.12	0.07	0.21	0.19

Table S14. Selected Mulliken partial charges (in |e|) for **3-CdHg**.

Atom	Mulliken partial charge
Hg	0.77
Cd	1.05
$N_{imine}(1)$	-0.26
$N_{imine}(2)$	-0.26
$N_{imine}(3)$	-0.30
$N_{imine}(4)$	-0.29
$N_{imine}(5)$	-0.27
$N_{imine}(6)$	-0.26
$N_{pyrrolide}(1)$	-0.35
$N_{pyrrolide}(2)$	-0.44
$N_{pyrrolide}(3)$	-0.36
$N_{amine}(1)$	-0.15
$N_{amine}(2)$	-0.15

Table S15. QTAIM parameters (in a.u.) calculated for **3-CdZn** and **3-CdHg**.

	Cage	
**Parameter**	**Zn-Cd**	**Hg-Cd**
CP	-	+
$\|\nabla\rho\|$	0.0071	$5,3 \times 10^{-17}$
$\rho(r)$	0.015	0.019

RDG	0.32	1.7 x 10^{-15}
$\nabla^2\rho$	0.040	0.058
sign(λ_2)·ρ	-	-

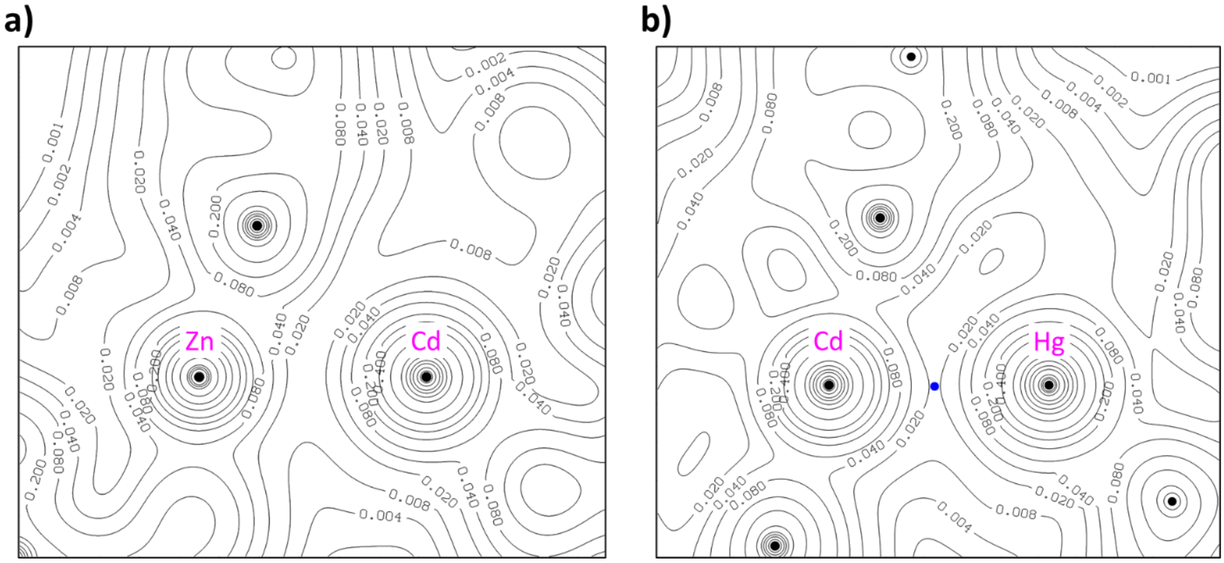

Figure S63. Electron density maps of a) **3-CdZn**, and b) **3-CdHg**.

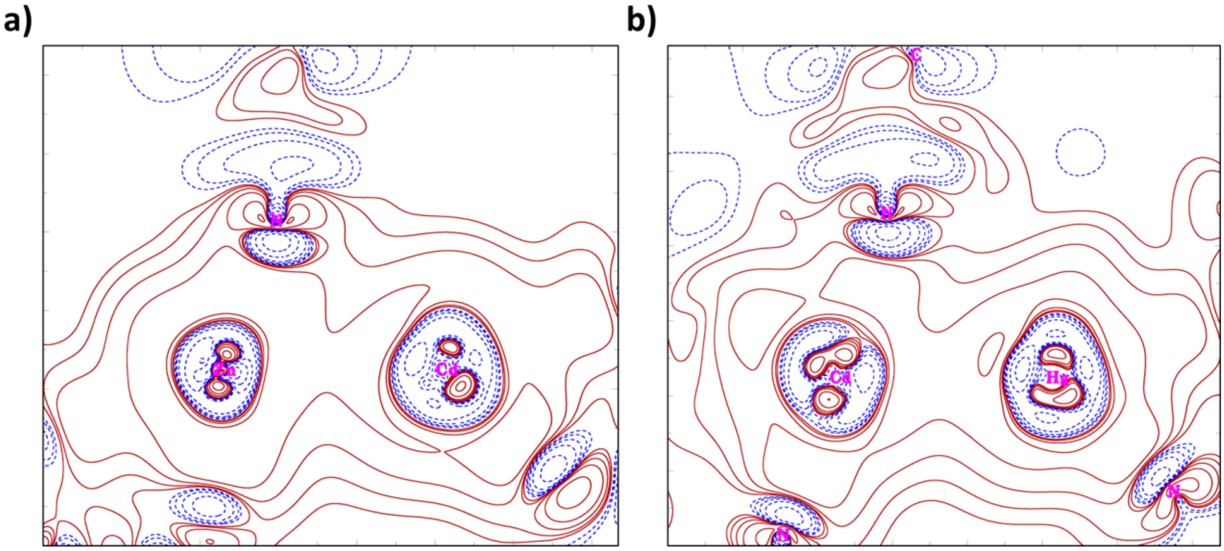

Figure S64. Electron density maps of a) **3-CdZn**, and b) **3-CdHg** cages.

Table S16. QTAIM parameters (in a.u.) calculated for bimetallic cages, with the incorporation of a continuum solvation model (SMD) of acetonitrile. RMSD values represent deviations from the gas-phase geometries.

	**Cage**				
**Parameter**	**3-Zn$_2$**	**3-Cd$_2$**	**3-Hg$_2$**	**3-CdZn**	**3-CdHg**
RMSD	0.12	0.03	0.10	0.11	0.06
CP	-	-	+	-	+
$\|\nabla\rho\|$	0.0057	0.0020	4.8×10^{-17}	0.0051	5.2×10^{-17}
$\rho(r)$	0.013	0.015	0.019	0.012	0.017
RDG	0.30	0.090	1.5×10^{-15}	0.32	2.0×10^{-15}
$\nabla^2\rho$	0.029	0.045	0.058	0.032	0.052
$\mathrm{sign}(\lambda_2)\cdot\rho$	+	-	-	-	-

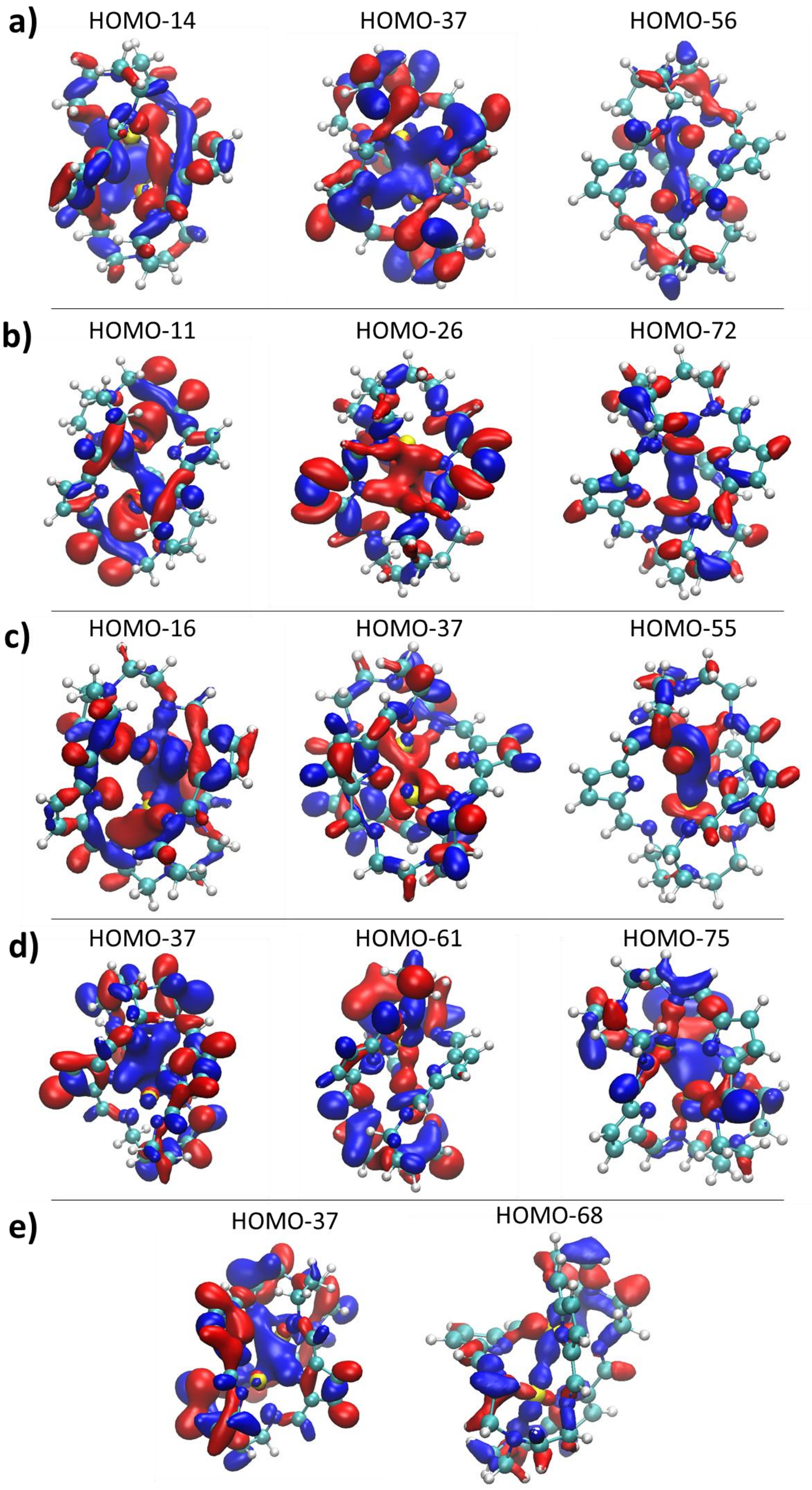

Figure S65 Selected molecular orbitals contributing to metal-metal interactions: a) **3-Zn$_2$**, b) **3-Cd$_2$,** c) **3-Hg$_2$**, d) **3-CdZn**, and e) **3-CdHg**.

In all cages, the molecular orbitals delocalized over both cations arise mainly from the mixing of nitrogen ligand orbitals with metal d orbitals, as well as from the interactions between the d orbitals of the two metal centers. Notably, in **3-Hg$_2$**, an orbital delocalized at the center of the cavity and wrapping around both cations is observed (Figure S65c, HOMO-37). This orbital is composed primarily of Hg s orbitals, with additional contributions from the d_{-1} and d_{+2} orbitals of the first Hg center and the d_{-2} orbital of the second. In contrast, for **3-Zn$_2$** and **3-Cd$_2$**, delocalized molecular orbitals are present; they arise mainly from ligand orbitals around the metal centers. These orbitals exhibit a smaller metal s character compared to **3-Hg$_2$**, and show negligible or reduced involvement of metal d orbitals for Zn and Cd, respectively. Similarly, in **3-ZnCd** and **3-CdHg**, delocalized molecular orbitals surrounding cations are formed that involve metal s orbital mixing, however, with no d orbitals contributing from Zn and Cd atoms. These observations suggest that the strong mercurophilic interaction in **3-Hg$_2$** arises from a substantial contribution of more diffuse Hg s orbitals combined with effective s-d atomic orbital mixing. Furthermore, the slightly weaker interaction in **3-CdHg** can be attributed to less efficient overlapping of the metal d orbitals.

UV-vis absorption spectra

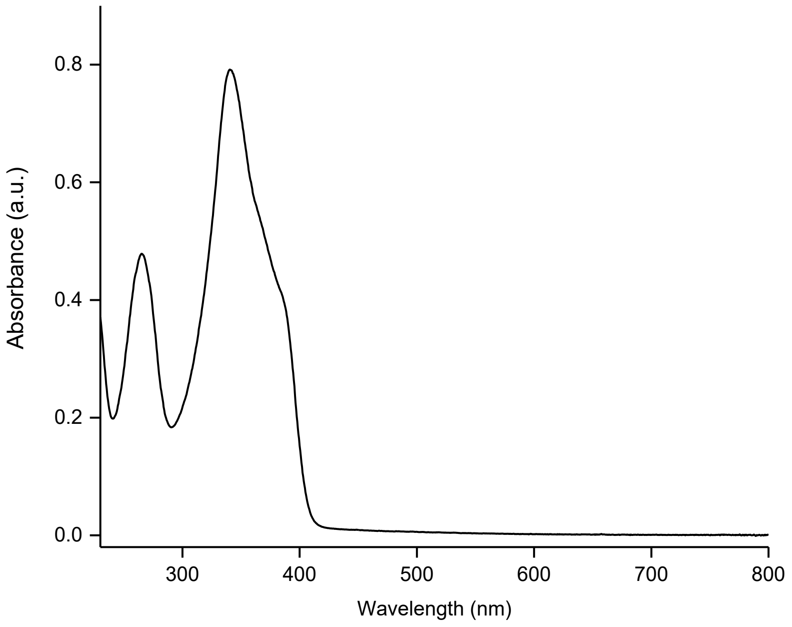

Figure S66. The UV-vis absorption spectrum of **3-Zn$_2$** (MeCN, 298 K).

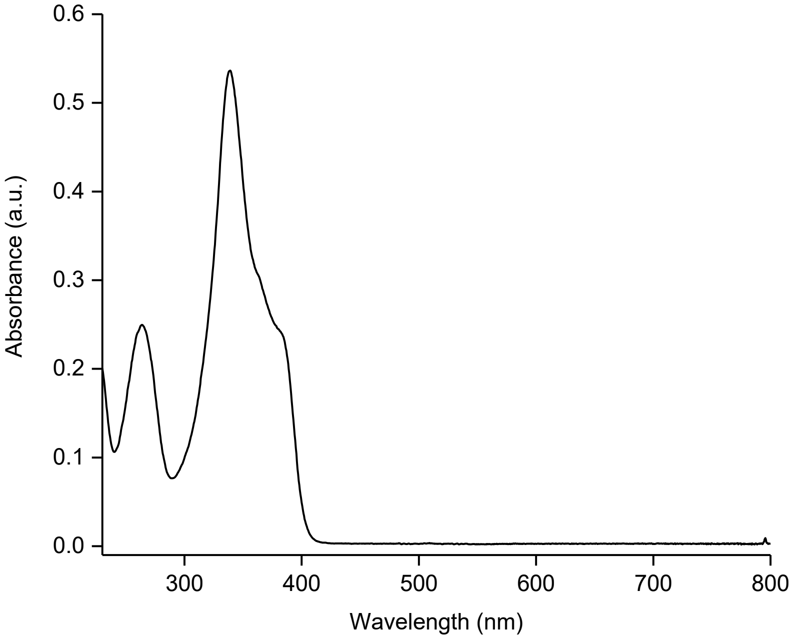

Figure S67. The UV-vis absorption spectrum of **3-Cd$_2$** (MeCN, 298 K).

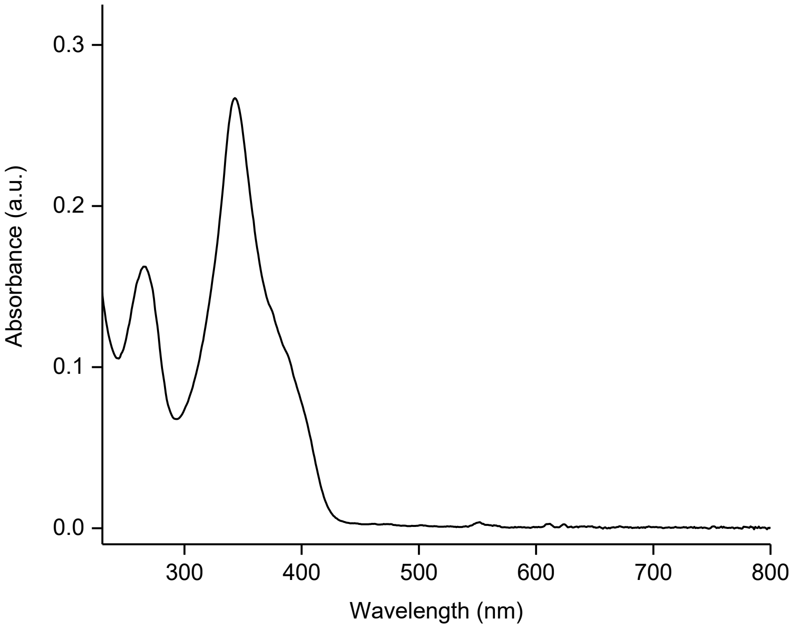

Figure S68. The UV-vis absorption spectrum of **3-Hg$_2$** (MeCN, 298 K).

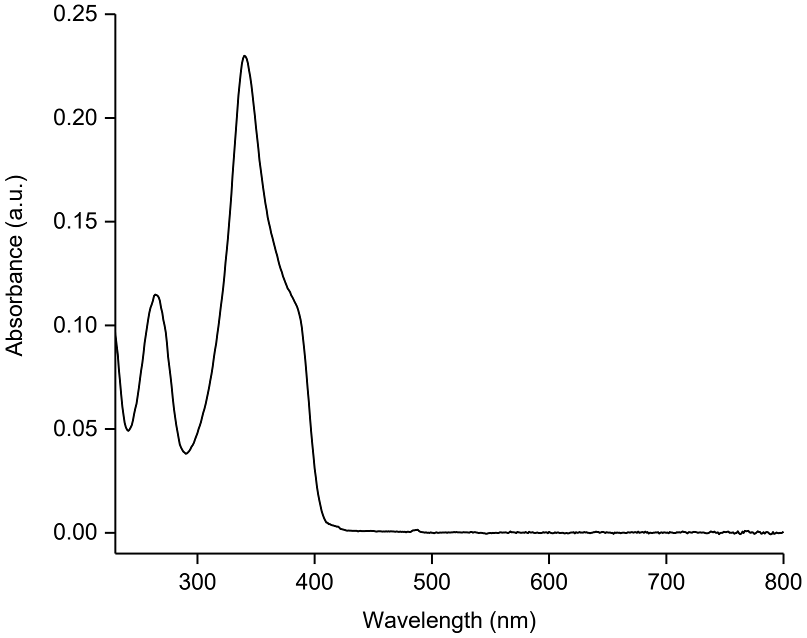

Figure S69. The UV-vis absorption spectrum of **3-ZnCd** (MeCN, 298 K).

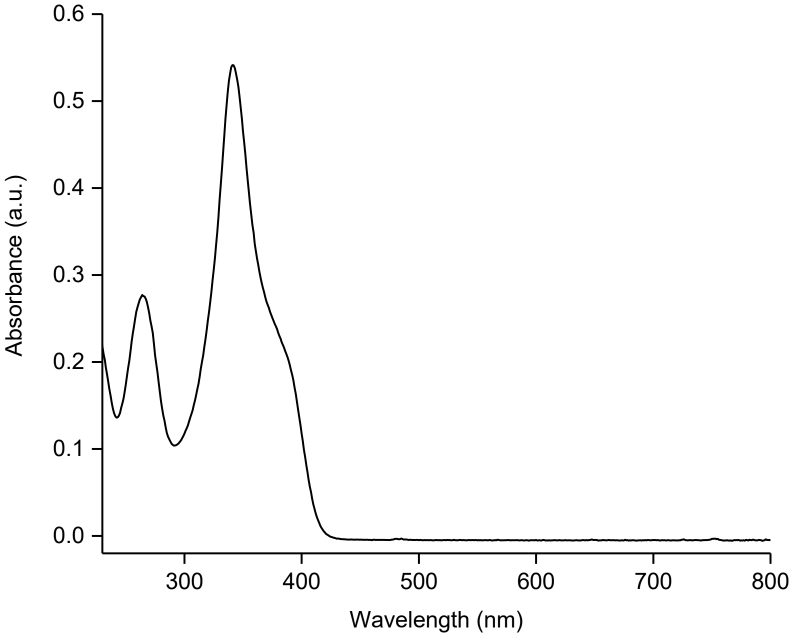

Figure S70. The UV-vis absorption spectrum of **3-HgCd** (MeCN, 298 K).

Cartesian Coordinates

Table S17. DFT-optimized Cartesian coordinates of **3-Zn$_2$**.

Zn	5.99450	3.56470	7.09590
Zn	6.58320	0.97750	5.95200
C	5.03280	1.82700	3.58750
H	4.73700	1.79210	2.53380
C	4.11730	2.49480	4.47770
C	2.76490	2.72750	4.17760
H	2.27540	2.52000	3.23620
C	2.19520	3.24960	5.33510
H	1.17200	3.56280	5.48680
C	3.23960	3.33210	6.26460
C	3.24560	4.04110	7.51820
H	2.29860	4.41840	7.91310
N	4.35400	4.28780	8.10200
C	4.42200	5.20710	9.21990
H	5.08730	4.78720	9.97950
H	3.43770	5.35970	9.67740
C	4.97340	6.54260	8.70380
H	4.25140	6.95240	7.99190
H	5.04520	7.25790	9.53640
N	6.24030	6.36320	8.01990
C	7.41390	6.29810	8.86990
H	7.97400	7.24520	8.88080
H	7.08320	6.11580	9.89600
C	8.35660	5.16050	8.46550
H	8.79150	5.35450	7.48250
H	9.18200	5.08980	9.18320
N	7.60720	3.92100	8.40010
C	7.80390	2.99520	9.24960
H	8.55500	3.08770	10.03870
C	6.96420	1.82200	9.22560
C	6.60770	1.03900	10.31970
H	7.04750	1.07980	11.30620
C	5.53910	0.24190	9.89240
H	5.00460	-0.50570	10.46120
C	5.33080	0.56420	8.55450
C	4.54750	-0.18830	7.60480
H	3.72700	-0.80860	7.97510
N	4.86940	-0.16800	6.37450
C	4.17420	-0.99980	5.41170
H	3.84260	-0.36890	4.58390
H	3.28210	-1.45950	5.85260
C	5.12410	-2.09120	4.90940
H	5.34700	-2.75840	5.74650
H	4.60850	-2.69720	4.14940
N	6.37540	-1.54420	4.42030
C	6.36620	-1.01340	3.06930
H	5.33950	-1.04150	2.69100
H	6.96920	-1.62470	2.38420
C	6.85360	0.44160	3.01170
H	7.91240	0.50420	3.27790
H	6.73910	0.82930	1.99300
N	6.09480	1.23500	3.96980
C	7.57780	-2.23110	4.85320
H	8.38300	-1.99280	4.15240
H	7.45970	-3.32470	4.84760
C	8.01470	-1.76860	6.24920
H	7.26000	-2.03630	6.99390
H	8.95560	-2.25670	6.52830
N	8.14970	-0.32630	6.22190
C	9.29720	0.22660	6.13480
H	10.21740	-0.36020	6.19920
C	9.39310	1.63550	5.85230
C	10.51780	2.34240	5.40830
H	11.53410	1.97690	5.36940
C	10.04040	3.58160	4.99170
H	10.60900	4.40410	4.58060
C	8.65660	3.56890	5.23290
C	7.80350	4.71830	5.06770
H	8.19760	5.52120	4.43610
N	6.68500	4.86670	5.66070
C	5.98800	6.14230	5.56120
H	4.91220	5.94740	5.58520
H	6.21710	6.65020	4.61750
C	6.40230	7.03250	6.74170
H	7.45800	7.29270	6.61570
H	5.84030	7.97530	6.69890
N	4.40990	2.86200	5.75230
N	6.19380	1.53860	8.12980
N	8.25850	2.38190	5.75980

Table S18. DFT-optimized Cartesian coordinates of **3-Cd$_2$**.

Cd	5.89690	3.61930	7.10510
Cd	6.68090	0.92630	5.93960
C	4.69300	1.73980	3.60280
H	4.27740	1.58570	2.60010

C	3.80990	2.42600	4.51000
C	2.44580	2.61950	4.23240
H	1.93120	2.31900	3.33030
C	1.91020	3.26290	5.34230
H	0.89040	3.58750	5.49300
C	2.98090	3.44730	6.22600
C	2.97950	4.27790	7.40750
H	2.03050	4.73370	7.70650
N	4.06180	4.53870	8.02760
C	4.14230	5.58070	9.02690
H	4.65670	5.18740	9.90850
H	3.14940	5.92570	9.33940
C	4.92490	6.75440	8.42400
H	4.35280	7.12970	7.57140
H	4.99060	7.57290	9.15620
N	6.24120	6.34370	7.94370
C	7.25620	6.28660	8.99050
H	7.77960	7.24740	9.10750
H	6.75710	6.07820	9.94020
C	8.27900	5.17430	8.74320
H	8.83610	5.35980	7.82080
H	9.00120	5.15260	9.56800
N	7.57990	3.91380	8.61370
C	7.71890	2.98550	9.47080
H	8.40930	3.08600	10.31380
C	6.90310	1.79420	9.40970
C	6.55560	0.99910	10.49980
H	6.97190	1.06210	11.49530
C	5.53580	0.15340	10.05190
H	5.01840	-0.61190	10.61320
C	5.34680	0.45740	8.70550
C	4.58750	-0.33810	7.76790
H	3.81720	-1.00200	8.17210
N	4.86140	-0.31840	6.52700
C	4.21540	-1.21710	5.59460
H	3.77210	-0.62390	4.79020
H	3.40850	-1.78450	6.07330
C	5.26030	-2.18740	5.03670
H	5.64620	-2.78050	5.86960
H	4.77600	-2.88870	4.34060
N	6.38070	-1.48950	4.41460
C	6.10510	-1.01180	3.06080
H	5.03010	-1.08700	2.87400
H	6.59870	-1.63560	2.30370
C	6.51440	0.45230	2.86890
H	7.59560	0.56880	2.98630
H	6.25040	0.77320	1.85460
N	5.84930	1.27460	3.87540
C	7.65810	-2.18180	4.55990
H	8.33240	-1.82000	3.77910
H	7.55560	-3.26850	4.42140
C	8.31780	-1.89140	5.91380
H	7.69750	-2.26680	6.73290
H	9.28840	-2.39930	5.96360
N	8.45170	-0.45780	6.04650
C	9.57910	0.12800	5.94740
H	10.51210	-0.44210	5.90050
C	9.66190	1.56160	5.79440
C	10.80920	2.29390	5.46460
H	11.82180	1.91790	5.42620
C	10.36280	3.57560	5.16350
H	10.95410	4.42510	4.85070
C	8.96970	3.56050	5.34810
C	8.15500	4.73510	5.17450
H	8.66630	5.56810	4.67780
N	6.95510	4.89310	5.57800
C	6.36130	6.22210	5.46420
H	5.28040	6.10870	5.34120
H	6.74530	6.75580	4.58730
C	6.67930	7.02780	6.72820
H	7.76330	7.16560	6.77450
H	6.23940	8.03070	6.64790
N	4.13780	2.92650	5.73090
N	6.17430	1.46810	8.29860
N	8.53980	2.33140	5.73950

Table S19. DFT-optimized Cartesian coordinates of **3-Hg$_2$**.

Hg	5.99840	3.44580	7.42460
Hg	6.59300	1.18880	5.40740
C	4.57580	1.66380	3.07970
H	4.14140	1.46590	2.09450
C	3.81530	2.54150	3.91840
C	2.54890	3.04890	3.54620
H	2.04950	2.89430	2.59980
C	2.09710	3.76190	4.63900
H	1.16520	4.29980	4.74380
C	3.11900	3.65790	5.61060
C	3.07670	4.35260	6.86310
H	2.16260	4.92390	7.05400
N	4.01970	4.39790	7.72680
C	3.92590	5.33430	8.83820
H	4.36600	4.87330	9.72650
H	2.87950	5.57040	9.06210
C	4.67490	6.61860	8.47230
H	4.17480	7.06160	7.60630
H	4.58610	7.34310	9.29380
N	6.06700	6.35970	8.12840
C	6.99580	6.47960	9.24330
H	7.36540	7.50960	9.36750

H	6.45770	6.22050	10.15910
C	8.18750	5.52750	9.11210
H	8.77120	5.77340	8.21860
H	8.84950	5.64610	9.97920
N	7.70610	4.17500	8.97740
C	8.12760	3.21580	9.69320
H	8.89920	3.35240	10.45750
C	7.54910	1.89500	9.53540
C	7.66680	0.78580	10.37420
H	8.30950	0.70300	11.23900
C	6.76450	-0.16400	9.88930
H	6.57130	-1.15140	10.28460
C	6.16890	0.40110	8.75590
C	5.25810	-0.28180	7.87520
H	4.70780	-1.11690	8.32200
N	5.08980	-0.01030	6.64030
C	4.19830	-0.86400	5.86050
H	3.73940	-0.25450	5.07920
H	3.39320	-1.25930	6.48940
C	4.98450	-2.03050	5.25500
H	5.39970	-2.62100	6.07590
H	4.29120	-2.69150	4.71500
N	6.07630	-1.57870	4.40420
C	5.70240	-1.33350	3.01700
H	4.61140	-1.34650	2.93880
H	6.07620	-2.12180	2.34910
C	6.19020	0.03120	2.52300
H	7.28240	0.07690	2.53800
H	5.85920	0.18730	1.49000
N	5.66380	1.06990	3.39840
C	7.32810	-2.30340	4.57250
H	7.97600	-2.05600	3.72730
H	7.18190	-3.39490	4.55820
C	8.05980	-1.90770	5.85790
H	7.45210	-2.16240	6.73260
H	8.99820	-2.47120	5.93450
N	8.28010	-0.48290	5.86040
C	9.41440	0.04270	6.07790
H	10.30390	-0.55670	6.29590
C	9.56440	1.48300	5.99410
C	10.73800	2.23570	5.93270
H	11.74480	1.86180	6.05240
C	10.34370	3.54550	5.64930
H	10.97810	4.41150	5.52200
C	8.94560	3.53170	5.58840
C	8.11530	4.69570	5.42400
H	8.59720	5.55150	4.93890
N	6.91040	4.80280	5.82870
C	6.24240	6.09150	5.66750
H	5.17280	5.90850	5.54430
H	6.59830	6.60110	4.76560
C	6.51090	6.97800	6.88730
H	7.58830	7.15340	6.94740
H	6.04390	7.96080	6.72900
N	4.16530	2.91320	5.17510
N	6.65230	1.65390	8.54360
N	8.47640	2.27390	5.80240

Table S20. DFT-optimized Cartesian coordinates of **3-Hg$_2^{OTf}$**.

Hg	6.89420	3.74240	7.69720
Hg	6.50940	1.76060	5.47460
C	4.88770	2.19970	2.93630
H	4.43580	2.10170	1.94360
C	4.45600	3.30250	3.74300
C	3.48840	4.25920	3.39830
H	3.01260	4.36990	2.43390
C	3.27810	5.01410	4.54190
H	2.63390	5.87300	4.67150
C	4.13450	4.48240	5.52610
C	4.16490	5.06070	6.84470
H	3.39010	5.80770	7.02110
N	4.99700	4.83710	7.79140
C	4.94410	5.69380	8.97940
H	5.17440	5.07750	9.85480
H	3.94950	6.12870	9.09970
C	5.95000	6.83480	8.82290
H	5.61110	7.46050	7.99870
H	5.93520	7.45950	9.72490
N	7.30800	6.35550	8.53070
C	8.17860	6.28520	9.69410
H	8.65290	7.25420	9.91810
H	7.56210	6.02080	10.55770
C	9.26690	5.21370	9.54750
H	9.92630	5.45720	8.70840
H	9.88050	5.18220	10.45690
N	8.63560	3.94720	9.27820
C	8.86360	2.88010	9.92510
H	9.61470	2.83100	10.72020
C	8.06270	1.70150	9.66010
C	7.97160	0.52420	10.40750
H	8.60100	0.24380	11.24030
C	6.88420	-0.17700	9.88490
H	6.49410	-1.13060	10.21330
C	6.38760	0.59510	8.82480
C	5.27100	0.20700	7.99770
H	4.61920	-0.55200	8.44720
N	5.01150	0.63470	6.82700
C	3.86610	0.05420	6.12700
H	3.56160	0.75150	5.34340

H	3.01810	-0.06620	6.81110
C	4.22610	-1.31040	5.52750
H	4.48550	-1.98700	6.34780
H	3.32760	-1.73260	5.05220
N	5.34400	-1.24660	4.60730
C	5.02230	-0.99840	3.21560
H	3.98420	-0.65990	3.14910
H	5.10000	-1.91270	2.60600
C	5.91080	0.09490	2.61370
H	6.96290	-0.19590	2.67860
H	5.65840	0.23080	1.55390
N	5.71400	1.32010	3.35920
C	6.41000	-2.20360	4.82600
H	7.03200	-2.22540	3.92700
H	6.02940	-3.22850	4.97490
C	7.30590	-1.82530	6.00640
H	6.73320	-1.86490	6.93890
H	8.12040	-2.55890	6.09470
N	7.79680	-0.48090	5.83650
C	9.04030	-0.24030	5.80090
H	9.79220	-1.03300	5.89840
C	9.52490	1.11350	5.60920
C	10.82170	1.53500	5.31860
H	11.69860	0.90600	5.25720
C	10.74440	2.91610	5.10610
H	11.55150	3.59400	4.86590
C	9.40910	3.26920	5.31300
C	8.89270	4.61720	5.35160
H	9.45210	5.36580	4.78080
N	7.87800	4.97530	6.02950
C	7.48830	6.38180	6.05190
H	6.41030	6.46380	5.91820
H	7.96040	6.92550	5.22630
C	7.90870	7.02180	7.37540
H	8.99760	6.95800	7.45920
H	7.64300	8.08630	7.34390
N	4.86080	3.44040	5.03550
N	7.11470	1.73270	8.68930
N	8.67070	2.16930	5.61650
O	4.91760	8.17660	5.97150
O	2.56930	8.27550	5.28520
O	3.15960	8.02290	7.64610
S	3.54260	8.49310	6.32580
F	3.93980	10.93010	5.39180
F	2.38780	10.79700	6.89710
F	4.47250	10.67440	7.47660
C	3.58380	10.32220	6.53070

Table S21. DFT-optimized Cartesian coordinates of **3-CdZn**.

Cd	10.28570	6.41210	12.09550
N	11.00830	9.31070	14.37950
N	7.92420	9.91870	13.92340
N	9.73080	6.67410	9.91420
N	8.41010	5.08770	12.40790
N	12.11870	5.10440	12.21250
N	10.46580	10.38480	11.50350
N	9.44620	6.97850	14.18560
N	12.11600	7.87650	11.70050
N	8.02280	8.35370	11.68200
N	10.20800	3.91680	10.50580
N	9.93760	11.86130	13.84040
C	11.88620	10.46730	14.47090
H	12.41870	10.57460	13.52430
H	12.63610	10.33260	15.25810
C	10.87490	8.54110	15.38800
H	11.44330	8.71440	16.30600
C	7.99140	10.88610	14.99590
H	8.58980	10.46840	15.81080
H	6.99750	11.11900	15.39400
C	9.23510	6.92310	16.45400
H	9.40650	7.14700	17.49750
C	9.84860	11.70280	11.37430
H	8.76350	11.57490	11.32570
H	10.16960	12.20360	10.45440
C	7.71020	7.85520	10.45600
C	8.44340	6.12170	14.52650
C	8.26380	6.07740	15.91010
H	7.54310	5.47360	16.44330
C	14.34290	7.83940	11.18010
H	15.35250	7.47080	11.06550
C	6.82830	8.73320	12.22540
C	13.24890	7.12810	11.69750
C	7.86040	5.23130	13.54460
H	6.97510	4.65630	13.83310
C	9.92600	7.46040	15.36610
C	8.62680	7.19220	9.55530
H	8.27890	7.08840	8.52100
C	6.81640	9.52870	13.42390
H	5.85730	9.83560	13.84800
C	10.47950	5.85610	8.97160
H	11.54560	5.97590	9.18460
H	10.30340	6.17180	7.93660
C	13.84360	9.08400	10.82050
H	14.38220	9.90980	10.37660
C	5.76010	8.46490	11.36690
H	4.71710	8.67880	11.55230
C	12.04090	3.65860	12.15990
H	11.38120	3.30940	12.95860
H	13.02320	3.19210	12.30120

C	12.47710	9.07050	11.15910
C	13.21420	5.70930	11.97040
H	14.15460	5.15660	11.87820
C	7.91770	4.14180	11.43240
H	7.55690	4.69910	10.56250
H	7.07520	3.55710	11.82130
C	11.48350	3.26710	10.78510
H	12.20650	3.59200	10.03190
H	11.41310	2.17170	10.71120
C	10.05590	4.39070	9.13380
H	9.00260	4.31280	8.85030
H	10.61670	3.76780	8.42370
C	9.05220	3.19830	11.02680
H	9.36710	2.64810	11.91730
H	8.66940	2.45550	10.30970
C	6.32990	7.91760	10.21900
H	5.82280	7.58520	9.32400
C	10.23860	12.54760	12.59320
H	11.31630	12.73120	12.54650
H	9.74800	13.52820	12.53760
C	11.04420	11.71220	14.77150
H	10.63390	11.60770	15.77970
H	11.69460	12.59840	14.78600
C	8.64920	12.15680	14.44530
H	7.99400	12.56760	13.67260
H	8.72370	12.91130	15.24240
C	11.62710	10.21460	10.99410
H	12.06730	11.02570	10.40470
Zn	9.55810	9.22300	12.90480

Table S22. DFT-optimized Cartesian coordinates of **3-CdHg**.

Hg	1.43810	0.30430	0.11440
N	-1.98330	0.99960	1.56450
N	-2.96960	-2.05720	-0.30990
N	1.88650	-0.50420	2.19600
N	2.96470	1.85870	-0.78210
N	2.02080	-1.59040	-0.93360
N	-2.49980	1.30560	-1.60470
N	0.45150	2.29740	0.30510
N	-0.27620	-0.27470	-2.13440
N	-0.59910	-2.03260	1.24370
N	4.21870	-0.32740	0.52280
N	-4.44630	0.30810	0.20310
C	-3.15630	0.54710	2.31310
H	-3.19470	1.01710	3.30220
H	-3.07000	-0.53280	2.46080
C	-1.61690	2.21070	1.71450
H	-2.15180	2.84920	2.42720
C	-4.31500	-1.93170	-0.83320
H	-4.82080	-2.90330	-0.88350
H	-4.26710	-1.52010	-1.84500
C	-0.44780	4.27950	0.94230
H	-1.11630	4.98980	1.40880
C	-3.63580	2.13180	-1.26300
H	-3.93830	2.77600	-2.09810
H	-3.35020	2.78380	-0.43210
C	0.28310	-2.34560	2.23420
C	1.21420	3.31390	-0.18400
C	0.67780	4.55410	0.16620
H	1.08450	5.52200	-0.08960
C	0.45110	-0.99530	-4.16830
H	1.01690	-1.53890	-4.91200
C	-1.43050	-3.10520	1.16790
C	0.57440	-1.11930	-2.77950
C	2.51720	3.04670	-0.75780
H	3.11410	3.89850	-1.09780
C	-0.55860	2.88420	1.00430
C	1.39220	-1.56460	2.70930
H	1.87060	-1.98330	3.60250
C	-2.63720	-3.07710	0.37840
H	-3.30240	-3.94320	0.45310
C	3.13730	0.01370	2.73800
H	3.24240	-0.23850	3.79960
H	3.13000	1.10390	2.65110
C	-0.50370	-0.00020	-4.38290
H	-0.86140	0.37750	-5.33000
C	-1.08370	-4.11000	2.08350
H	-1.59040	-5.05430	2.22310
C	3.11070	-2.36280	-0.35840
H	3.30120	-3.26160	-0.95500
H	2.81310	-2.68990	0.64060
C	-0.93420	0.39750	-3.11470
C	1.59030	-1.87340	-2.10250
H	2.03850	-2.70400	-2.65530
C	4.31940	1.51990	-1.13930
H	4.90690	2.40320	-1.41850
H	4.29640	0.84830	-2.00300
C	4.38600	-1.52040	-0.29690
H	5.21630	-2.14930	0.05900
H	4.63670	-1.21210	-1.31460
C	4.31700	-0.57770	1.95940
H	5.25890	-0.19140	2.37040
H	4.32390	-1.65820	2.12970
C	4.97890	0.82910	0.06120
H	6.01750	0.56650	-0.19210
H	5.01920	1.55190	0.88020
C	0.01130	-3.61710	2.77670
H	0.55950	-4.09640	3.57590
C	-4.80680	1.23480	-0.86010

H	-5.67210	1.85970	-0.58970
H	-5.09940	0.65060	-1.73640
C	-4.43530	0.88660	1.54060
H	-5.30810	0.57060	2.12880
H	-4.49950	1.97520	1.45300
C	-5.09340	-0.99250	0.09490
H	-6.13550	-0.90830	-0.24780
H	-5.11820	-1.45120	1.08710
C	-2.02540	1.29050	-2.78330
H	-2.43590	1.91990	-3.57920
Cd	-1.53470	-0.32730	-0.20990

References

(1) Rosenau, C. P.; Jelier, B. J.; Gossert, A. D.; Togni, A. Exposing the Origins of Irreproducibility in Fluorine NMR Spectroscopy. *Angew. Chem. Int. Ed.* **2018**, *57* (30), 9528–9533. DOI: 10.1002/anie.201802620.

(2) CrysAlis PRO. CrysAlisPro: Rigaku Oxford Diffraction 1.171.43.104a.

(3) Sheldrick, G. M. *SHELXT* – Integrated Space-Group and Crystal-Structure Determination. *Acta Crystallogr A Found Adv* **2015**, *71* (1), 3–8. DOI: 10.1107/S2053273314026370.

(4) Sheldrick, G. M. Crystal Structure Refinement with *SHELXL*. *Acta Crystallogr C Struct Chem* **2015**, *71* (1), 3–8. DOI: 10.1107/S2053229614024218.

(5) Dolomanov, O. V.; Bourhis, L. J.; Gildea, R. J.; Howard, J. A. K.; Puschmann, H. *OLEX2*: A Complete Structure Solution, Refinement and Analysis Program. *J. Appl. Crystallogr.* **2009**, *42* (2), 339–341. DOI: 10.1107/S0021889808042726.

(6) Neese, F. Software Update: The ORCA Program System—Version 6.0. *Wiley Interdiscip. Rev. Comput. Mol. Sci.* **2025**, *15* (2), e70019. DOI: 10.1002/wcms.70019.

(7) Bannwarth, C.; Caldeweyher, E.; Ehlert, S.; Hansen, A.; Pracht, P.; Seibert, J.; Spicher, S.; Grimme, S. Extended Tight-binding Quantum Chemistry Methods. *WIREs Comput. Mol. Sci.* **2021**, *11* (2), 1–49. DOI: 10.1002/wcms.1493.

(8) Bannwarth, C.; Ehlert, S.; Grimme, S. GFN2-xTB—An Accurate and Broadly Parametrized Self-Consistent Tight-Binding Quantum Chemical Method with Multipole Electrostatics and Density-Dependent Dispersion Contributions. *J. Chem. Theory Comput.* **2019**, *15* (3), 1652–1671. DOI: 10.1021/acs.jctc.8b01176.

(9) Caldeweyher, E.; Bannwarth, C.; Grimme, S. Extension of the D3 Dispersion Coefficient Model. *J. Chem. Phys.* **2017**, *147* (3), 034112. DOI: 10.1063/1.4993215.

(10) Caldeweyher, E.; Ehlert, S.; Hansen, A.; Neugebauer, H.; Spicher, S.; Bannwarth, C.; Grimme, S. A Generally Applicable Atomic-Charge Dependent London Dispersion Correction. *J. Chem. Phys.* **2019**, *150* (15), 154122. DOI: 10.1063/1.5090222.

(11) Caldeweyher, E.; Mewes, J.-M.; Ehlert, S.; Grimme, S. Extension and Evaluation of the D4 London-Dispersion Model for Periodic Systems. *Phys. Chem. Chem. Phys.* **2020**, *22* (16), 8499–8512. DOI: 10.1039/D0CP00502A.

(12) Najibi, A.; Goerigk, L. DFT-D4 Counterparts of Leading Meta-generalized-gradient Approximation and Hybrid Density Functionals for Energetics and Geometries. *J. Comput. Chem.* **2020**, *41* (30), 2562–2572. DOI: 10.1002/jcc.26411.

(13) Goerigk, L.; Hansen, A.; Bauer, C.; Ehrlich, S.; Najibi, A.; Grimme, S. A Look at the Density Functional Theory Zoo with the Advanced GMTKN55 Database for General Main Group Thermochemistry, Kinetics and Noncovalent Interactions. *Phys. Chem. Chem. Phys.* **2017**, *19* (48), 32184–32215. DOI: 10.1039/C7CP04913G.

(14) Weigend, F.; Ahlrichs, R. Balanced Basis Sets of Split Valence, Triple Zeta Valence and Quadruple Zeta Valence Quality for H to Rn: Design and Assessment of Accuracy. *Phys. Chem. Chem. Phys.* **2005**, *7* (18), 3297. DOI: 10.1039/b508541a.

(15) Grimme, S. Exploration of Chemical Compound, Conformer, and Reaction Space with Meta-Dynamics Simulations Based on Tight-Binding Quantum Chemical Calculations. *J. Chem. Theory Comput.* **2019**, *15* (5), 2847–2862. DOI: 10.1021/acs.jctc.9b00143.

(16) Pracht, P.; Bohle, F.; Grimme, S. Automated Exploration of the Low-Energy Chemical Space with Fast Quantum Chemical Methods. *Phys. Chem. Chem. Phys.* **2020**, *22* (14), 7169–7192. DOI: 10.1039/C9CP06869D.

(17) Lu, T.; Chen, F. Multiwfn: A Multifunctional Wavefunction Analyzer. *J. Comput. Chem.* **2012**, *33* (5), 580–592. DOI: 10.1002/jcc.22885.

(18) Lu, T. A Comprehensive Electron Wavefunction Analysis Toolbox for Chemists, Multiwfn. *J. Chem. Phys.* **2024**, *161* (8), 082503. DOI: 10.1063/5.0216272.

(19) Humphrey, W.; Dalke, A.; Schulten, K. VMD: Visual Molecular Dynamics. *J. Mol. Graphics* **1996**, *14* (1), 33–38. DOI: 10.1016/0263-7855(96)00018-5.

(20) Peters, G. M.; Winegrad, J. B.; Gau, M. R.; Imler, G. H.; Xu, B.; Ren, S.; Wayland, B. B.; Zdilla, M. J. Synthesis and Structure of 2,5-Bis[*N* -(2,6-Mesityl)Iminomethyl]Pyrrolylcobalt(II): Evidence for One-Electron-Oxidized, Redox Noninnocent Ligand Behavior. *Inorg. Chem.* **2017**, *56* (6), 3377–3385. DOI: 10.1021/acs.inorgchem.6b02898.

(21) Sukiennik, J.; Sarwa, A.; Perdek, J. P.; Siczek, M.; Szyszko, B. Tautomerism-Coupled Self-Assembly and Transformations of Iminopyrrole Metallacages. *Chem. Eur. J.* **2025**, *31* (63), e02714. DOI: 10.1002/chem.202502714.

(22) Sarwa, A.; Białońska, A.; Garbicz, M.; Szyszko, B. Plenates: Anion-Dependent Self-Assembly of the Pyrrole Cage Encapsulating Silver(I) Clusters. *Chem. Eur. J.* **2023**, *29* (12), e202203850. DOI: 10.1002/chem.202203850.
